# Supplementary material for: Randomized phase II study of daily versus alternate-day administrations of S-1 for the elderly patients with completely resected pathological stage IA (tumor diameter > 2 cm)—IIIA of non-small cell lung cancer: Setouchi Lung Cancer Group Study 1201
Source: PLoS One. 2023 May 19;18(5):e0285273. doi: 10.1371/journal.pone.0285273 (PMC10198543; doi:10.1371/journal.pone.0285273)
Supplement: S1 Fig — Arm A: alternate-day administration, Arm B: daily administration. (PDF) [file pone.0285273.s002.pdf]

S1 Fig

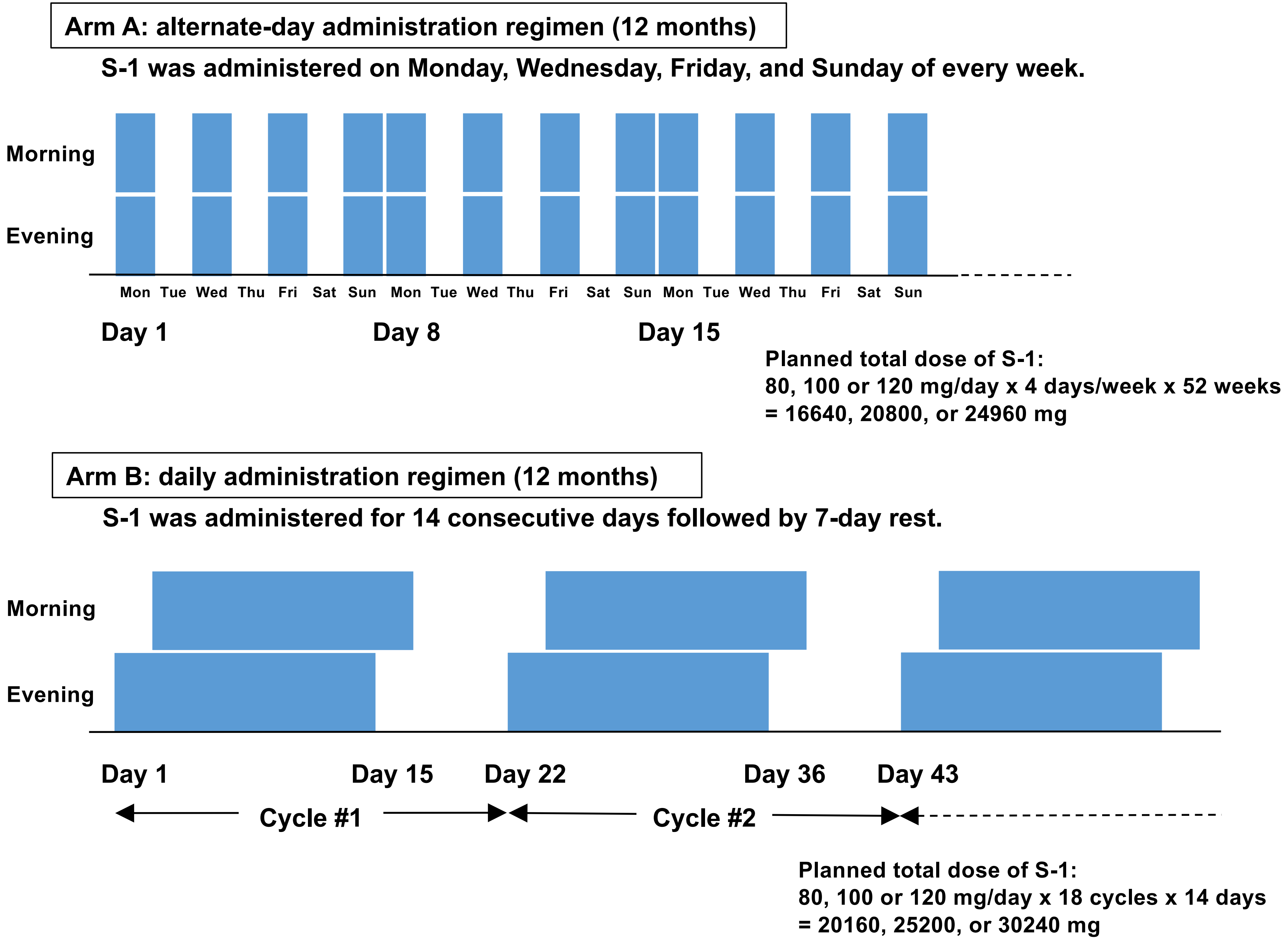
Arm A: alternate-day administration regimen (12 months)
S-1 was administered on Monday, Wednesday, Friday, and Sunday of every week.
Morning
Evening
Mon Tue Wed Thu Fri Sat Sun Mon Tue Wed Thu Fri Sat Sun Mon Tue Wed Thu Fri Sat Sun
Day 1
Day 8
Day 15
Planned total dose of S-1:
80, 100 or 120 mg/day x 4 days/week x 52 weeks
= 16640, 20800, or 24960 mg
Arm B: daily administration regimen (12 months)
S-1 was administered for 14 consecutive days followed by 7-day rest.
Morning
Evening
Day 1
Day 15
Day 22
Day 36
Day 43
Cycle #1
Cycle #2
Planned total dose of S-1:
80, 100 or 120 mg/day x 18 cycles x 14 days
= 20160, 25200, or 30240 mg
