# Supplementary figures and images for: Randomized phase II study of daily versus alternate-day administrations of S-1 for the elderly patients with completely resected pathological stage IA (tumor diameter > 2 cm)—IIIA of non-small cell lung cancer: Setouchi Lung Cancer Group Study 1201
Source: PLoS One. 2023 May 19;18(5):e0285273. doi: 10.1371/journal.pone.0285273 (PMC10198543; doi:10.1371/journal.pone.0285273)

S2 Fig

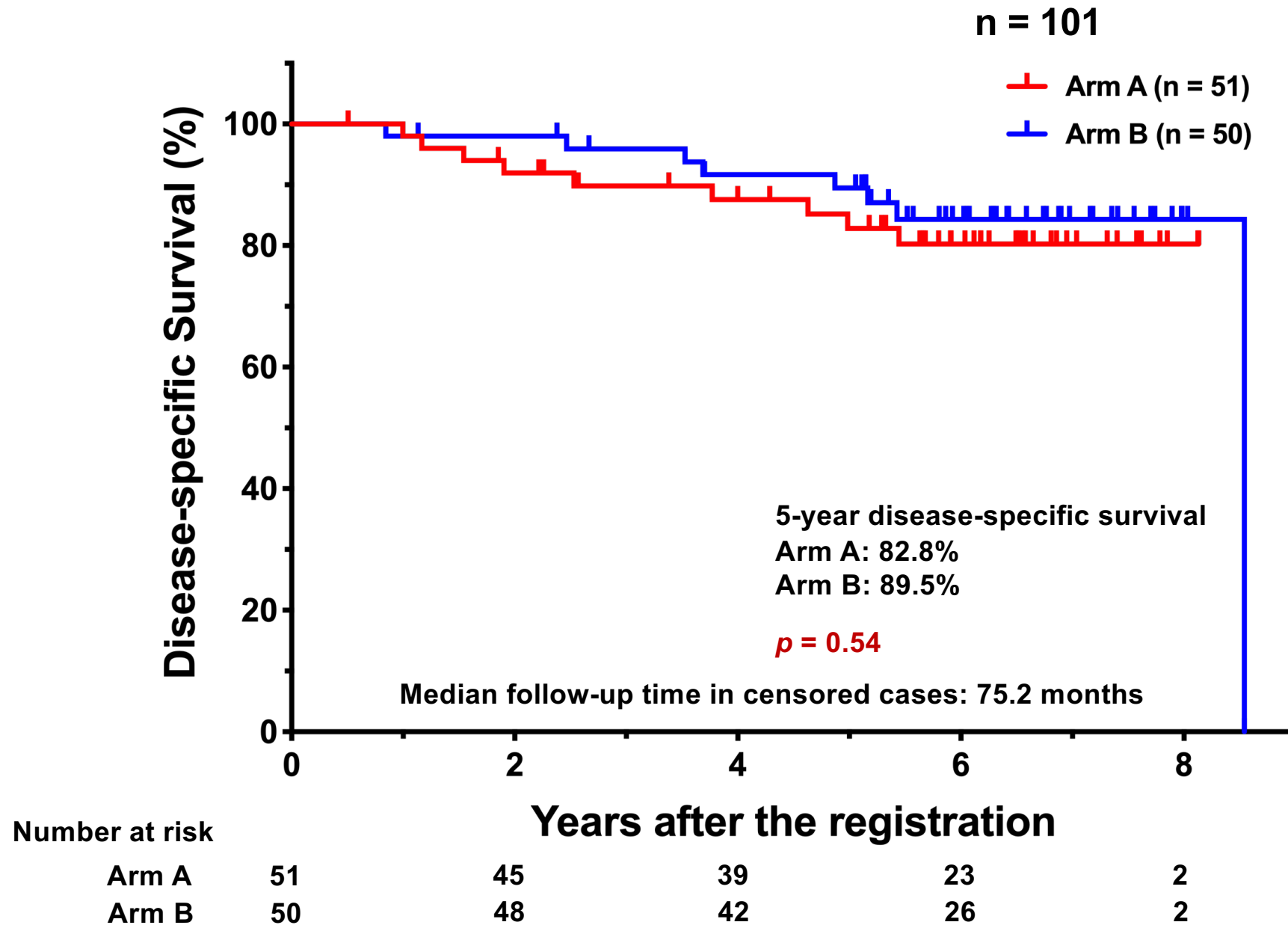

Supplement: S2 Fig — (PDF) [file pone.0285273.s003.pdf]

S5 Fig A

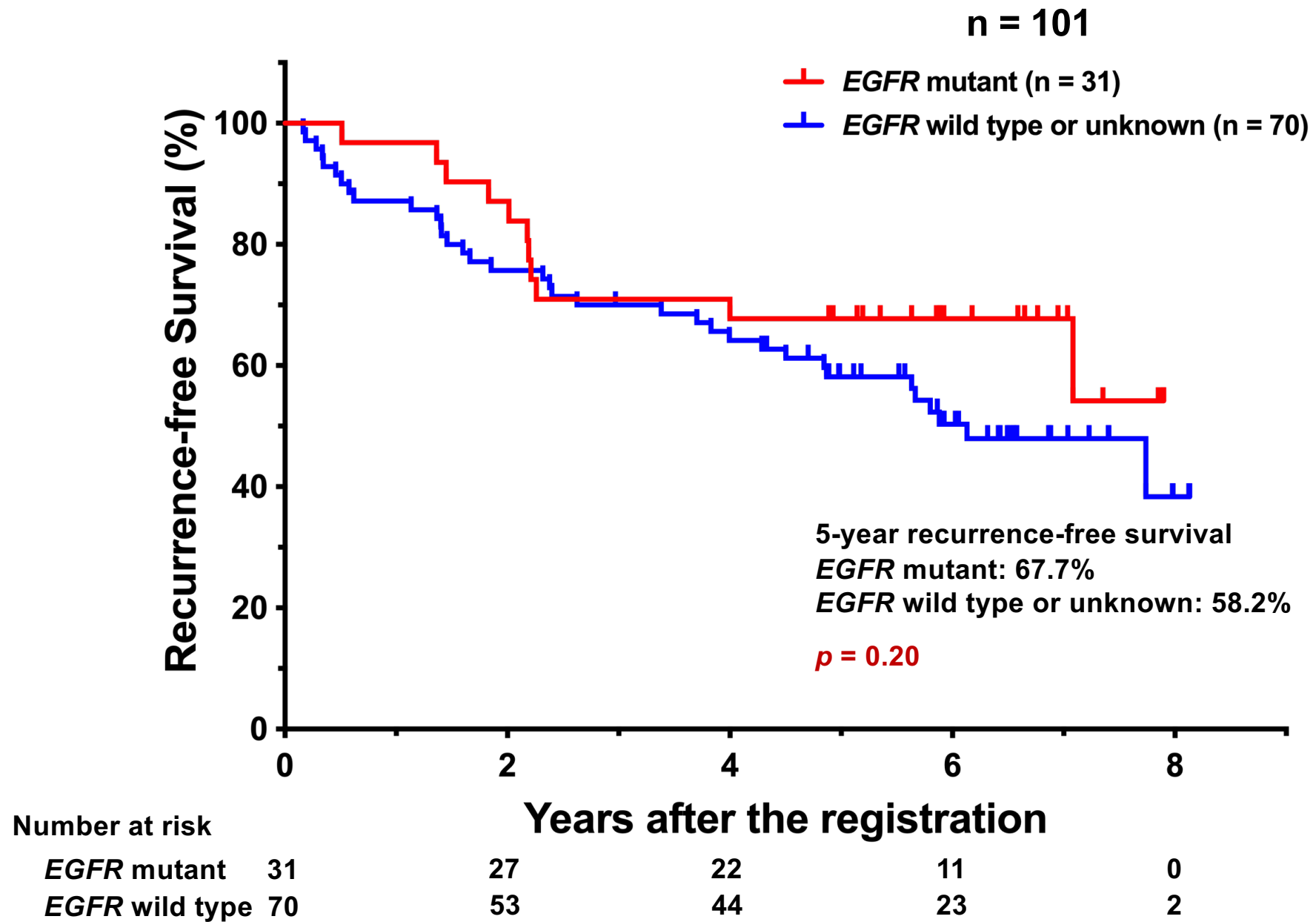

S5 Fig B

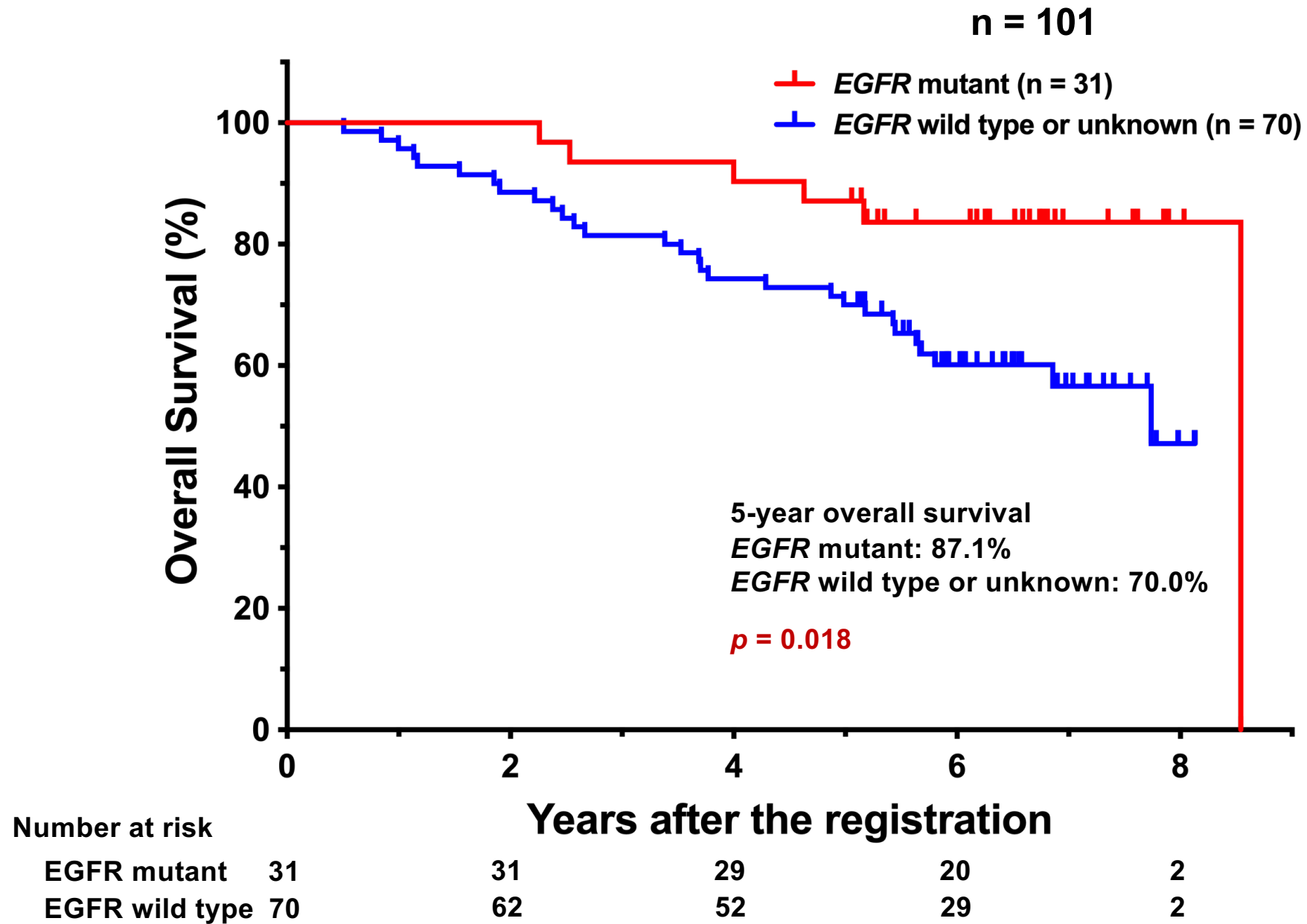

Supplement: S5 Fig — (A) Recurrence-free survival (RFS) of the patients by EGFR mutational status. (B) Overall survival (OS) of the patients by EGFR mutational status. (PDF) [file pone.0285273.s006.pdf]

S7 Fig A

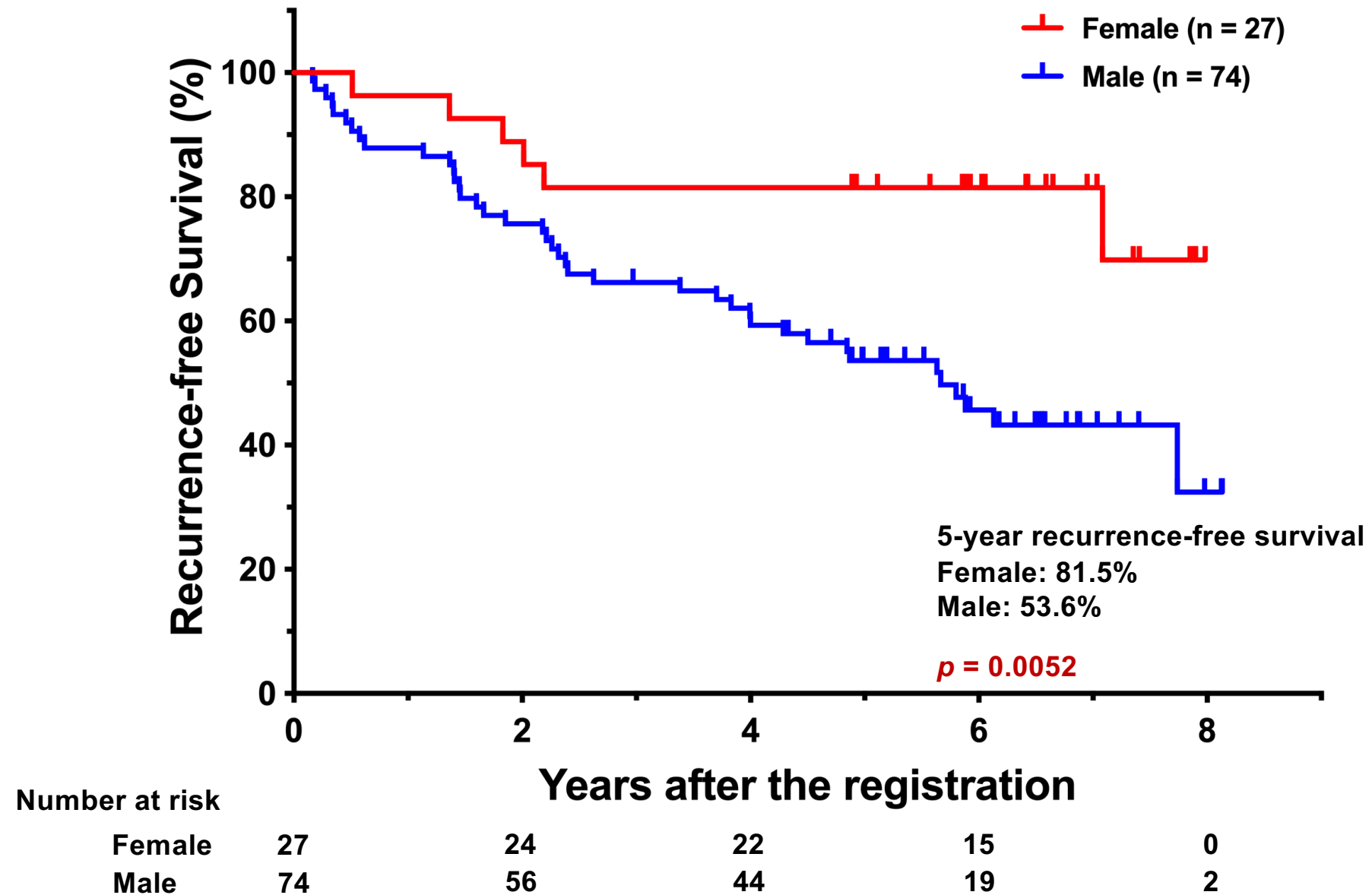

S7 Fig B

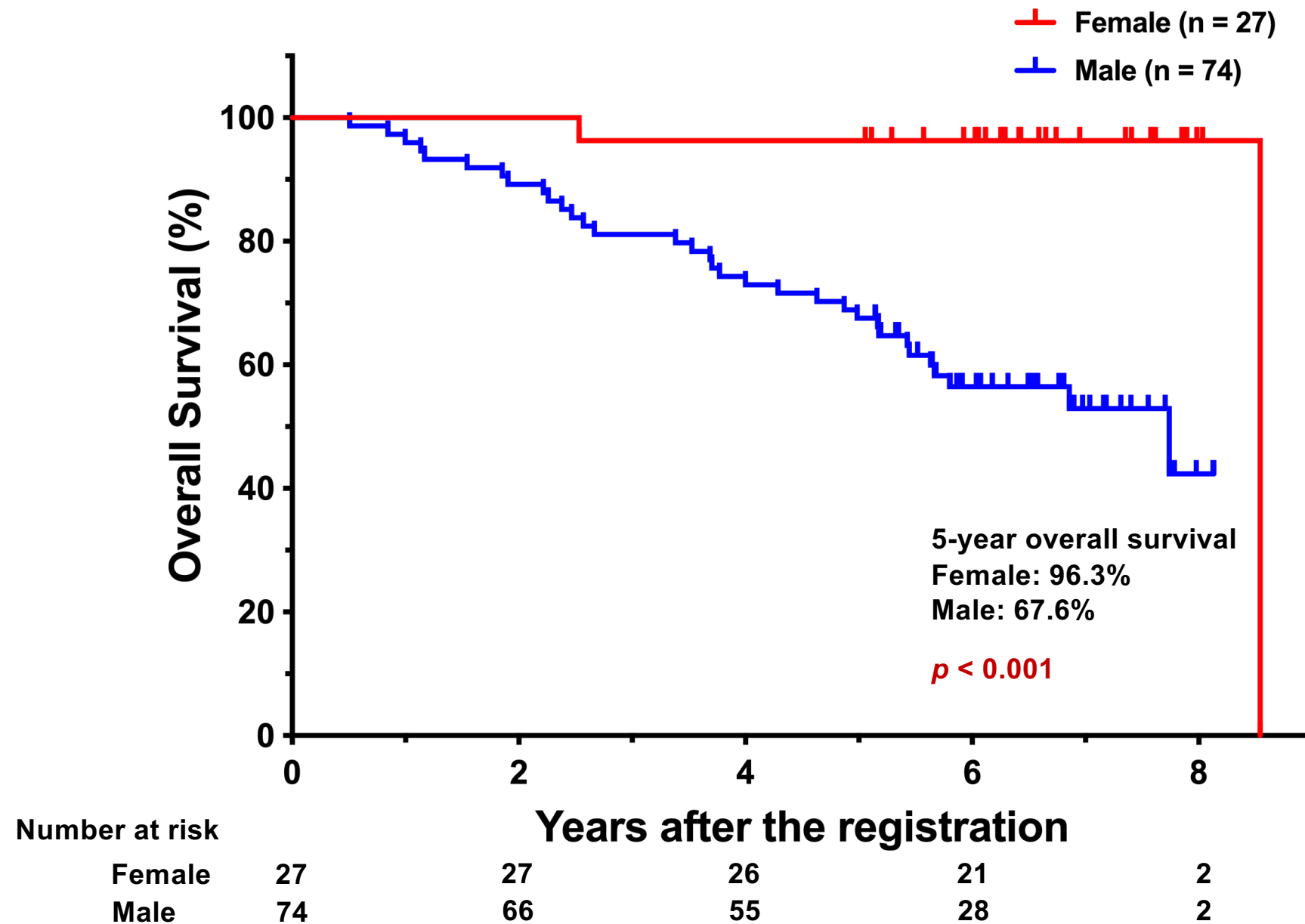

Supplement: S7 Fig — (A) Recurrence-free survival (RFS) of the patients by sex. (B) Overall survival (OS) of the patients by sex. (PDF) [file pone.0285273.s008.pdf]
