# Supplementary material for: Randomized phase II study of daily versus alternate-day administrations of S-1 for the elderly patients with completely resected pathological stage IA (tumor diameter > 2 cm)—IIIA of non-small cell lung cancer: Setouchi Lung Cancer Group Study 1201
Source: PLoS One. 2023 May 19;18(5):e0285273. doi: 10.1371/journal.pone.0285273 (PMC10198543; doi:10.1371/journal.pone.0285273)
Supplement: S3 Fig — (A) Recurrence-free survival (RFS) of the patients by completion of S-1 administration at 6 months. (B) Recurrence-free survival (RFS) of the patients by completion of S-1 administration at 6 months excluding the recurrent cases within 6 months (6 cases were excluded). (C) Overall survival (OS) of the patients by completion of S-1 administration at 6 months. (D) Overall survival (OS) of the patients by completion of S-1 administration at 6 months excluding the recurrent cases within 6 months (6 cases were excluded). (E) Recurrence-free survival (RFS) of the patients with the completion of S-1 administration at 6 months. (F) Overall survival (OS) of the patients with the completion of S-1 administration at 6 months. (G) Recurrence-free survival (RFS) of the patients without the completion of S-1 administration at 6 months. (H) Recurrence-free survival (RFS) of the patients without the completion of S-1 administration at 6 months excluding the recurrent cases within 6 months (6 cases were excluded). (I) Overall survival (OS) of the patients without the completion of S-1 administration at 6 months. (J) Overall survival (OS) of the patients without the completion of S-1 administration at 6 months excluding the recurrent cases within 6 months (6 cases were excluded). (K) Disease-specific survival (DSS) of the patients with the completion of S-1 administration at 6 months. (L) Disease-specific survival (DSS) of the patients without the completion of S-1 administration at 6 months. (M) Disease-specific survival (DSS) of the patients without the completion of S-1 administration at 6 months excluding the recurrent cases within 6 months (6 cases were excluded). (PDF) [file pone.0285273.s004.pdf]

S3 Fig A

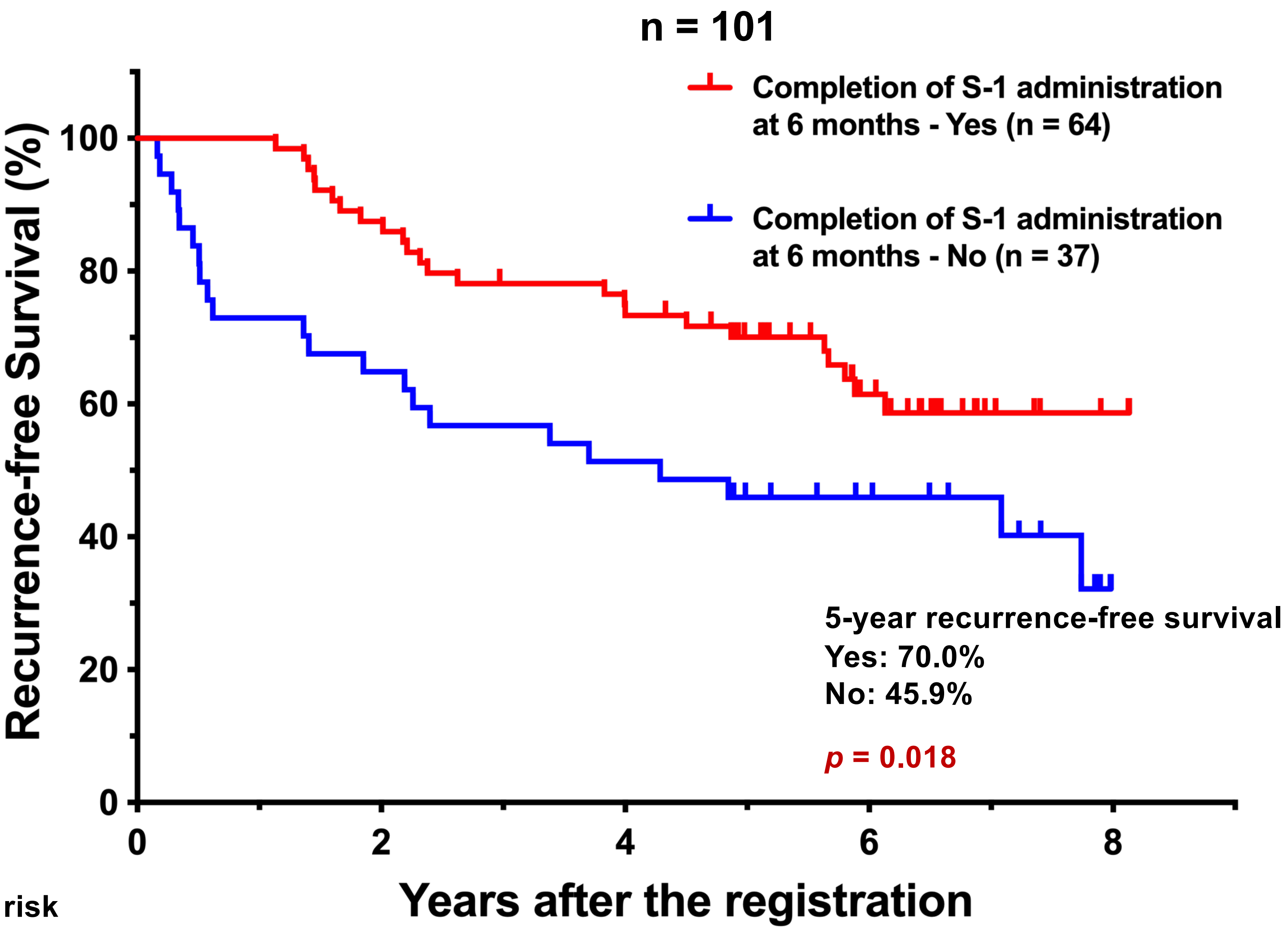

Number at risk

Yes	64	56	47	23	2
No	37	24	19	11	0

S3 Fig B

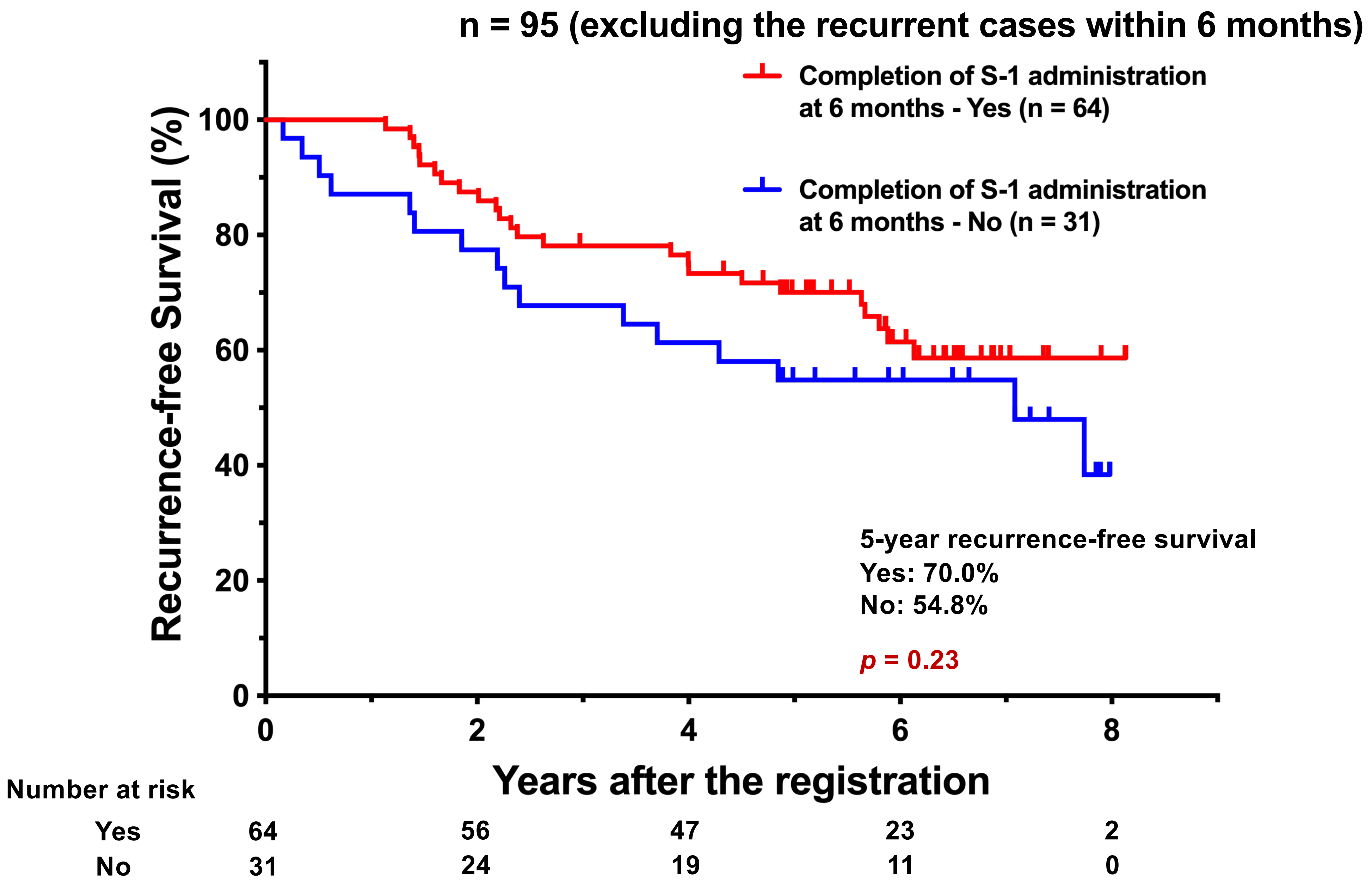
n = 95 (excluding the recurrent cases within 6 months)
Completion of S-1 administration at 6 months - Yes (n = 64)
Completion of S-1 administration at 6 months - No (n = 31)
Recurrence-free Survival (%)
100
80
60
40
20
0
0
2
4
6
8
Years after the registration
5-year recurrence-free survival
Yes: 70.0%
No: 54.8%
p = 0.23
Number at risk
Yes 64 56 47 23 2
No 31 24 19 11 0

S3 Fig C

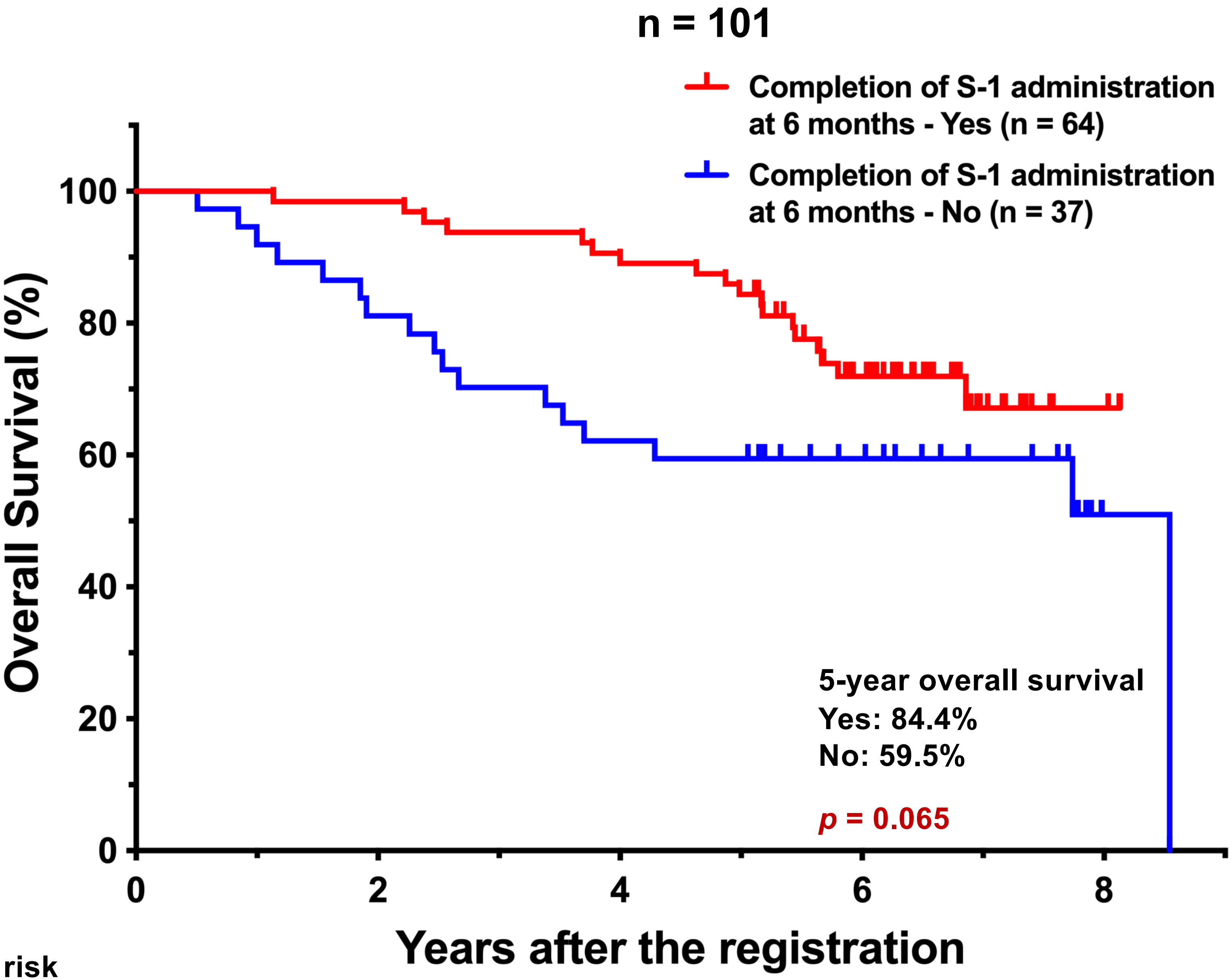

Number at risk

Yes	64	63	58	33	3
No	37	30	23	16	1

S3 Fig D

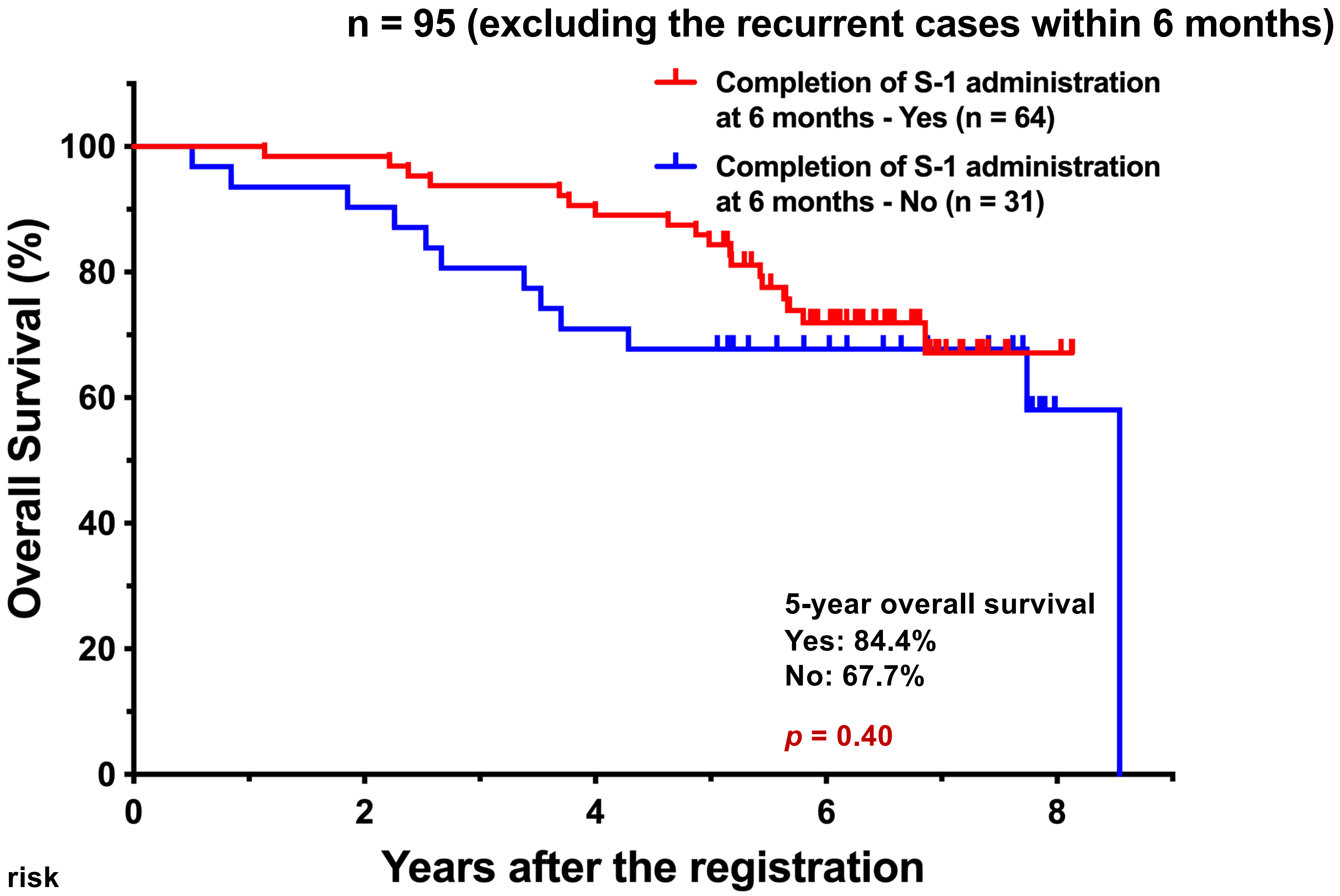

Number at risk

	0	2	4	6	8
Yes	64	63	58	33	3
No	31	28	22	15	1

S3 Fig E

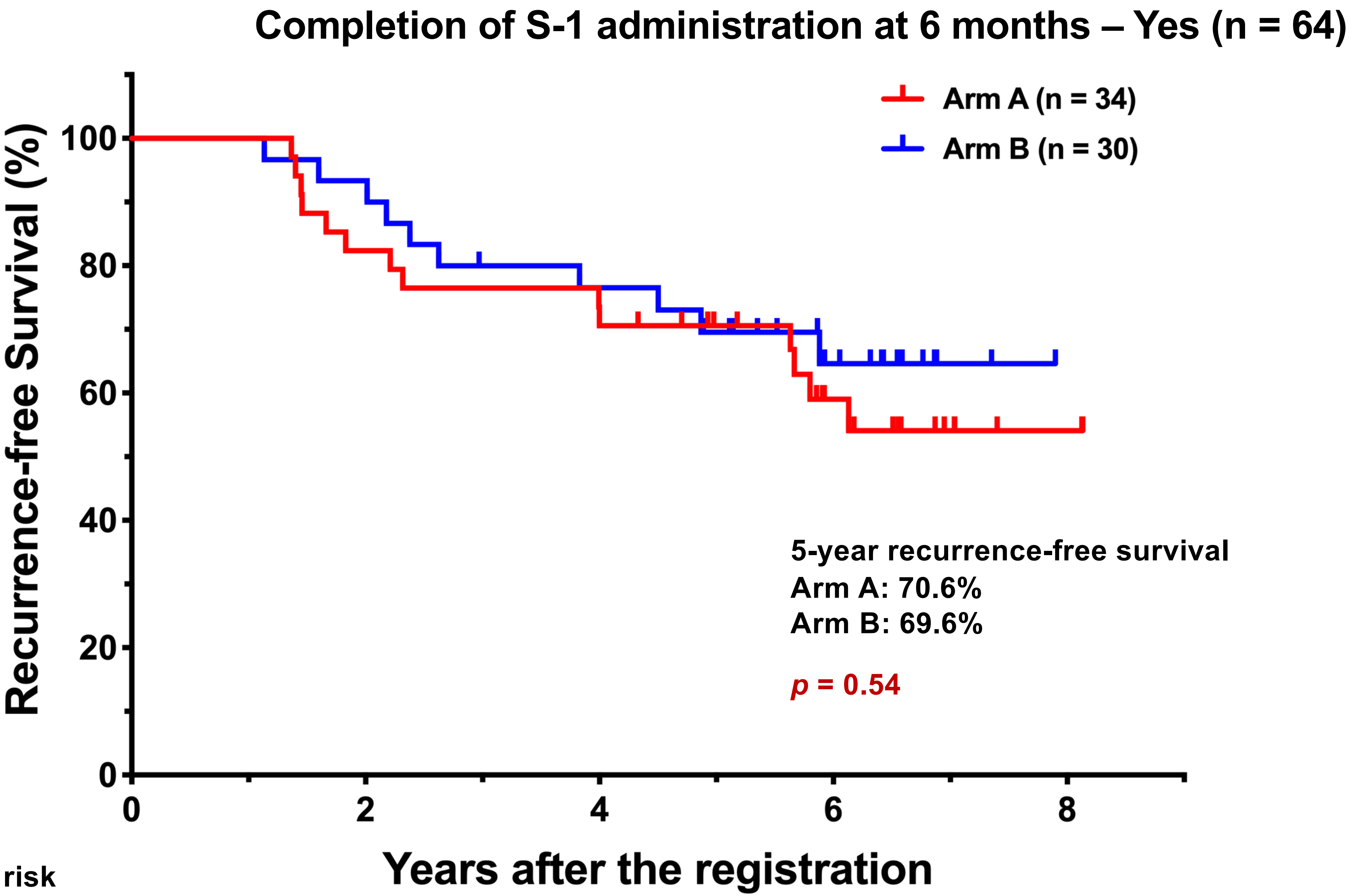

Number at risk

Arm A	34	28	25	12	2
Arm B	30	28	22	11	0

S3 Fig F

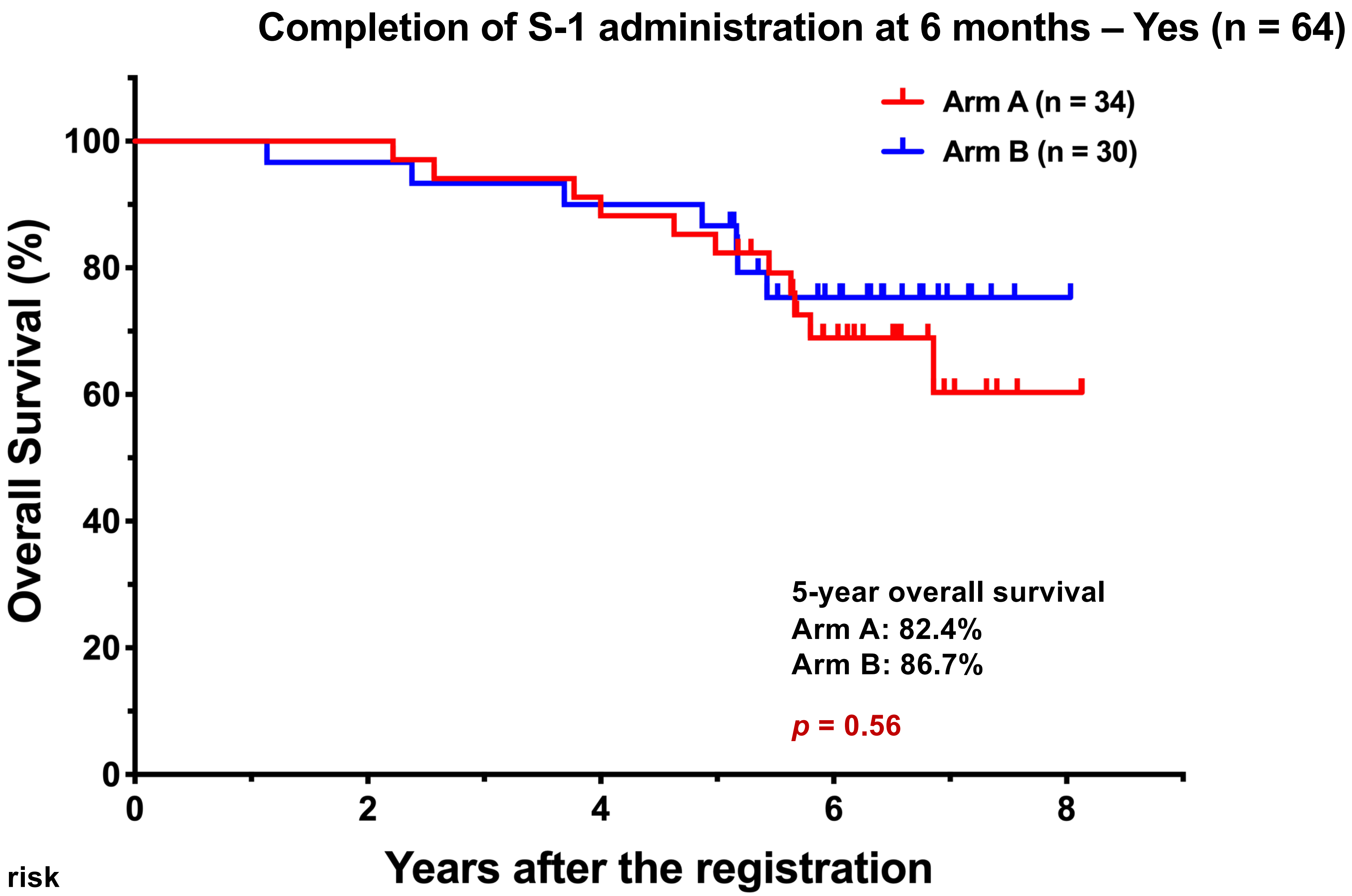

Number at risk					
Arm A	34	34	31	17	2
Arm B	30	29	27	16	1

S3 Fig G

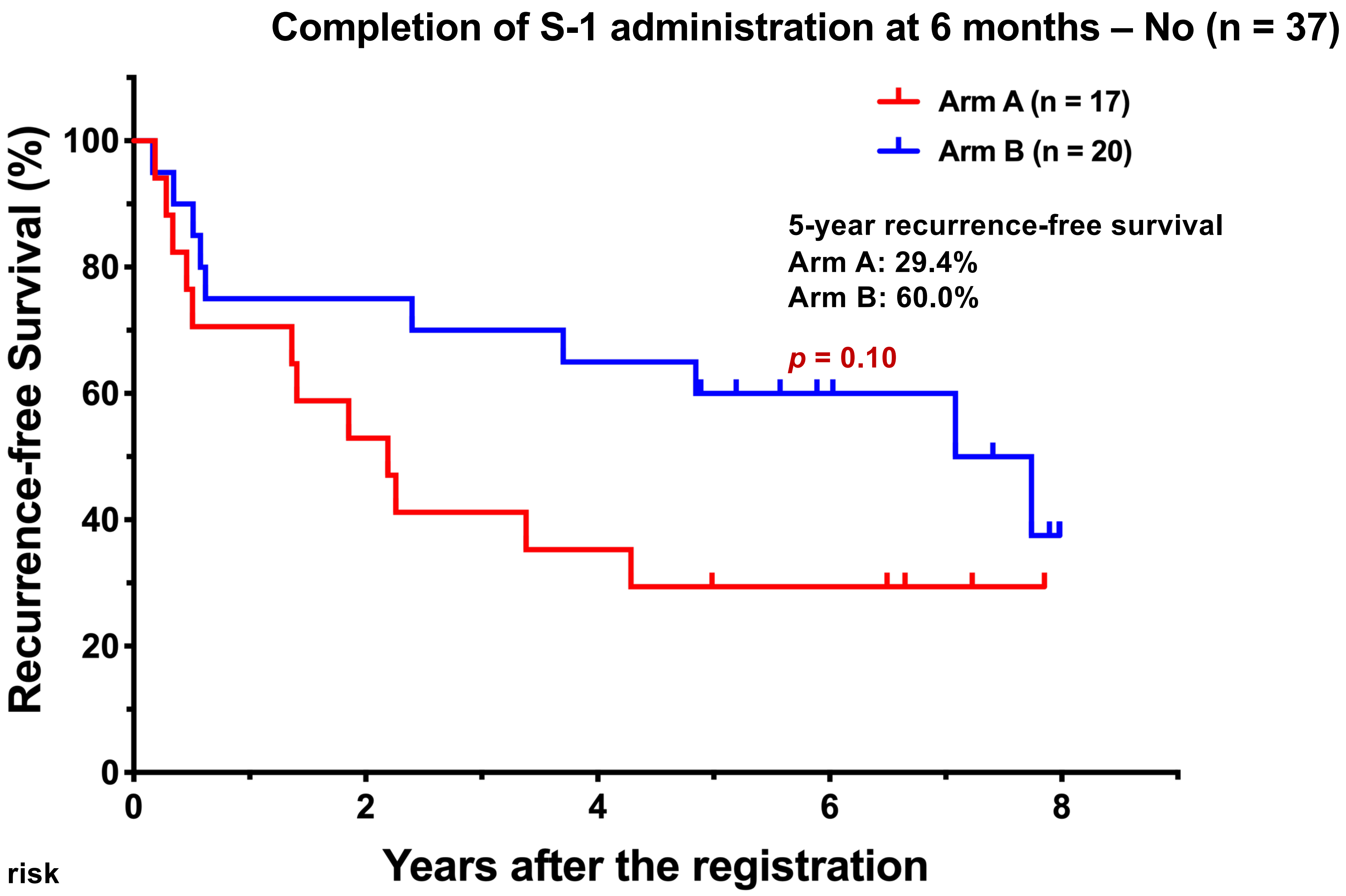

Number at risk					
Arm A	17	9	6	4	0
Arm B	20	15	13	7	0

S3 Fig H

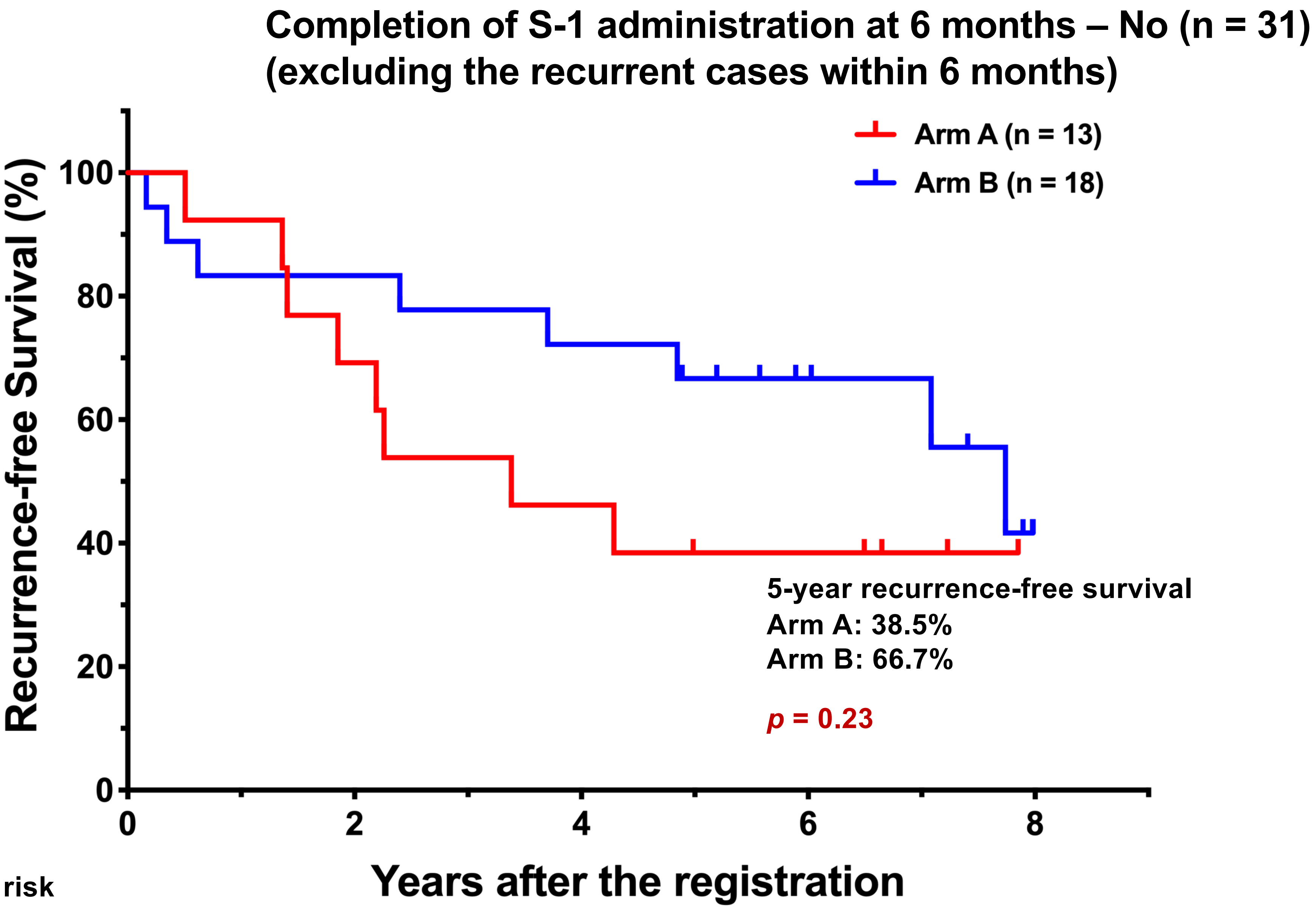

Number at risk					
Arm A	13	9	6	4	0
Arm B	18	15	13	7	0

S3 Fig I

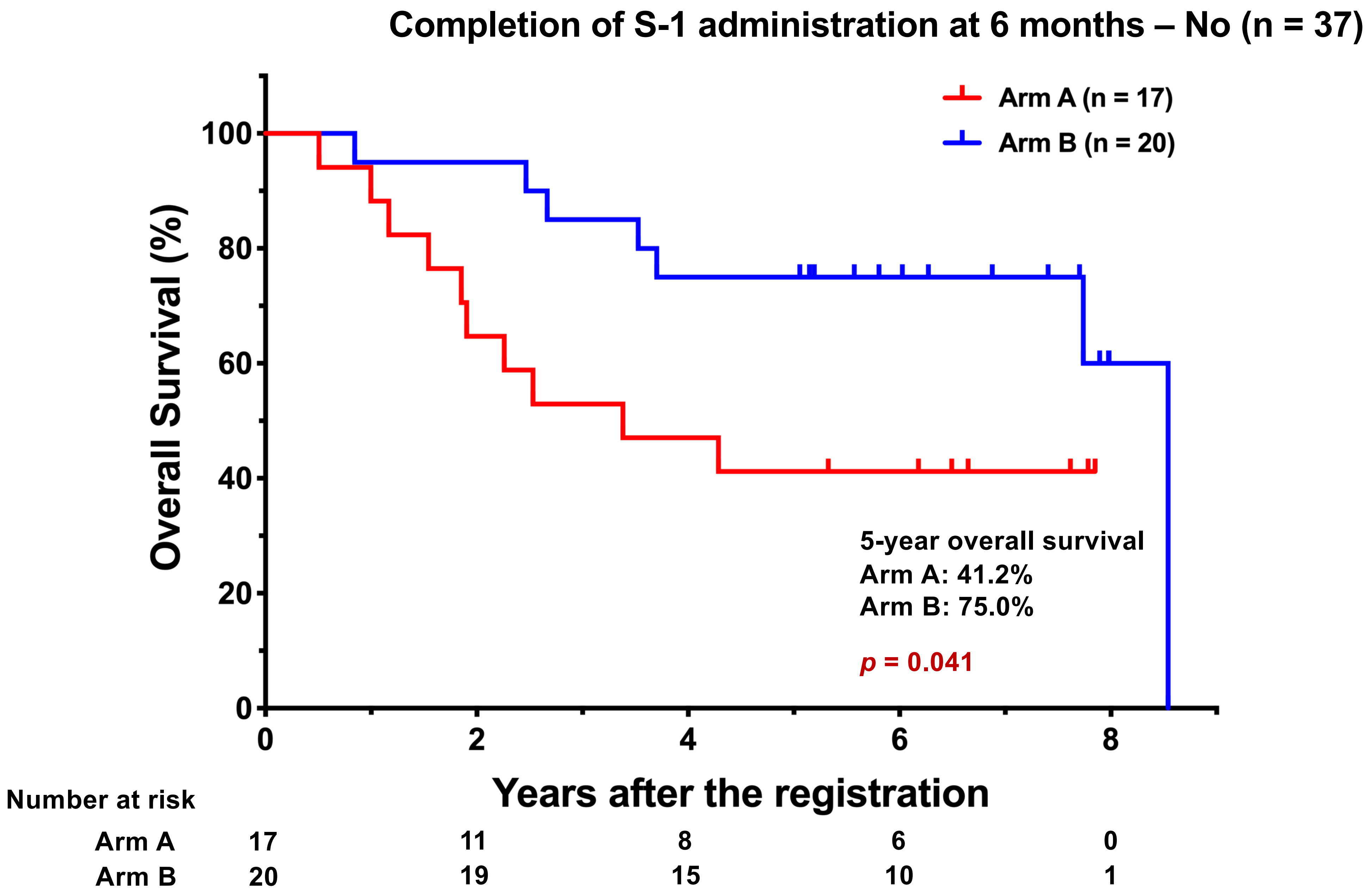
Completion of S-1 administration at 6 months – No (n = 37)
Arm A (n = 17)
Arm B (n = 20)
Overall Survival (%)
100
80
60
40
20
0
0
2
4
6
8
Years after the registration
5-year overall survival
Arm A: 41.2%
Arm B: 75.0%
p = 0.041
Number at risk
Arm A 17 11 8 6 0
Arm B 20 19 15 10 1

S3 Fig J

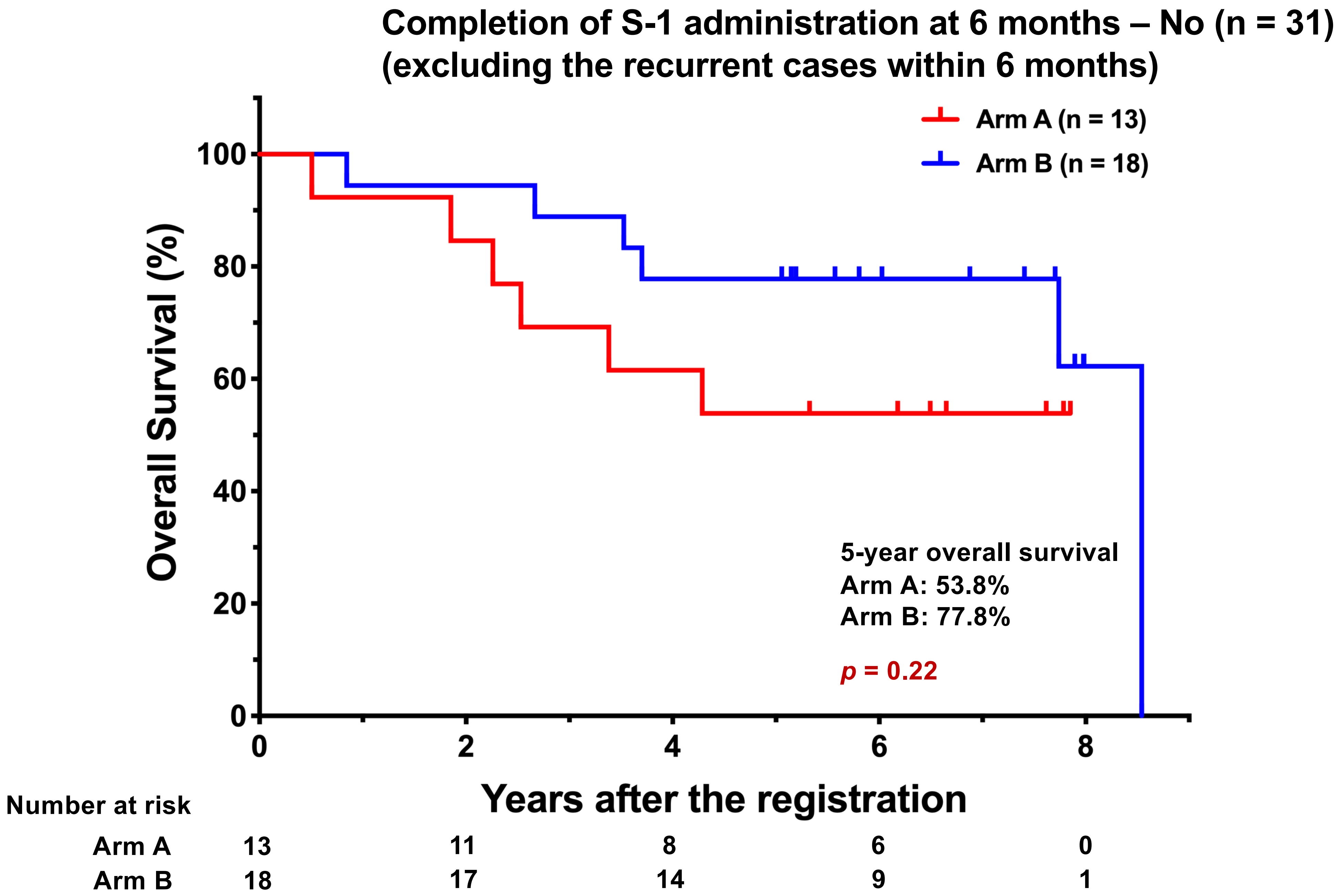

Completion of S-1 administration at 6 months – No (n = 31)
(excluding the recurrent cases within 6 months)
Arm A (n = 13)
Arm B (n = 18)
Overall Survival (%)
100
80
60
40
20
0
0
2
4
6
8
Years after the registration
5-year overall survival
Arm A: 53.8%
Arm B: 77.8%
p = 0.22
Number at risk
Arm A 13 11 8 6 0
Arm B 18 17 14 9 1

S3 Fig K

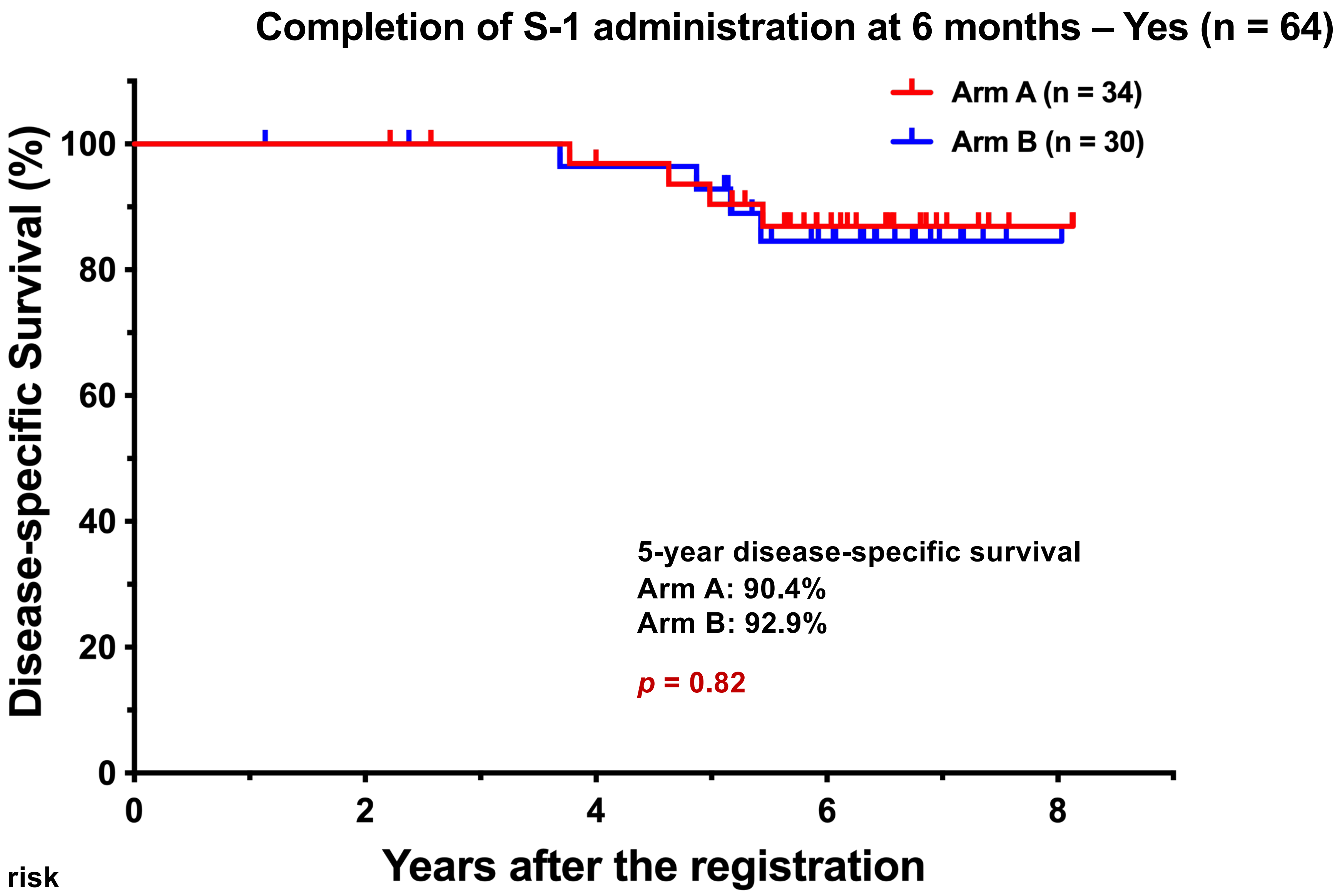

Number at risk

Arm A	34	34	31	17	2
Arm B	30	29	27	16	1

S3 Fig L

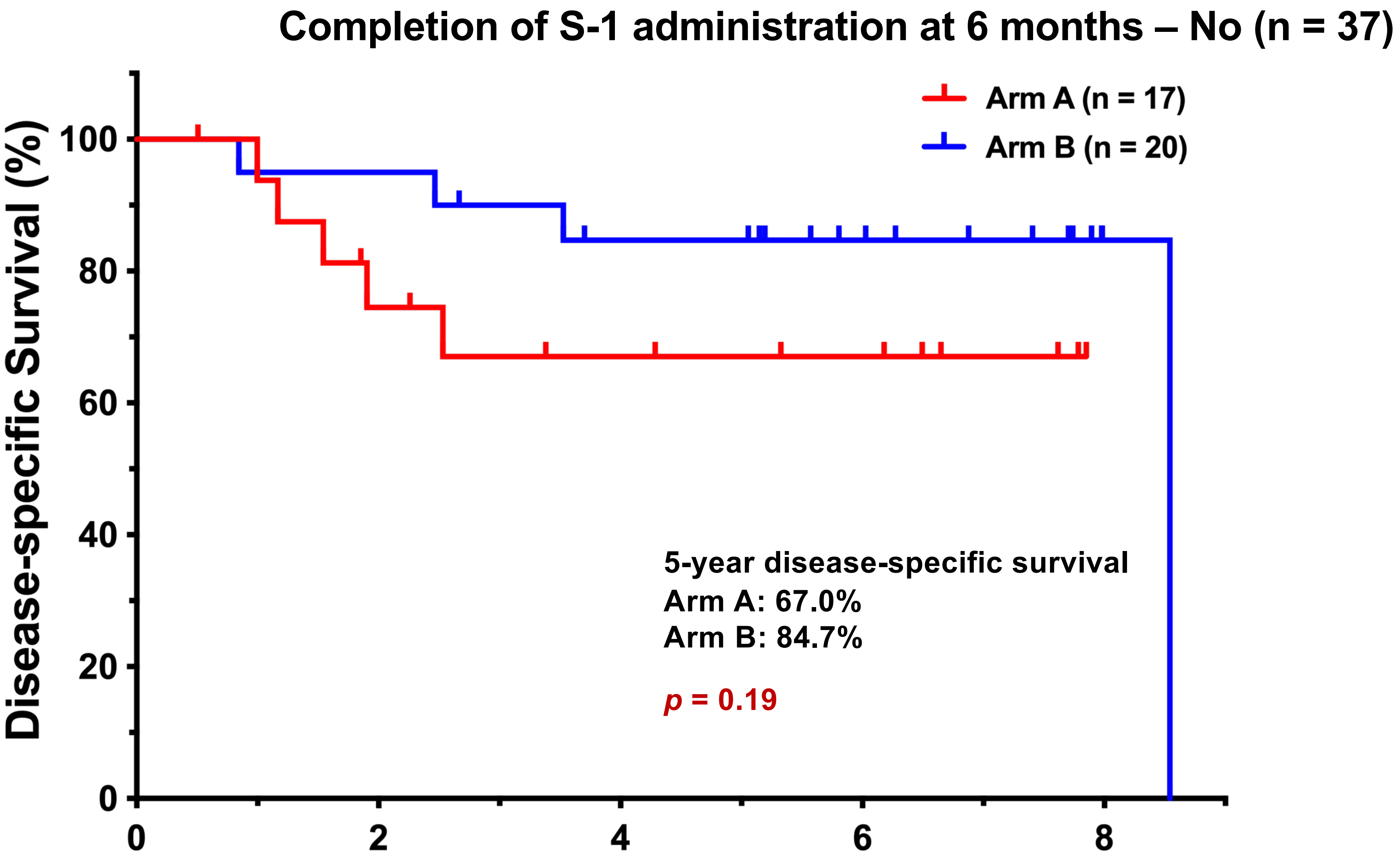

Number at risk					
Arm A	17	11	8	6	0
Arm B	20	19	15	10	1

S3 Fig M

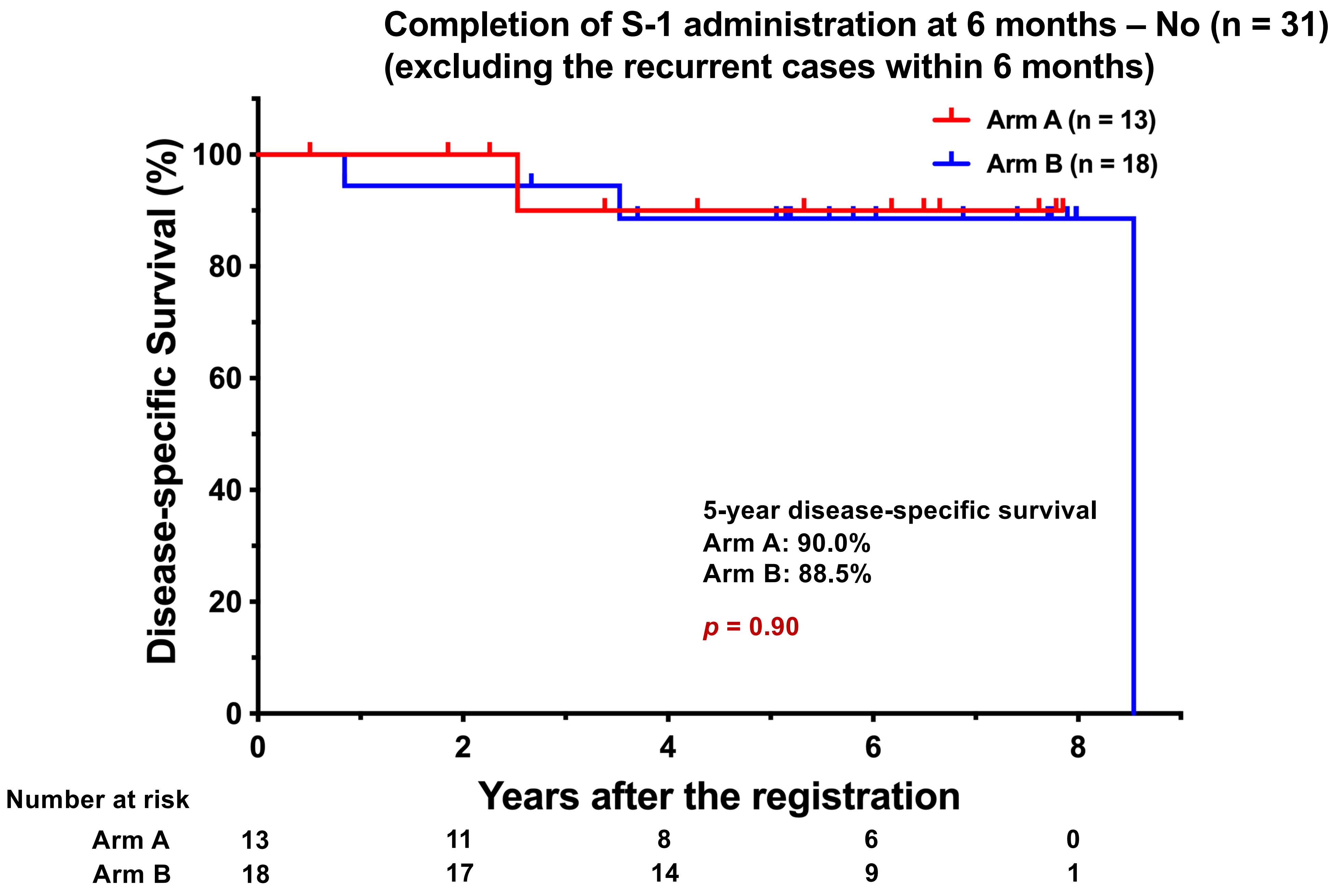
Completion of S-1 administration at 6 months – No (n = 31)
(excluding the recurrent cases within 6 months)
Arm A (n = 13)
Arm B (n = 18)
Disease-specific Survival (%)
100
80
60
40
20
0
5-year disease-specific survival
Arm A: 90.0%
Arm B: 88.5%
p = 0.90
0
2
4
6
8
Years after the registration
Number at risk
Arm A 13 11 8 6 0
Arm B 18 17 14 9 1
