# Supplementary material for: Randomized phase II study of daily versus alternate-day administrations of S-1 for the elderly patients with completely resected pathological stage IA (tumor diameter > 2 cm)—IIIA of non-small cell lung cancer: Setouchi Lung Cancer Group Study 1201
Source: PLoS One. 2023 May 19;18(5):e0285273. doi: 10.1371/journal.pone.0285273 (PMC10198543; doi:10.1371/journal.pone.0285273)
Supplement: S4 Fig — (A) Recurrence-free survival (RFS) of the patients by pStage. (B) Overall survival (OS) of the patients by pStage. (C) Recurrence-free survival (RFS) of the patients with pStage IA (T1bN0M0)/IB. (D) Recurrence-free survival (RFS) of the patients with pStage IB. (E) Overall survival (OS) of the patients with pStage IA (T1bN0M0)/IB. (F) Overall survival (OS) of the patients with pStage IB. (G) Recurrence-free survival (RFS) of the patients with pStage IIA/IIB. (H) Overall survival (OS) of the patients with pStage IIA/IIB. (I) Recurrence-free survival (RFS) of the patients with pStage IIIA. (J) Overall survival (OS) of the patients with pStage IIIA. (PDF) [file pone.0285273.s005.pdf]

S4 Fig A

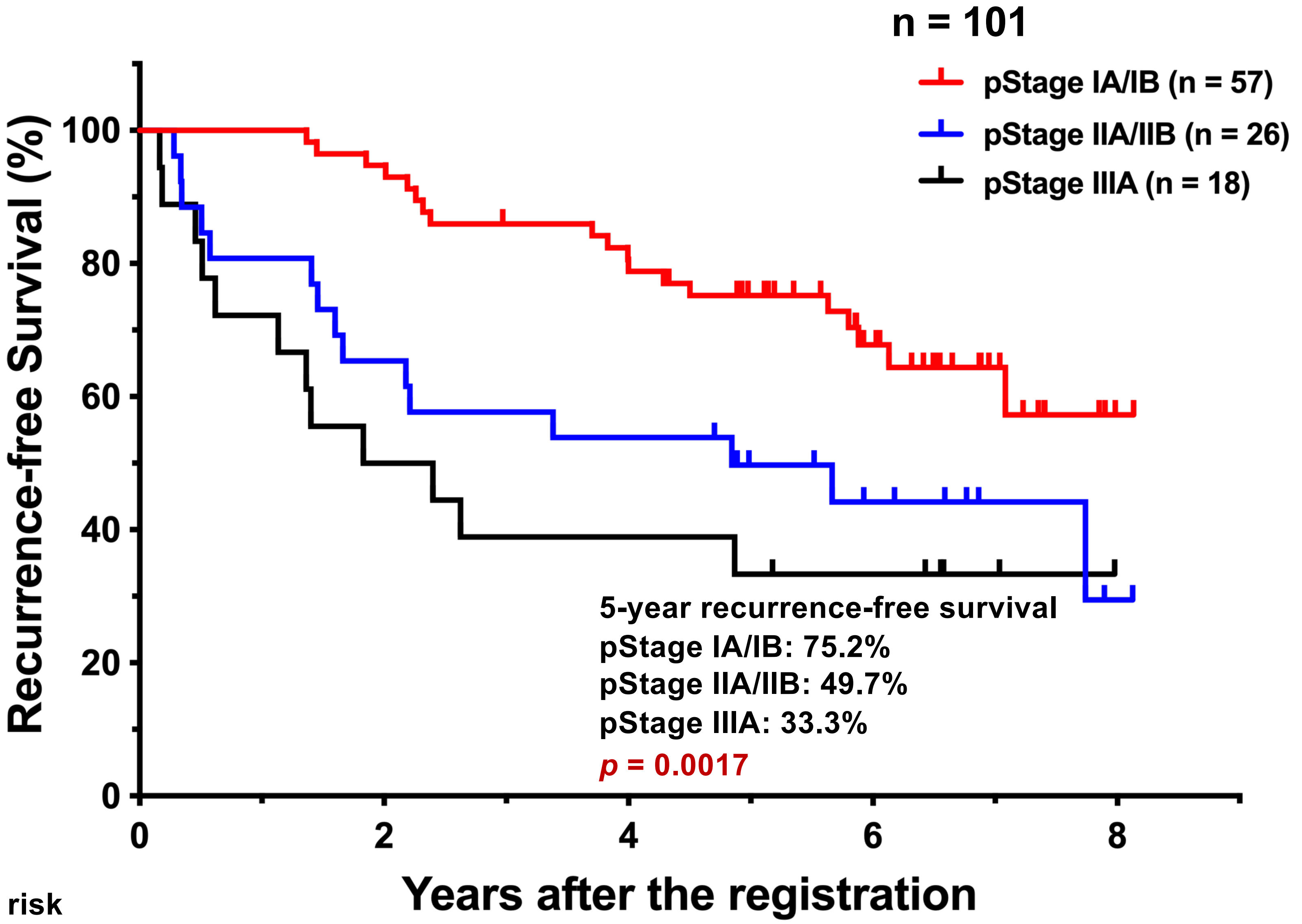

Number at risk

pStage IA/IB	57	54	45	22	1
pStage IIA/IIB	26	17	14	7	1
pStage IIIA	18	9	7	5	0

S4 Fig B

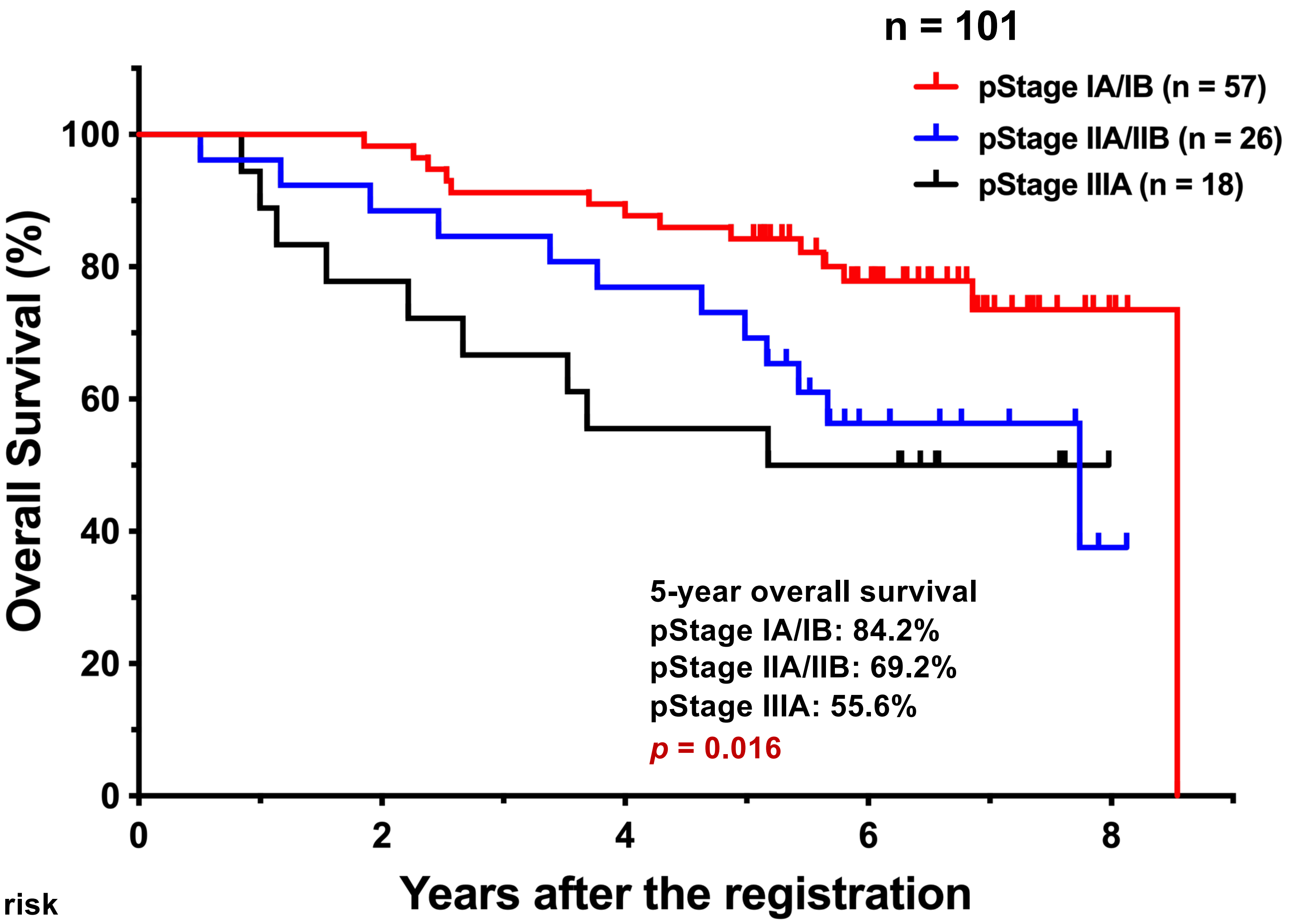

Number at risk

pStage IA/IB	57	56	51	32	3
pStage IIA/IIB	26	23	20	9	1
pStage IIIA	18	14	10	8	0

S4 Fig C

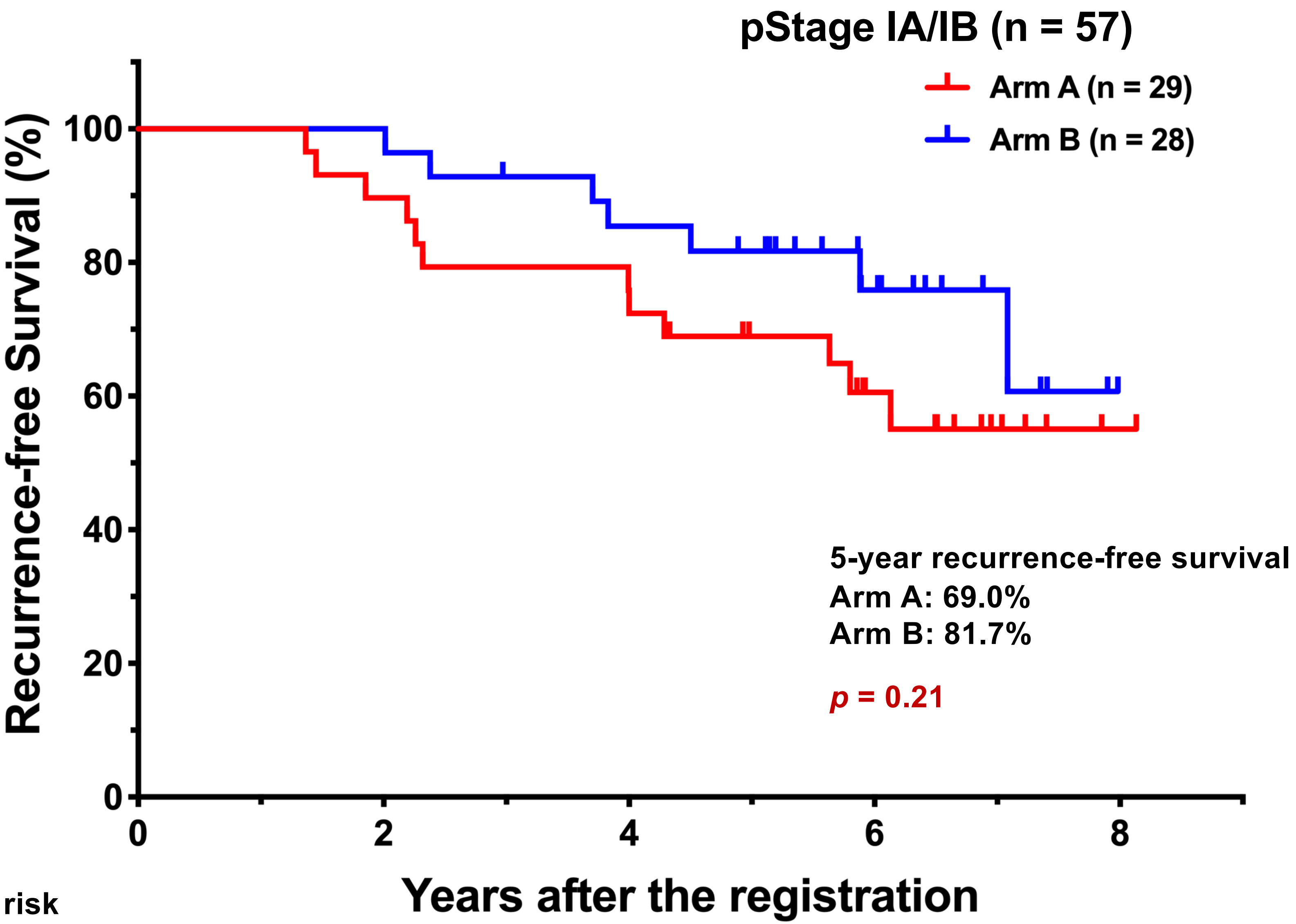

Number at risk

	0	2	4	6	8
Arm A	29	26	22	11	1
Arm B	28	28	23	11	0

S4 Fig D

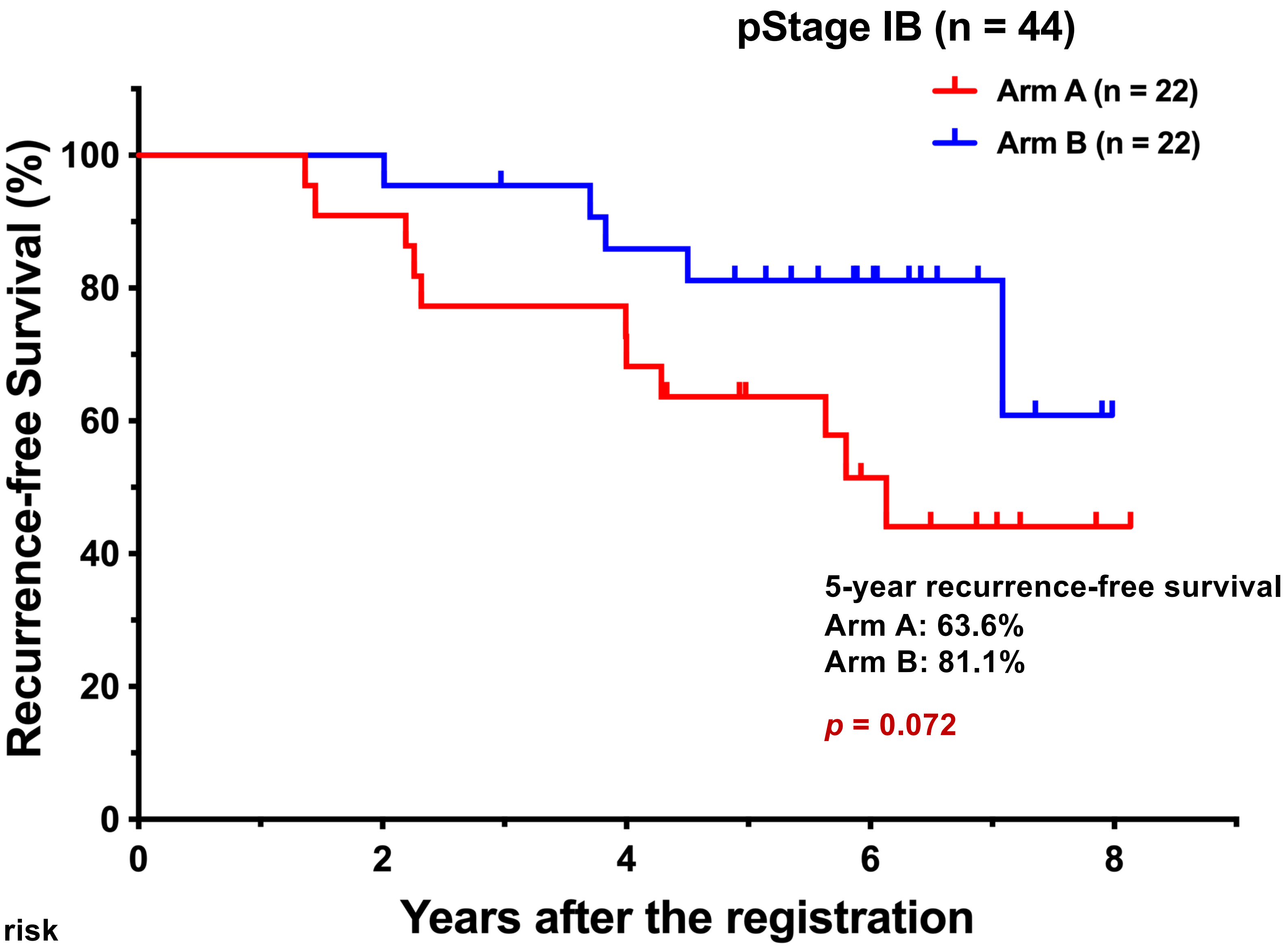

Number at risk

Arm A	22	20	16	7	1
Arm B	22	22	18	10	0

S4 Fig E

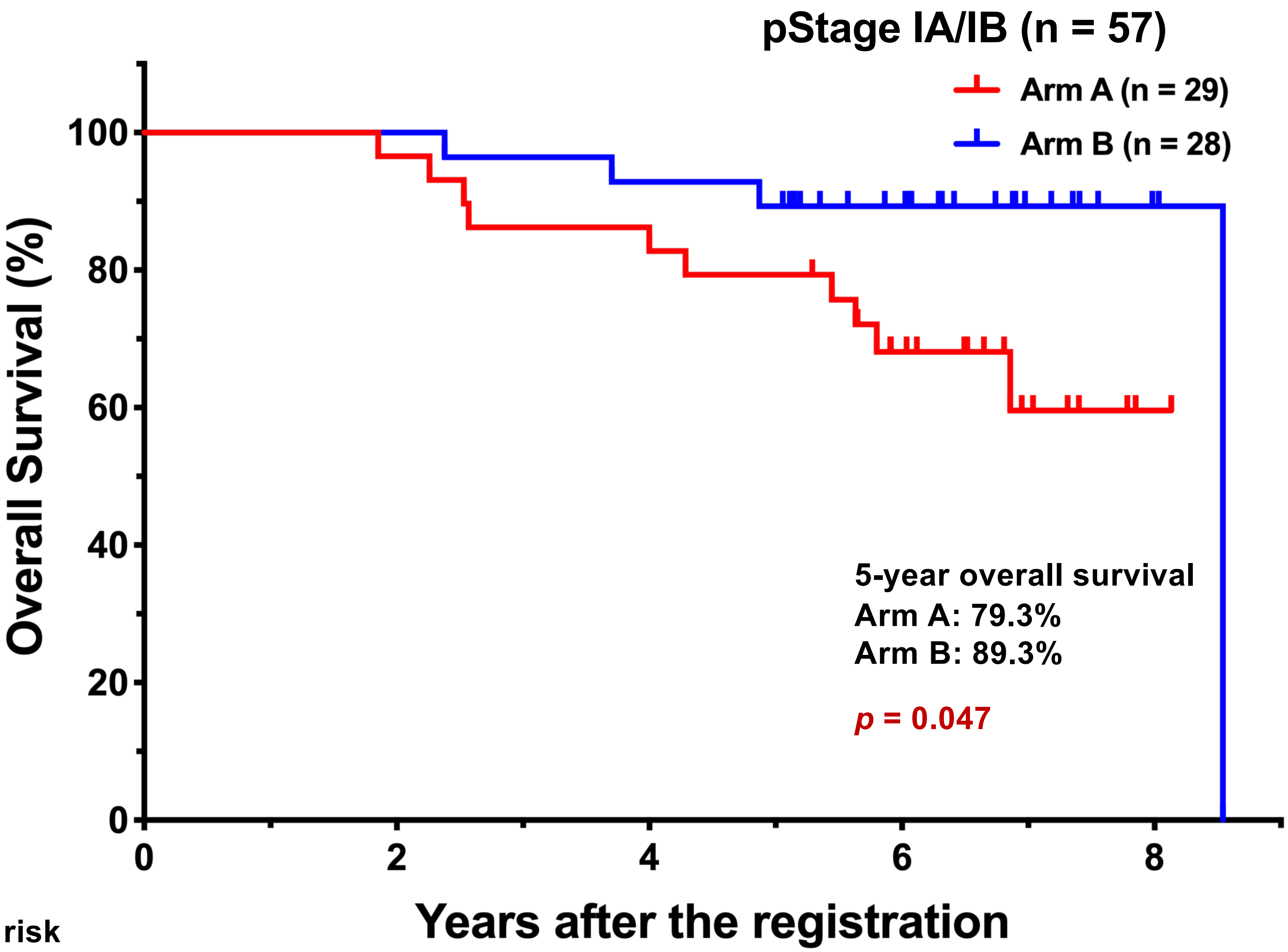

Number at risk

Arm A	29	28	25	15	1
Arm B	28	28	26	17	2

S4 Fig F

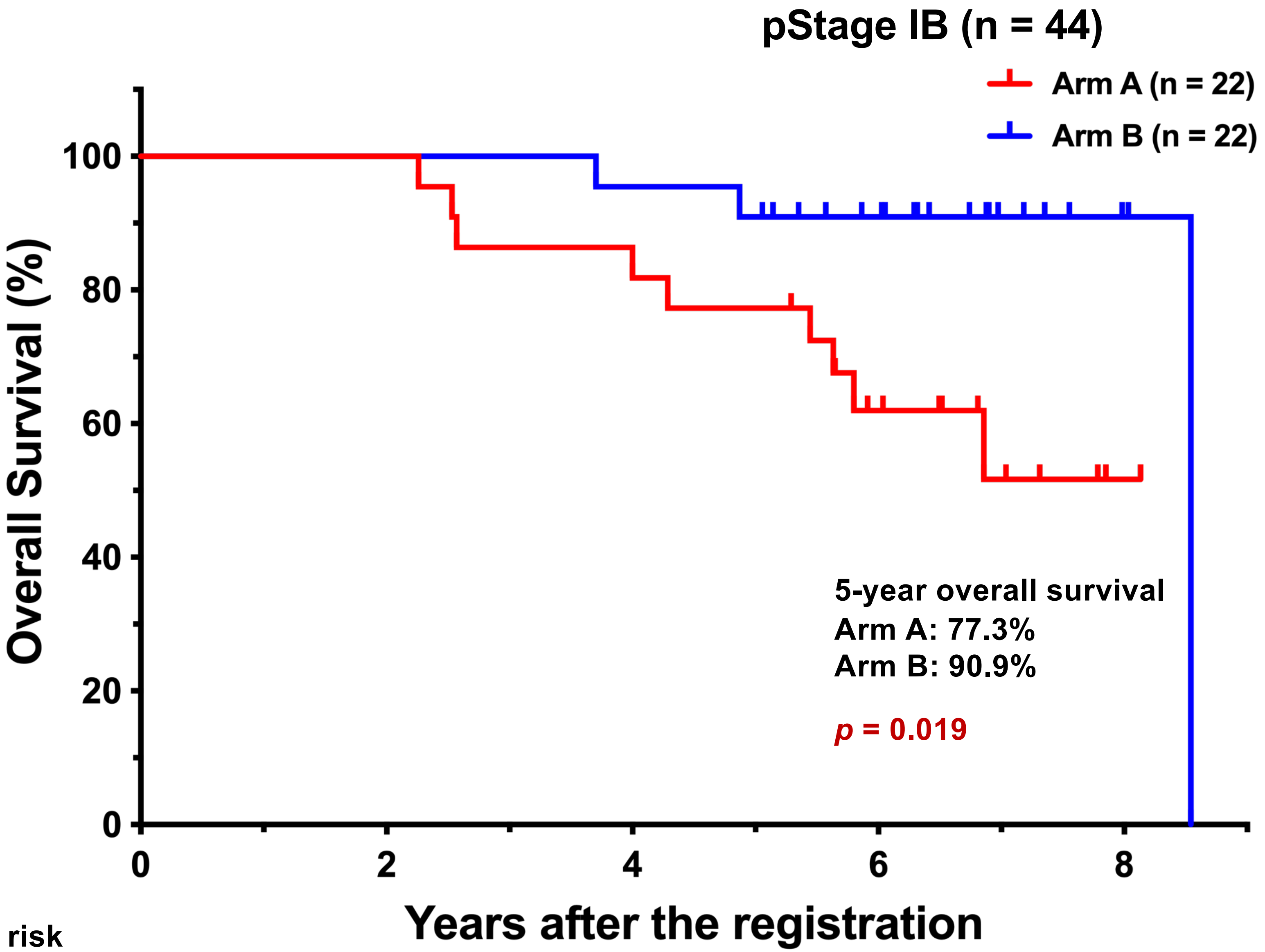

Number at risk					
Arm A	22	22	19	10	1
Arm B	22	22	21	15	2

S4 Fig G

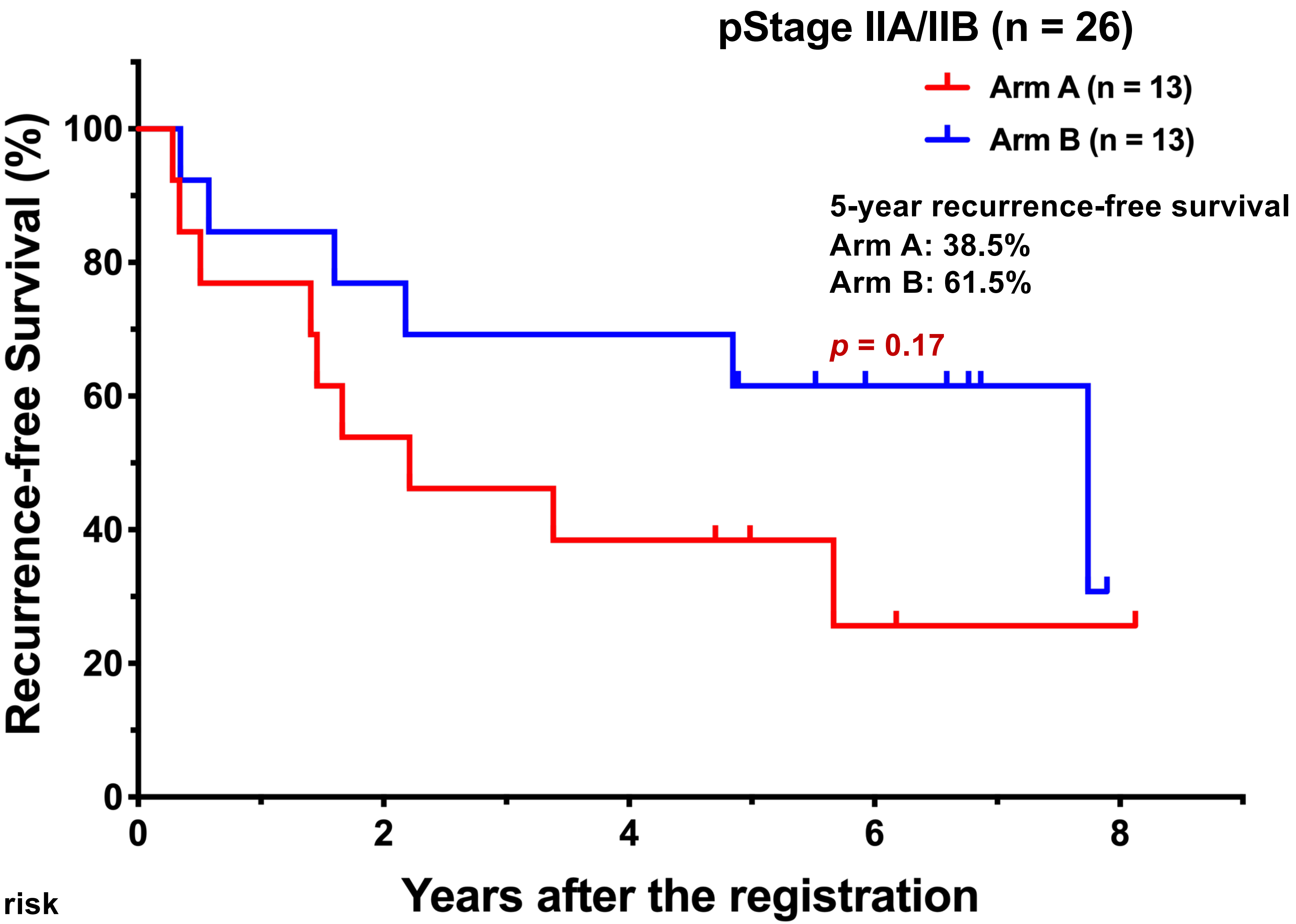

Number at risk

Arm A	13	7	5	2	1
Arm B	13	10	9	5	0

S4 Fig H

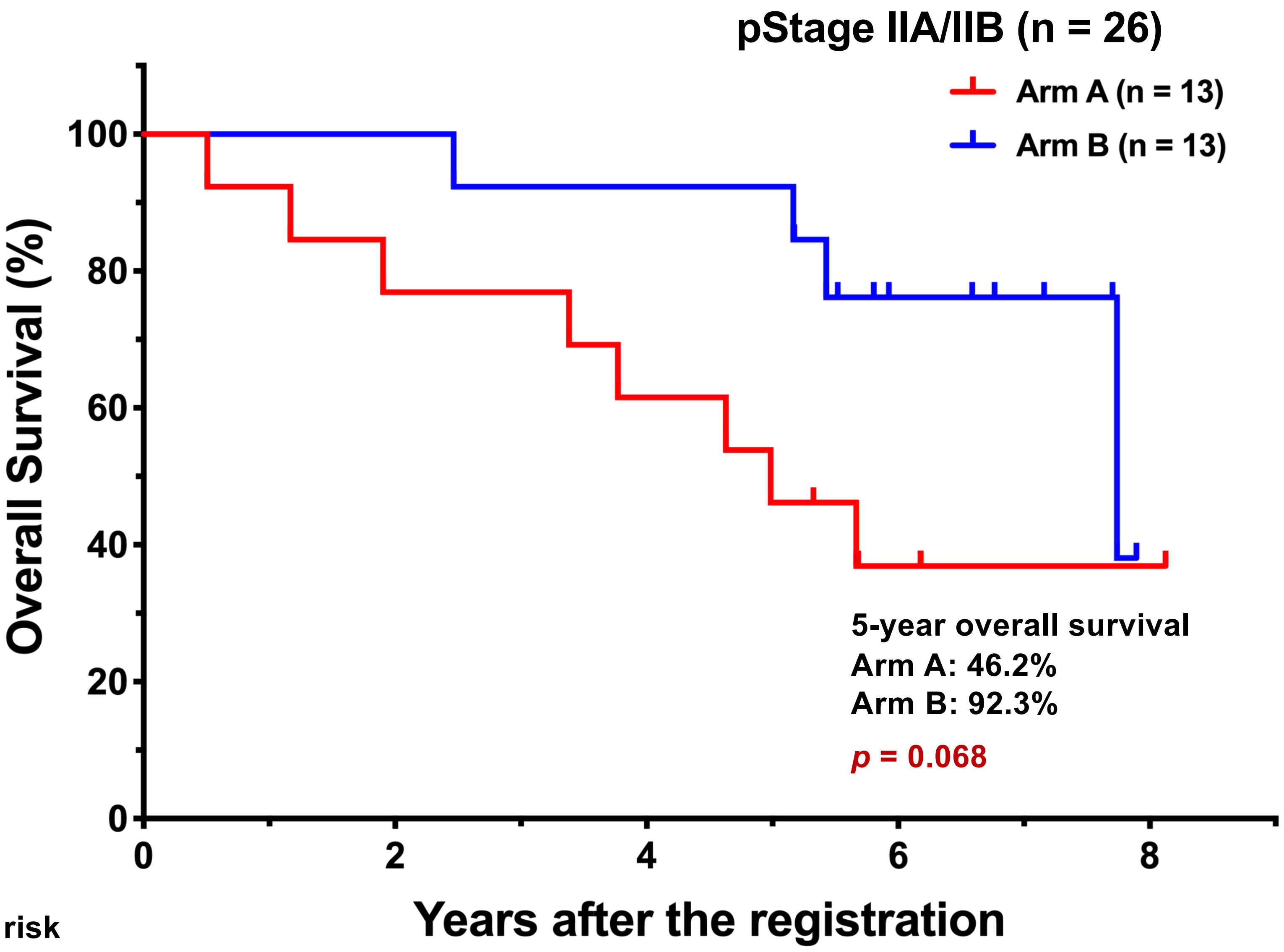

Number at risk					
Arm A	13	10	8	3	1
Arm B	13	13	12	6	0

S4 Fig I

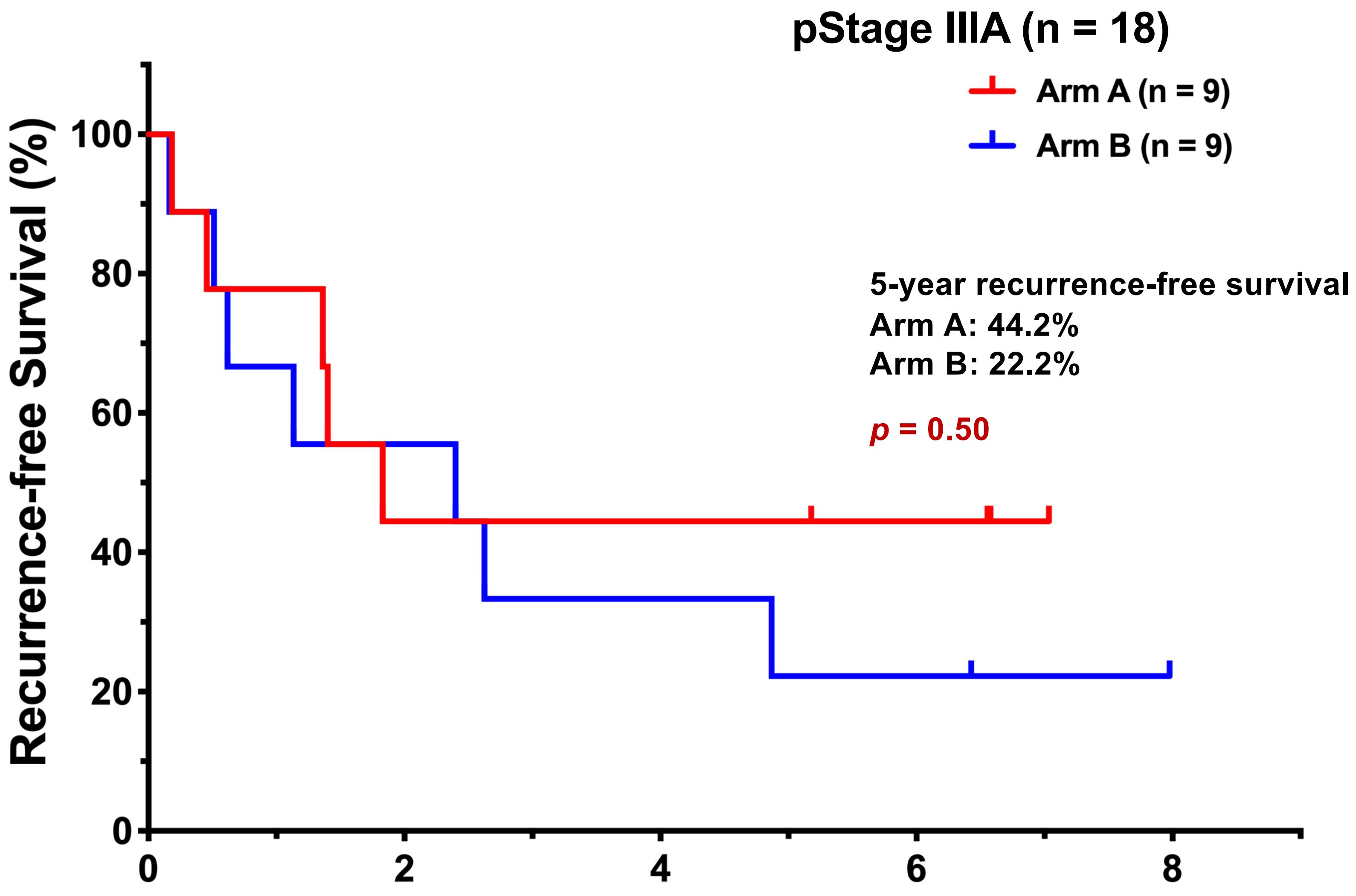

Number at risk					
Arm A	9	4	4	3	0
Arm B	9	5	3	2	0

S4 Fig J

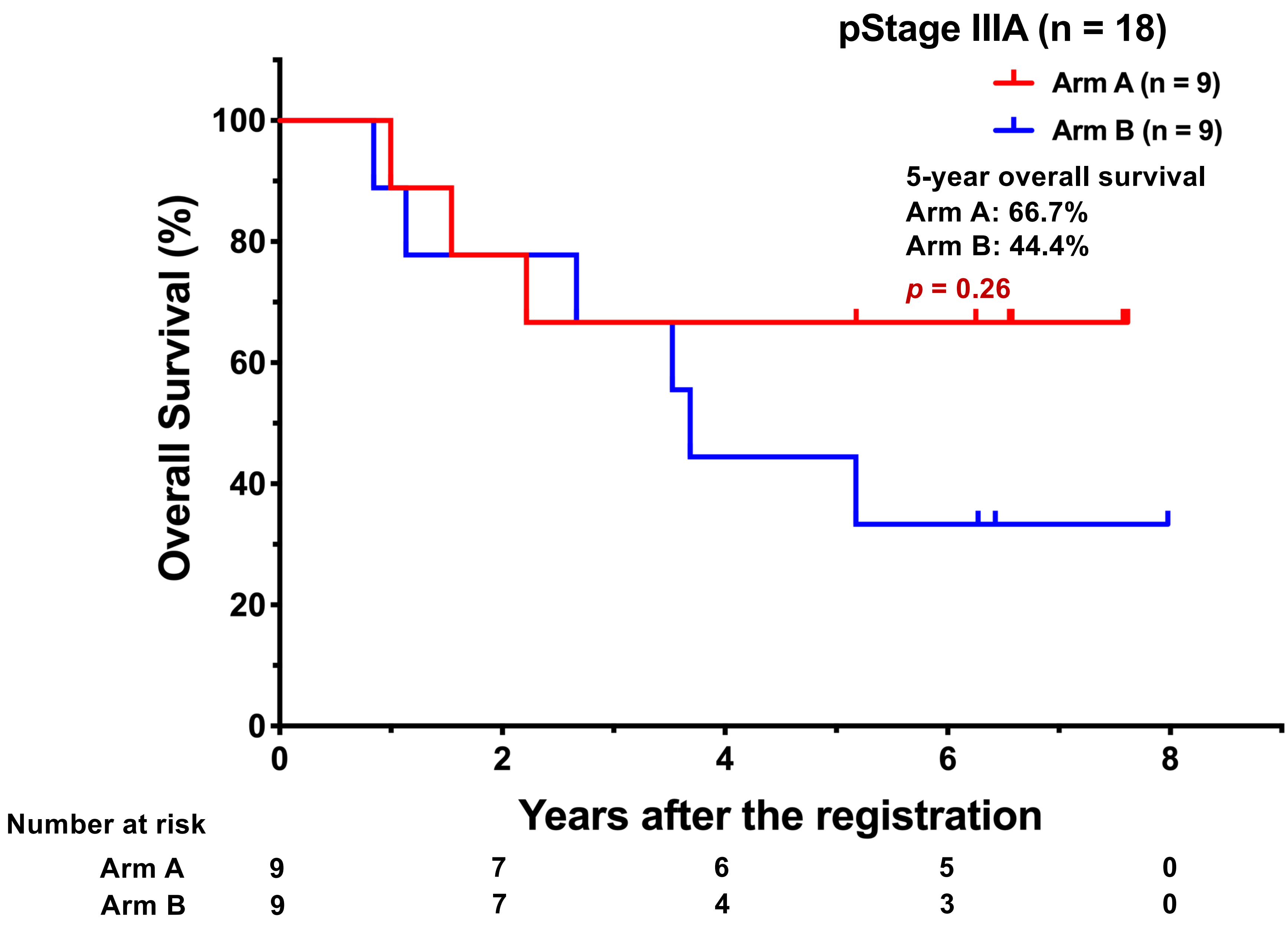
pStage IIIA (n = 18)
Arm A (n = 9)
Arm B (n = 9)
5-year overall survival
Arm A: 66.7%
Arm B: 44.4%
p = 0.26
Overall Survival (%)
100
80
60
40
20
0
0
2
4
6
8
Years after the registration
Number at risk
Arm A 9 7 6 5 0
Arm B 9 7 4 3 0
