# Supplementary material for: Randomized phase II study of daily versus alternate-day administrations of S-1 for the elderly patients with completely resected pathological stage IA (tumor diameter > 2 cm)—IIIA of non-small cell lung cancer: Setouchi Lung Cancer Group Study 1201
Source: PLoS One. 2023 May 19;18(5):e0285273. doi: 10.1371/journal.pone.0285273 (PMC10198543; doi:10.1371/journal.pone.0285273)
Supplement: S6 Fig — (A) Recurrence-free survival (RFS) of the patients by smoking status. (B) Overall survival (OS) of the patients by smoking status. (PDF) [file pone.0285273.s007.pdf]

S6 Fig A

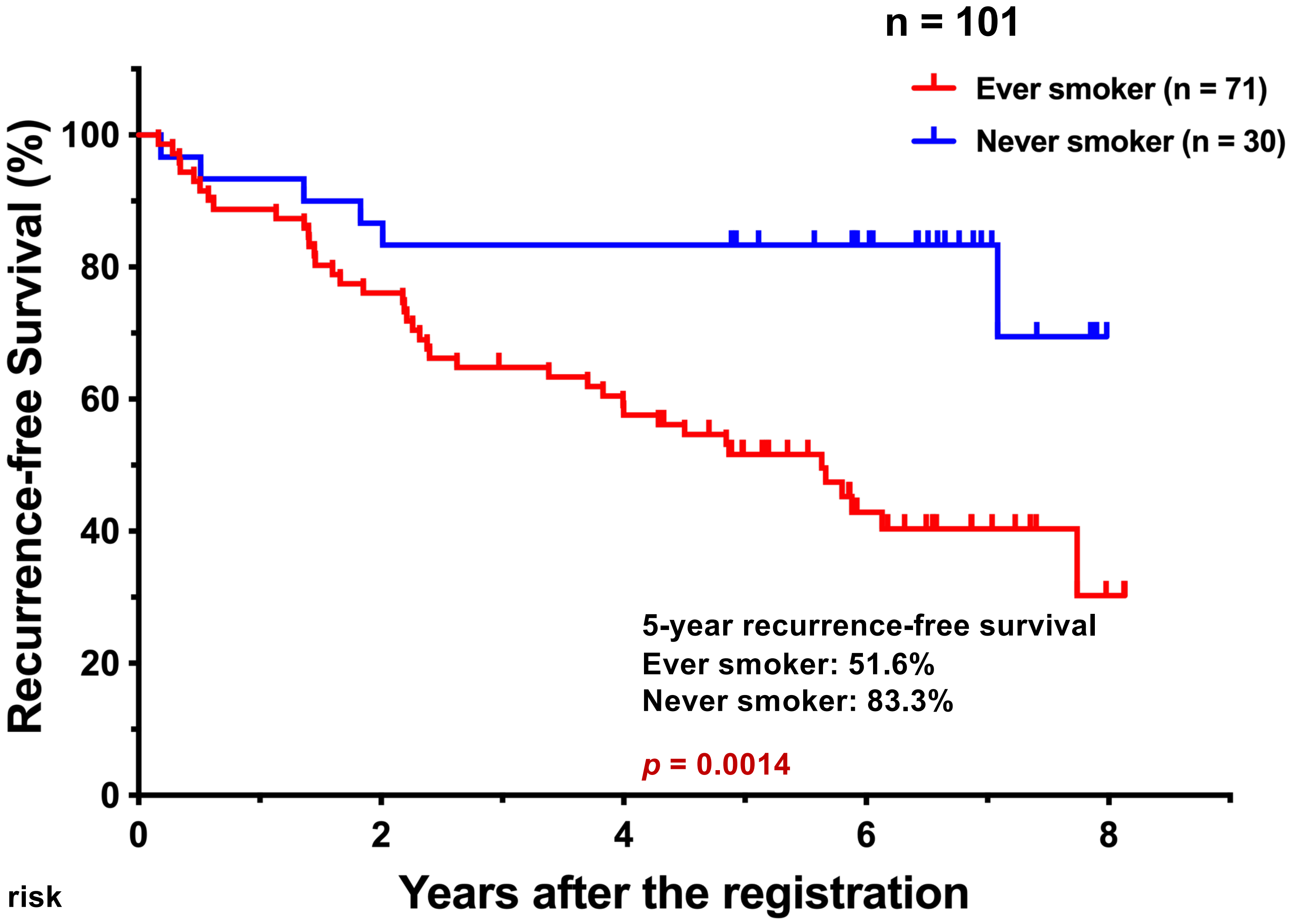

Number at risk

Ever smoker	71	54	41	17	2
Never smoker	30	26	25	17	0

S6 Fig B

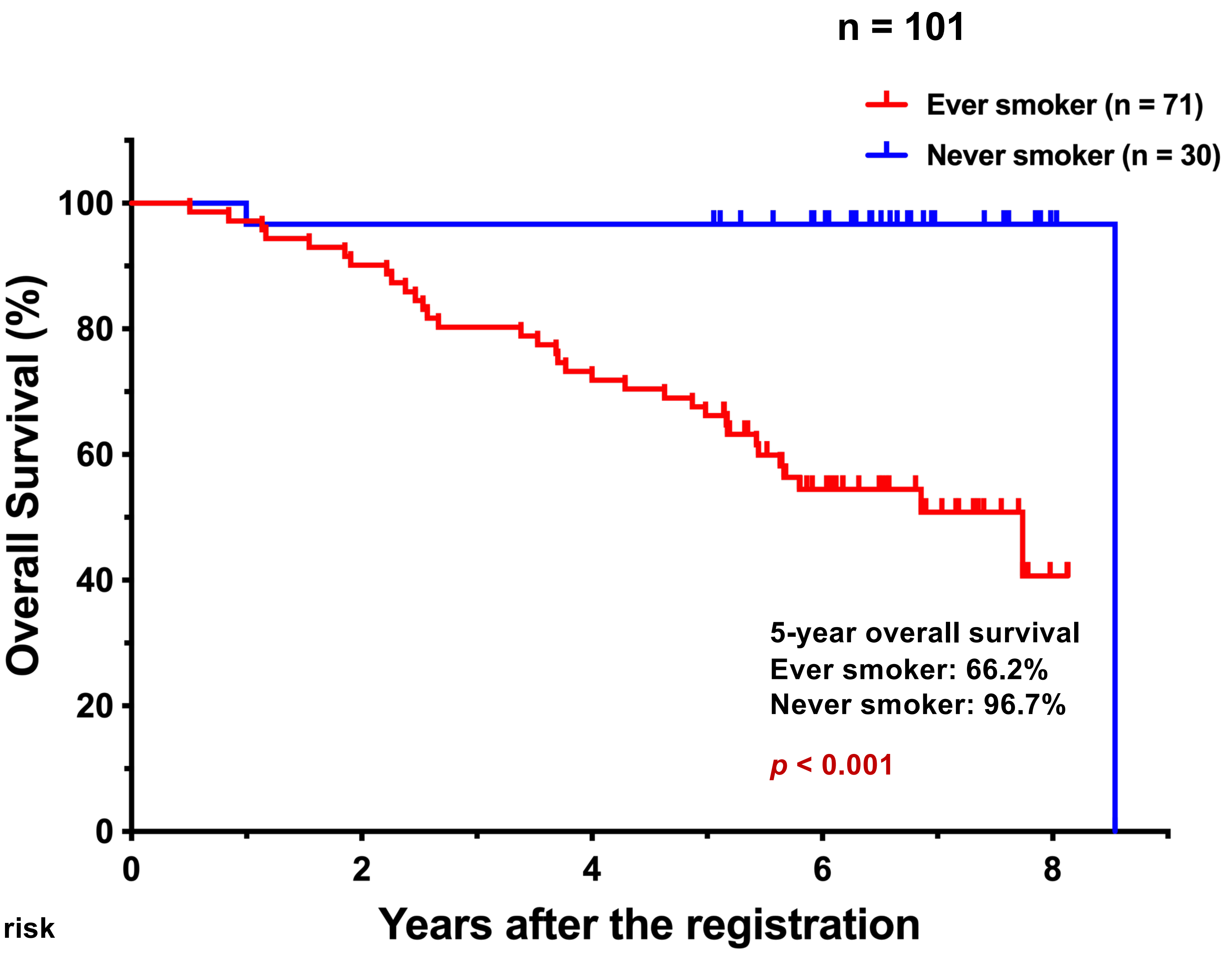

Number at risk

Ever smoker	71	64	52	26	2
Never smoker	30	29	29	23	2
