# Supplementary material for: Randomized phase II study of daily versus alternate-day administrations of S-1 for the elderly patients with completely resected pathological stage IA (tumor diameter > 2 cm)—IIIA of non-small cell lung cancer: Setouchi Lung Cancer Group Study 1201
Source: PLoS One. 2023 May 19;18(5):e0285273. doi: 10.1371/journal.pone.0285273 (PMC10198543; doi:10.1371/journal.pone.0285273)
Supplement: S1 File — (PDF) [file pone.0285273.s024.pdf]

Setouchi Lung Cancer Study Group
SLCG1201

Randomized phase II study of daily versus alternate-day administration of S-1 for the elderly patients with completely resected pathological stage IA (T1bN0M0)/IB/II/IIIA non-small cell lung cancer

Study protocol

Representative, Setouchi Lung Cancer Study Group
Shinichi Toyooka
Department of General Thoracic Surgery and Breast and Endocrinological Surgery, Okayama University Graduate School of Medicine, Dentistry and Pharmaceutical Sciences

Secretariat of SLCG1201
Ken Suzawa, Hiromasa Yamamoto, Mikio Okazaki
Department of Thoracic Surgery, Okayama University Hospital

Protocol author
Hiroshige Yoshioka[1], Katsuyuki Hotta[2], Shinichi Toyooka[3]
[1]Department of Thoracic Oncology, Kansai Medical University Hospital
[2]Center for Innovative Clinical Medicine and [3]Department of Thoracic Surgery, Okayama University Hospital

February 25, 2012: Concept approved by Executive Committee
March 15, 2012: Protocol version 1.0
July 10, 2012: Protocol version 1.1
January 4, 2013: Protocol version 1.2
March 4, 2014: Protocol version 1.3
May 24, 2017: Protocol version 1.4 (Addendum only due to revisions related to revised guidelines)
April 29, 2018: Protocol version 1.5
October 13, 2018: Protocol version 1.6
November 17, 2019: Protocol version 1.7
October 1, 2020: Protocol version 1.8

0. Overview

0.1. Summary of the study (Schema)

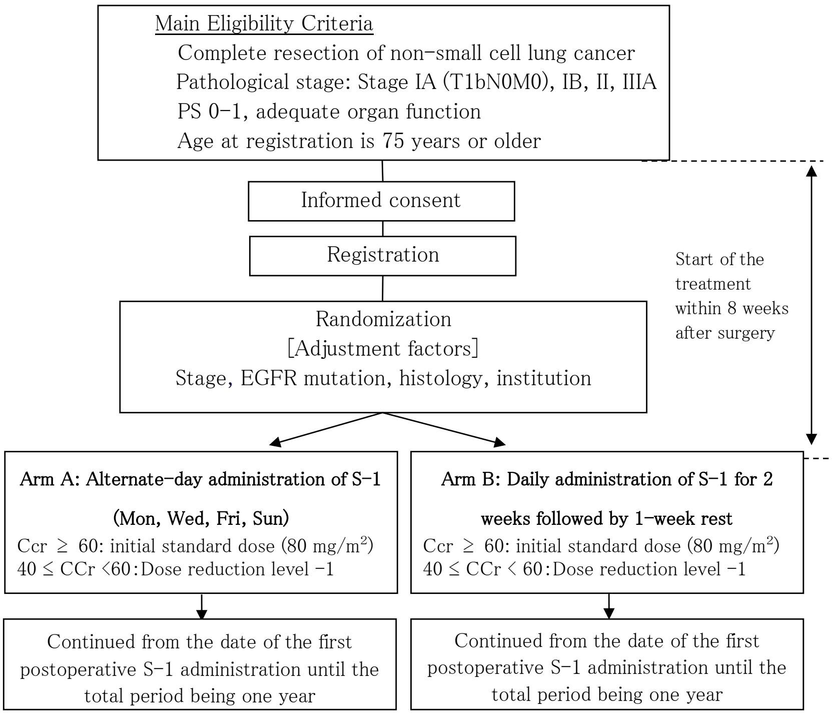

0.2. Purpose

To evaluate the tolerability and efficacy of alternate-day administration of S-1 as postoperative chemotherapy in elderly patients with completely resected pathological stage IA (T1bN0M0)/IB/II/IIIA non-small cell lung cancer.

Primary endpoint: Completion rate (Feasibility)

Secondary endpoints: rate and severity of adverse events, recurrence-free survival (RFS), overall survival (OS), quality of life (QOL)

0.3. Treatment

Arm A: alternate-day administration of S-1

S-1 is administered on Mondays, Wednesdays, Fridays, and Sundays and repeated until one of the criteria for discontinuation of treatment is met. Treatment with alternate-day administration of S-1 should be continued for 1 year counting from the date of the first S-1 administration.

If administration cannot be started more than 28 days after the last day of administration, this study treatment will be discontinued (day 1 will be the day after the last administration). The same day of the week 4 weeks after the last administration is acceptable).

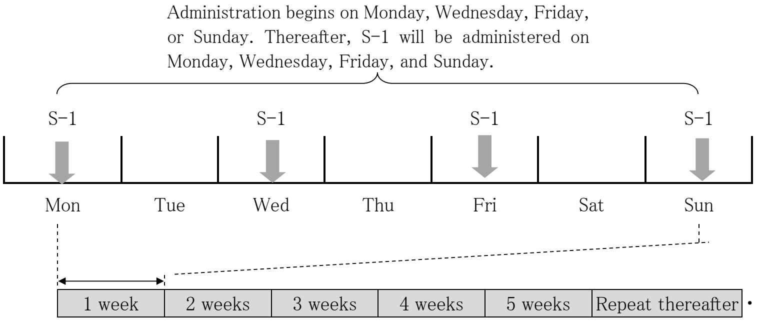

Arm B: Daily administration of S-1 for 2 weeks followed by 1-week rest

S-1 is taken orally every day for 14 days (after dinner on Day 1 to after breakfast on Day 15), followed by a 7-day rest period, repeated every 3 weeks as a course. This is repeated every 3 weeks for a total of 12 months from the start date of the protocol treatment. No new course will be started after the same calendar day 12 months after the start date of the protocol. The final course of treatment should be administered until the equivalent of day 14. If administration cannot be started more than 28 days after the last day of administration, this study treatment will be discontinued (day 1 will be the next day after the last administration). The same day of the week 4 weeks after the last administration is acceptable).

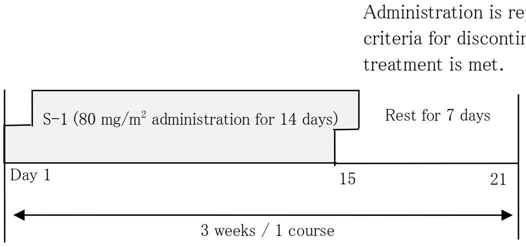

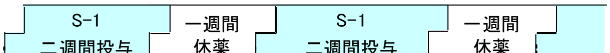

0.4. Subject

0.4.1. Target disease

Elderly patients with completely resected pathological stage IA (T1bN0M0)/IB/II/IIIA non-small cell lung cancer

0.4.2. Selection criteria

1) Pathological stage IA (T1bN0M0)/IB/II/IIIA cases with pathologically diagnosed non-small cell lung cancer. However, low-grade tumors such as carcinoid, mucoepidermoid carcinoma and adenoid cystic carcinoma are excluded.
2) Patients who have undergone surgical resection of lobectomy or more, lymph node dissection (ND2a or higher in principle), and pathologically confirmed complete resection.
3) Cases with no prior treatment other than surgery for the primary tumor.
4) Cases whose age is 75 years or older at the time of enrollment.
5) Cases with ECOG Performance Status (PS) of 0 to 1
6) No serious postoperative complications and laboratory values meet the following criteria
   - White blood cell count: 3,000-12,000/mm^3
   - Platelet count: 10 x 10^4/mm^3 or greater
   - Hemoglobin level: 9.0 g/dl or higher
   - Total bilirubin: 1.5 mg/dl or less
   - AST (GOT), ALT (GPT): less than 100 (IU/L)
   - PaO_2: 60 mmHg or higher (or SpO_2 90% or higher)
   - Creatinine: less than 1.2 mg/dl
   - Creatinine clearance (CCr) value*: 40 mL/min or more

   *For registration, creatinine clearance values measured by 24-hour urine storage or estimated by the Cockcroft-Gault formula will be used. However, the measured value is given priority. If the estimated value is 50 mL/min, additional measurement of the actual value is recommended.
7) Cases in which chemotherapy treatment is expected to start within 8 weeks after surgery
8) Patients whose consent to be a subject of this study has been explained to them in writing using a written explanation of consent and whose consent has been obtained in writing.

0.4.3. Exclusion criteria

Cases with any of the following items should be excluded

1) Cases with contraindications to S-1 administration
   - #1. Patients with a history of serious hypersensitivity to any of the components of S-1
   - #2. Patients with severe myelosuppression or renal or hepatic impairment
   - #3. Patients receiving other fluoropyrimidine antineoplastic agents
   - #4. Cases in which flucytosine is being administered
2) Cases with a history of serious drug allergy
3) Cases with a history of myocardial infarction that occurred within 6 months
4) Cases of interstitial pneumonia and pulmonary fibrosis evident on chest radiographs
5) Patients requiring regular oral administration of warfarin or dabigatran
6) Cases with clinically problematic changes on electrocardiogram (echocardiography, if necessary)
7) Cases with clinically problematic cardiac disease, psychiatric disease, infectious disease, or Cases with serious complications
8) Cases with difficult-to-control diabetes mellitus
9) Cases with intestinal paralysis and intestinal obstruction
10) Cases with watery diarrhea
11) Cases with active multiple cancers*
12) HBs antigen-positive cases
13) Other cases deemed inappropriate by the study investigator

*Active multiple cancers: Synchronous multiple cancers and metachronous multiple cancers within 5 years of disease-free interval. Carcinoma in situ (intraepithelial carcinoma) or lesion equivalent to intramucosal carcinoma that is considered curable by local treatment is not included in active multiple cancers.

0.5. Planned enrollment and study duration

Number of patients to be enrolled: 100

Registration period: 4 years (May 2012-April 2016)

Follow-up period for primary endpoint: 6 months after the end of enrollment

Research period: April 2012-June 2022

0.6. Inquiries

Principal Investigator (Setouchi Lung Cancer Study Group)

Shinichi Toyooka

Department of General Thoracic Surgery and Breast and Endocrinological Surgery, Okayama University Graduate School of Medicine, Dentistry and Pharmaceutical Sciences

Research Secretariat

Ken Suzawa, Hiromasa Yamamoto, Mikio Okazaki

Department of Thoracic Surgery, Okayama University Hospital, 2-5-1 Shikata-cho, Kita-ku, Okayama 700-8558, Japan

Tel: 086-235-7265, Fax: 086-235-7269

Table of contents

0. Overview 2
1. Purpose 8
2. Background 8
  2.1. Previous findings on adjuvant chemotherapy in Japan 8
  2.2. About S-1 8
  2.3. Alternate-day administration method for S-1 8
3. Criteria and definitions used in this study 10
  3.1. TNM tumor classification 10
  3.2. Staging (pathological stage) 11
  3.3. Definitions of terms related to treatment (postponement, withdrawal, suspension, discontinuation) 11
4. Patient selection criteria 12
  4.1. Selection criteria 12
  4.2. Exclusion criteria 12
5. Registration 13
  5.1. Registration procedure 13
  5.2. Precautions for registration 13
  5.3. Random assignment and adjustment factors 13
6. Protocol treatment 14
  6.1. Overview 14
  6.2. Arm A: S-1 alternate-day administration method 14
  6.3. Arm B: S-1 2-week administration followed by 1-week rest method 19
7. Protocol treatment discontinuation, termination criteria, and subsequent therapy 23
  7.1. Criteria for discontinuation of protocol treatment 23
  7.2. Follow-up for subjects who discontinued the study 23
  7.3. Definition of termination of protocol treatment 23
  7.4. Susequent therapy 23
8. Concomitant medications, and concomitant and supportive care 23
9. Anticipated adverse drug events 25
  9.1. Drug information 25
  9.2. Definition of adverse events 25
  9.3. Assessment of adverse events/adverse reactions 25
  9.4. Anticipated adverse reactions 25
  9.5. Causal relationship with study drug 25
10. Reporting adverse event 25
  10.1. Adverse events requiring urgent reporting 26
  10.2. Adverse events requiring normal reporting 26
  10.3. Reporting obligations and procedures of institutional principal investigators26
  10.4. Responsibilities of principal investigator/research secretariat 27
11. Observation/test items and timing 27
  11.1. Definition of treatment period 27
  11.2. Preoperative endpoints 27
  11.3. Pre-treatment (at enrollment) endpoints (within 2 weeks prior to enrollment) 27
  11.4. Endpoints during treatment period 27
  11.5. After discontinuation/termination of drug administration 28
  11.6. With signs of recurrence 28
12. Evaluation methods for assessment items 29
  12.1. Evaluation items 29
  12.2. Evaluation method 29
13. Target cases and study period 30
  13.1. Case accumulation period/follow-up period 30

13.2. Selection of subjects for analysis 30
13.3. Basis for setting the number of cases 31
13.4. Decision criteria for standard treatment (S-1 administration) based on the results 31
14. Discontinuation of the study 31
15. Data collection and storage 32
16. Ethical matters 32
16.1. Protection of subjects 32
16.2. Consent obtained 32
16.3. Explanation 32
16.4. Privacy protection and subject identification 33
16.5. Compliance with protocols 33
16.6. Approval of Institutional Review Board (IRB) or Ethics Review Board 33
16.7. Changes in protocol content 33
16.8. Changes in the contents of the case report form (CRF) 33
16.9. Secondary use of data 34
17. Monitoring and auditing 35
17.1. Monitoring items 35
17.2. Deviation from protocol 35
18. Costs and compensation 35
19. Research funding and conflicts of interest 35
20. Method of disclosing information on the study (registration of study plans and publication of study results) 35
21. Analysis for quality of life 36
21.1. Required samples 36
21.2. Method of investigation 36
21.3. Samples deliverly 36
21.4. Sample analysis 36
21.5. Post-analysis processing of samples 36
22. Disease reports 36
23. Research organization 37
23.1. Principal investigator (Setouchi Lung Cancer Study Group) 37
23.2. Research secretariat 37
23.3. Planned participating institutions 37
23.4. Data and Safety Monitoring Committee 37
23.5. Center for statistical analysis/registration 37
23.6. Data center 37
24. References 38

1. Purpose

To evaluate the tolerability and efficacy of alternate-day administration of S-1 as postoperative chemotherapy in elderly patients with completely resected pathological stage IA (T1bN0M0)/IB/II/IIIA non-small cell lung cancer.

Primary endpoint: Completion rate (Feasibility)

Secondary endpoints: rate and severity of adverse events, recurrence-free survival (RFS), overall survival (OS), quality of life (QOL)

2. Background

2.1. Previous findings on adjuvant chemotherapy in Japan

While surgical resection has traditionally been the first choice for stage I-IIIA non-small cell lung cancer, three recent large comparative trials[1)2)3)] conducted in the US and Europe have shown that platinum-based chemotherapy significantly improves survival rates. On the other hand, a meta-analysis of postoperative UFT monotherapy in Japan confirmed the efficacy of UFT[4)]. In response to this, since 2004, the addition of postoperative chemotherapy to surgery has been considered the standard of care for pathological stage IA (T1bN0M0) and IB-IIIA patients who have undergone complete resection by surgery. However, focusing on elderly patients, who often have other health problems, sufficient evidence has not been obtained to determine whether aggressive anticancer treatment is warranted. In other words, it is inconclusive whether postoperative chemotherapy for elderly patients can be expected to be more effective than the side effects caused by chemotherapy, and the development of treatment methods to improve treatment outcomes is needed.

2.2. About S-1

S-1 (tegafur/gimelacil/oteracil) is an oral fluoropyrimidine anticancer agent developed in Japan and approved in January 1999 as an effective treatment for gastric cancer. The combination of tegafur, a prodrug of 5-FU, with gimerasil (a reversible antagonist of the rate-limiting enzyme of 5-FU degradation) and potassium oterasil (a reversible inhibitor of phosphorylation of 5-FU to suppress gastrointestinal toxicity) increases blood 5-FU concentration and enhances anti-tumor effect. The drug is also designed to reduce the associated increase in gastrointestinal toxicity. [5) 6)]

The response rate of S-1 monotherapy (80 mg/m^2/day/equivalent) in untreated advanced non-small cell lung cancer in Japan was 22.0% after 4-week administration and 2-week rest period, which is much higher than the 6% response rate of UFT monotherapy, a fixed-dose combination of tegafur and uracil and comparable to conventional novel anticancer agents (taxanes and others).[7)] The main adverse events were gastrointestinal toxicity, including anorexia (10%) and diarrhea (9%).

In addition, a phase II study of single-agent S-1 (80 mg/m^2/day, 2-week administration followed by 1-week rest, for 1 year postoperatively) in patients with completely resected non-small cell lung cancer (Stage IB-IIIA) was conducted, and the 1-year DFS was 83.7% (Stage I: 95%, Stage II: 72.7%, Stage III: 75.0%) with promising results.[8)] In patients with resected head and neck cancer, a randomized comparison of the conventional adjuvant chemotherapy schedule of single-agent S-1 (80 mg/m^2/day/equivalent), 4-week administration followed by 2-week rest, with 2-week administration followed by 1-week rest, showed better compliance with the latter (6 months: 54% vs. 69%) and the incidence of diarrhea was also lower (10% vs. 28%).[9)] These results suggest that the latter method of administration is safer and more tolerable.

2.3. Alternate-day administration method for S-1

In the early 1960s, Lipkin[10)], Clarkson[11)], Cronkite[12)] et al. reported significant differences in the cell cycle between host normal cells (gastrointestinal mucosal cells, bone marrow cells) and cancer cells.

Shirasaka et al. first focused on normal host cells to reduce gastrointestinal and bone marrow toxicity of 5-FU, a highly time-dependent metabolic antagonist, and then biologically found a way to make it less cytotoxic to cancer cells.

The cell cycle of host normal cells is about half a day to one day, most of which is the S-phase in which 5-FU acts, which is about 12 hours, and a day (24 hours) of 5-FU non-exposure (drug withdrawal) renders a significant number of normal cells immune to 5-FU action. On the other hand, the cell cycle of cancer cells is longer than that of normal cells (4-5 days), and the S phase is longer than 24 hours, so the cell-killing effect of 5-FU on

cancer cells is not weakened by repeated exposure to 5-FU every 24 hours after a drug withdrawal. Shirasaka et al. also found that the cell-killing activity (IC_{50}) of 5-FU *in vitro* was not weakened by repeated on/off dosing every 6 or 12 hours and proposed a method of administering S-1 every other day.

The efficacy of the every other day administration method is becoming clear from basic and clinical results[13) 14)], and retrospective data from the Department of Gastroenterological Surgery, Jichi Medical University showed that 72.8% of 92 patients with recurrent or postoperative gastric cancer who started S-1 using the standard administration method of 4 weeks daily and 2 weeks rest had non-hematologic toxicity of Gr 1 or higher (nausea/vomiting; 16.3%, anorexia; 15.2%, diarrhea; 21.7%, general malaise; 19.6%). In 72 patients who had difficulty continuing the standard administration, S-1 was continued every other day after a 1-week break. As a result, non-hematologic toxicity of Gr 1 or higher was markedly reduced to anorexia (0%), nausea/vomiting (2.8%), in two patients and diarrhea (2.8%) in two patients, and the average duration of treatment was significantly extended to 272 days with every other day administration compared to 47 days with continuous administration. The TTP and MST were 170 days and 11 months, respectively, and the disease control rate among evaluable patients was 53% (31/58),[12)], which is a good result. The pharmacokinetics of the every other day administration regimen also showed that the Cmax blood concentration of 5-FU was comparable to that of the daily administration regimen.[13)]

Prospective clinical trials are currently reported in pancreatic cancer and gastric cancer.[14)15)] In pancreatic cancer, Yamagami et al. of Wakayama Medical University reported safety data from a phase II clinical trial of S-1 every other day in patients with unresectable advanced pancreatic cancer at the 49th Annual Meeting of Japan Society of Clinical Oncology. The results showed that gastrointestinal-related side effects were G1: 4.2%, G2: 6.2%, G3 or higher: 0%, and hematologic toxicity was G1: 6.2%, G2: 16.7%, G3: 4.2%, with a trend toward lower Grade 3 or higher side effects, and overall survival results after future follow-up are expected.[14)] In gastric cancer, Tsujitani et al. of the San-in Gastric Cancer Chemotherapy Study Group published the results of a phase II clinical trial at the ASCO GI in 2012, comparing the group of 4-week administration of S-1 followed by 2-week rest with the group of every other day administration, showing compliance (72.2% vs. 91.8%) and 1-year recurrence-free survival (82.9% vs. 91.7%). The every other day regimen is considered one of the treatment options for postoperative chemotherapy for gastric cancer.[15)] However, there is a lack of data from clinical trials for the use of every other day administration of S-1 in other carcinomas in clinical practice, thus data needs to be established as soon as possible.

Considering the above, the present study was designed to compare the durability of S-1 administered every other day (alternate-day administration on Monday, Wednesday, Friday and Sunday) with that of the aforementioned daily administration method (2-week administration followed by 1-week rest) in order to establish a better adjuvant chemotherapy regimen tailored to elderly patients.

3. Criteria and definitions used in this study

In this study, the definitions in the revised 7th edition of "General Rule for Clinical and Pathological Record of Lung Cancer" edited by the Japan Lung Cancer Society will be used.

3.1. TNM tumor classification

T-Primary tumor

T0 No evidence of primary tumor

Tis intraepithelial carcinoma in situ

T1: Tumor ≤ 3 cm in greatest dimension, covered by lung or visceral pleura, no central invasion beyond lobe bronchus by bronchoscopy (i.e., not extending into main bronchus)

T1a Tumor diameter ≤ 2 cm

T1b Tumor diameter > 2 cm and ≤ 3 cm

T2: Tumor > 3 cm and ≤ 7 cm in greatest dimension, or tumor ≤3 cm in greatest dimension but either of the following (T2a)

Extends into the main bronchus but ≥2 cm away from the tracheal bifurcation

Involvement of the pleura on the visceral side

Continuous atelectasis or obstructive pneumonia extending to the pulmonary hilum, but not involving the entire unilateral lung.

T2a Tumor > 3 cm in greatest diameter and ≤ 5 cm or ≤ 3 cm with pleural involvement (PL1, PL2, PL3 in interlobar cases)

T2b Tumor diameter > 5 cm and ≤ 7 cm

T3: Tumor > 7 cm in greatest dimension; direct involvement of chest wall (including superior sulcus tumor), diaphragm, phrenic nerve, mediastinal pleura, or pericardium; extension into main bronchus less than 2 cm from bifurcation but not into bifurcation; atelectasis or obstructive pneumonia involving one lung; discontinuous collateral tumor nodules within the same lobe

T4: Involvement of mediastinum, heart, great vessels, trachea, recurrent nerve, esophagus, vertebral body, tracheal bifurcation, or paratumor nodules in different lung lobes on the same side

N-affiliated lymph node

N0 No lymph node metastasis

N1 Metastases in ipsilateral peribronchial and/or ipsilateral hilar lymph nodes and intrapulmonary lymph nodes, including involvement by direct extension

N2 Metastasis in ipsilateral mediastinal or subcarinal lymph nodes

N3 Metastasis in contralateral mediastinal, contralateral hilar, ipsilateral or contralateral anterior scalene muscle, supraclavicular lymph nodes

M-Distant metastasis

M0 No distant metastasis

M1 Distant metastasis.

M1a Paraneoplastic nodule in contralateral lung, pleural nodule, malignant pleural effusion (ipsilateral, contralateral), malignant pericardial effusion

M1b Distant metastasis to other organs

M1 should be listed as follows depending on the metastatic organ

Pulmonary PUL, bone marrow MAR, bone OSS, pleural PLE, hepatic HEP, peritoneal PER

Brain BRA, adrenal ADR, lymph node LYM, skin SKI, other OTH

pTNM Classification and pathological classification

The pT, pN, and pM classifications are the same as the T, N, and M classifications.

To determine pN0, at least six hilar and mediastinal lymphadenectomy specimens should usually be histologically retrieved.

3.2. Staging (pathological stage)

Stage	T	N	M
0	carcinoma in situ	N0	M0
IA	T1a or T1b	N0	M0
IB	T2a	N0	M0
IIA	T1a or T1b	N1	M0
	T2a	N1	M0
	T2b	N0	M0
IIB	T2b	N1	M0
	T3	N0	M0
IIIA	T1a or T1b	N2	M0
	T2a	N2	M0
	T2b	N2	M0
	T3	N2	M0
	T3	N1	M0
	T4	N0	M0
	T4	N1	M0
IIIB	Any T	N3	M0
	T4	N2	M0
IV	Any T	Any N	M1a or M1b

3.3. Definitions of terms related to treatment (postponement, withdrawal, suspension, discontinuation)

Postponement: Delaying the administration of S-1 beyond the prescribed time.

Withdrawal: Cessation of drug administration as per the schedule specified in the protocol.

Suspension: Suspension of drug administration outside the schedule due to the occurrence of adverse events or for other reasons, with the possibility of resumption of administration.

Discontinuation: A termination of protocol treatment with no possibility of resumption.

4. Patient selection criteria

4.1. Selection criteria

1) Pathological stage IA (T1bN0M0)/IB/II/IIIA cases with pathologically diagnosed non-small cell lung cancer. However, low-grade tumors such as carcinoid, mucoepidermoid carcinoma and adenoid cystic carcinoma are excluded.
2) Patients who have undergone surgical resection of lobectomy or more, lymph node dissection (ND2a or higher in principle), and pathologically confirmed complete resection.
3) Cases with no prior treatment other than surgery for the primary tumor.
4) Cases whose age is 75 years or older at the time of enrollment.
5) Cases with ECOG Performance Status (PS) of 0 to 1
6) No serious postoperative complications and laboratory values meet the following criteria
   White blood cell count: 3,000-12,000/mm^3
   Platelet count: 10 x $10^4/mm^3$ or greater
   Hemoglobin level: 9.0 g/dl or higher
   Total bilirubin: 1.5 mg/dl or less
   AST (GOT), ALT (GPT): less than 100 (IU/L)
   PaO_2: 60 mmHg or higher (or SpO_2 90% or higher)
   Creatinine: less than 1.2 mg/dl
   Creatinine clearance (CCr) value*: 40 mL/min or more
   *For registration, creatinine clearance values measured by 24-hour urine storage or estimated by the Cockcroft-Gault formula will be used. However, the measured value is given priority. If the estimated value is 50 mL/min, additional measurement of the actual value is recommended.
7) Cases in which chemotherapy treatment is expected to start within 8 weeks after surgery
8) Patients whose consent to be a subject of this study has been explained to them in writing using a written explanation of consent and whose consent has been obtained in writing.

4.2. Exclusion criteria

Cases with any of the following items should be excluded

1) Cases with contraindications to S-1 administration
   #1. Patients with a history of serious hypersensitivity to any of the components of S-1
   #2. Patients with severe myelosuppression or renal or hepatic impairment
   #3. Patients receiving other fluoropyrimidine antineoplastic agents
   #4. Cases in which flucytosine is being administered
2) Cases with a history of serious drug allergy
3) Cases with a history of myocardial infarction that occurred within 6 months
4) Cases of interstitial pneumonia and pulmonary fibrosis evident on chest radiographs
5) Patients requiring regular oral administration of warfarin or dabigatran
6) Cases with clinically problematic changes on electrocardiogram (echocardiography, if necessary)
7) Cases with clinically problematic cardiac disease, psychiatric disease, infectious disease, or Cases with serious complications
8) Cases with difficult-to-control diabetes mellitus
9) Cases with intestinal paralysis and intestinal obstruction
10) Cases with watery diarrhea
11) Cases with active multiple cancers*
12) HBs antigen-positive cases
13) Other cases deemed inappropriate by the study investigator

*Active multiple cancers: Synchronous multiple cancers and metachronous multiple cancers within 5 years of disease-free interval. Carcinoma in situ (intraepithelial carcinoma) or lesion equivalent to intramucosal carcinoma that is considered curable by local treatment is not included in active multiple cancers.

5. Registration

5.1. Registration procedure

The principal investigator/participating investigator confirms that the eligible patients meet all the eligibility criteria and do not violate the exclusion criteria, fills out the "Case Registration Form", and faxes the form to the registry center. After the registration center confirms eligibility, it will fax back a notification of case registration results to the physician in charge, indicating the case registration number. The procedure should be started within 8 weeks after the surgery according to the instructions in the registration confirmation letter.

(Registration Office)

Registration Office: Department of Thoracic Surgery, Okayama University Hospital
Kazuhiko Shien, Hiromasa Yamamoto, Junichi Soh
Fax: 086-235-7269 (direct number for Department of Thoracic Surgery)

5.2. Precautions for registration

(1) Post-registration after protocol treatment has been initiated is not acceptable.
(2) When the case registration form is not complete, registration will not be accepted until all the information is fulfilled.
(3) A case registration number is issued after eligibility is verified by the registration center.
(4) Once registered, the registration center will fax back a notification of case registration results with the case registration number to the physician in charge, who should keep it in an appropriate place.
(5) Once a case is registered, it will not be cancelled (deleted from the database). In case of duplicate registration, the first registration information (case registration number) shall be used.
(6) When an incorrect or duplicate registration is found, the registration center should be notified immediately.
(7) As a rule, registration must be made by faxing the case registration form at least one day prior to the scheduled start of treatment.

5.3. Random assignment and adjustment factors

Upon enrollment, each patient will be randomly assigned to a treatment by Dr. Keitaro Matsuo, Department of Preventive Medicine, Kyushu University Faculty of Medical Sciences. Random assignment will be performed using a minimization method with four factors as assignment adjustment factors: pathologic stage (IA(T1bN0M0)/stage IB or stage II or stage IIIA), histologic type (non-squamous or squamous cell carcinoma (if difficult to determine, non-squamous should be used)), EGFR mutation (mutated, unmutated or unknown), and institution.

6. Protocol treatment

6.1. Overview

The alternate-day administration of S-1 and 2-week administration of S-1 followed by 1-week rest are defined as protocol treatment in this study. As a rule, protocol treatment will be initiated within 1 week after enrollment and within 8 weeks after surgery. If more than 8 weeks have elapsed since surgery, the data center will be contacted to confirm whether protocol treatment can be started, and if so, it will be initiated. After initiation, the reason why the treatment could not be started within 8 weeks should be noted in the comment section of the "Report of Study Initiation and Pre-Treatment Discontinuation". TS-1 should be TS-1 combination capsules T20, T25, TS-1 combination granules T20, T25, and TS-1 combination OD tablets T20, T25 (Taiho Pharmaceutical Co).

6.2. Arm A: S-1 alternate-day administration method

6.2.1. Treatment schedule for Arm A (S-1 alternate-day administration method)

Treatment should begin within 8 weeks after surgery.

<u>Administration Schedule</u>

The first dose of S-1 should be initiated on Monday, Wednesday, Friday, or Sunday (the specified day of the week); Day 1 is defined as the day on which S-1 is initiated.

If S-1 cannot be administered after breakfast, it may be started after dinner. Even if S-1 is started after dinner, the day should be defined as the day on which S-1 is initiated, and the next dose should be taken on the specified day of the week.

(e.g., if taken on Friday evening, the next dose will be given on Sunday morning)

S-1 should be administered on Mondays, Wednesdays, Fridays, and Sundays and should be continued until one of the criteria for discontinuation of treatment is met. **<u>S-1 should not be administered other than the specified day.</u>**

- If administration cannot be started more than 28 days after the last day of administration, this study treatment will be discontinued (day 1 will be the day after the last dose). The same day of the week 4 weeks after the last dose is acceptable).

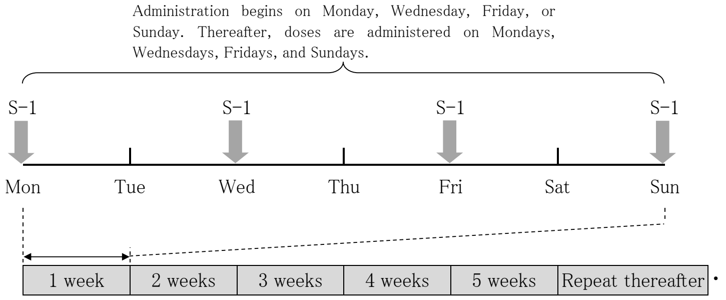

<u>Starting dose and starting method</u>

The daily dose of S-1 is calculated according to body surface area from Table 1. "S-1 Initial standard dose and one-step dose reduction" and divided equally into two portions and administered orally after breakfast and dinner. If the creatinine clearance (CCr) at the time of enrollment is between 40 mL/min and 60 mL/min, S-1 should be started at a dose reduced by one step from the initial standard dose according to Table 1. The baseline weight is defined as the weight in which initial dose was calculated before chemotherapy. If the weight is reduced by more than 10% from the baseline, recalculate the baseline weight and use the weight at the time of recalculation as the baseline weight thereafter.

Table 1. S-1 initial standard dose and one-step dose reduction

Body surface area	Initial standard dose (FT equivalent) (CCr ≥ 60)	Dose reduction level -1 (40 ≤ CCr < 60)
< 1.25 m^2	80 mg/day	50 mg/day
≥ 1.25 m^2 and < 1.5 m^2	100 mg/day	80 mg /day
≥ 1.5 m^2	120 mg/day	100 mg /day

6.2.2. Criteria for starting administration (Arm A)

At the start of the first administration, confirm that 4.1. selection criteria 6) are met on the day before or on the day of the start of administration and start administration.

If the criteria are not met, the start of administration should be postponed, and the first administration should be started after confirming that the relevant items meet the 4.1. selection criteria 6).

6.2.3. Criteria for continuing administration (Arm A)

After the first administration, S-1 administration will be suspended if the "Dose Continuation Criteria" in Table 2 are not met.

When it is confirmed that the "Criteria for continuing administration" in Table 2 are not met within 21 days of the first administration, S-1 should be suspended, and a reduced dose of S-1 should be considered from the next dose according to Table 5. Dose reduction levels.

Table 2. Criteria for continuing administration

Item	Criteria for continuing administration
White blood cell count	≥ 2,000 /mm^3 and ≤ 12,000 /mm^3
Neutrophil count	≥ 1,000 /mm^3
Platelet count	≥ 75,000 /mm^3
Total bilirubin	≤ 1.5 mg/dL
AST and ALT	≤ 100 IU/L
Creatinine	< 1.2 mg/dL
Pneumonia	Grade 0
Infection	No fever of suspected infection
PS	0 to 1
Diarrhea, mouth ulcers	Grade 1 or below
Other non-hematologic toxicity	Grade 2 or below (However, the physician in charge may decide in his/her discretion to allow administration.)
Others	Drug withdrawal is allowed when deemed necessary by the physician in charge due to the occurrence of adverse events that do not fall under the above categories.

6.2.4. Criteria for resumption of administration (Arm A)

When restarting the drug after suspension, make sure that the "Criteria for resumption of administration" in Table 3 are met. However, if the adverse event meets the "Dose Reduction Criteria" in Table 4, the dose should be reduced from the time of resumption. If the drug is suspended due to an adverse event other than those listed in Table 2 "Criteria for continuing administration", the grade of the adverse event that caused the suspension must have improved by at least one level, and the physician in charge must determine that the drug can be administered.

If S-1 treatment cannot be resumed after 28 days, protocol treatment should be discontinued.

Table 3. Criteria for resumption of administration

Item	Criteria for resumption of administration
White blood cell count	≥ 3,000/mm³ and ≤12,000 /mm³
Neutrophil count	≥ 1,500/mm³
Platelet count	≥ 100,000 /mm³
Total bilirubin	≤ 1.5 mg/dL
AST and ALT	≤ 100 IU/L
Creatinine	< 1.2 mg/dL
Pneumonia	Grade 0
Infection	No fever of suspected infection
PS	0 to 1
Diarrhea, mouth ulcers	Grade 1 or below
Other findings and general clinical findings	Grade 2 or below (However, the physician in charge may decide in his/her discretion to allow administration.)

6.2.5. Criteria for dose reduction (Arm A)

When resuming administration in patients with adverse events that meet the dose reduction criteria specified in Table 4, the dose should be reduced one step at a time according to "Dose reduction levels" in Table 5. However, the minimum dose of S-1 is 50 mg/day, and the dose will not be increased again after a dose reduction. For adverse events other than those listed in Table 4, the S-1 dose may be reduced at the discretion of the treating physician, again in accordance with the dose reduction levels listed in Table 5.

Table 4. Criteria for dose reduction

Item	Criteria for dose reduction
White blood cell count	< 1,000 /mm³ (Grade 4)
Neutrophil count	< 500 /mm³ (Grade 4)
	Febrile neutropenia (Grade 3 or higher)
Platelet count	< 25,000 /mm³ (Grade 4)
	Grade 3 thrombocytopenia with platelet transfusion
Creatinine	≥ 1.2 mg/dL
Total bilirubin	≥ 2.0 mg/dL
Non-hematologic toxicity (except low Na)	Grade 3 and above

Grade 4 non-hematologic toxicities (Grade 1 or higher for pulmonary inflammation) will result in discontinuation of protocol treatment.

Table 5. Dose reduction levels (dose reduction methods for S-1)

Body surface area	At the start of treatment (FT equivalent)	Dose reduction level			
		-1	-2	-3	-4
< 1.25 m²	80 mg/day	50 mg/day	Cessation of treatment	-	-
≥ 1.25 m² and < 1.5 m²	100 mg/day	80 mg/day	50 mg/day	Cessation of treatment	-
≥ 1.5m²	120 mg/day	100 mg/day	80mg/day	50 mg/day	Cessation of treatment

6.2.6. Criteria for dose escalation (Arm A)

After 43 days of treatment, if no adverse events are observed that would result in withdrawal/dose reduction and

the physician in charge determines that there are no safety issues, the dose of S-1 may be increased by one step only from the initial standard dose in accordance with "Dose escalation levels" in Table 6.

The dose should not be increased in patients whose dose has been reduced since the first dose due to the CCr value at the time of registration (40 ≤ CCr < 60).

The dose should not be increased again in cases where the dose has been reduced.

Dose reductions for increased doses follow the dose reduction levels in Table 7.

Table 6. Dose escalation levels

The method for the dose escalation of S-1		
Body surface area	Initial standard dose (FT equivalent)	Dose escalation level
		+1
< 1.25 m^2	80 mg/day	100 mg/day
≥ 1.25 m^2 and < 1.5 m^2	100 mg/day	120 mg/day
≥ 1.5m^2	120 mg/day	150 mg/day

Table 7. Dose reduction levels for the cases with dose escalation

The method for the dose reduction for the cases with dose escalation of S-1				
Body surface area	Dose after increase (FT equivalent)	Dose reduction level		
		-1	-2	-3
< 1.25 m^2	100 mg/day	80 mg/day	50 mg /day	Cessation of treatment
≥ 1.25 m^2 and < 1.5 m^2	120 mg/day	100 mg/day	80 mg/day	50 mg/day
≥ 1.5m^2	150 mg/day	120 mg/day	100 mg/day	80 mg/day

6.2.7. Criteria for completion of protocol treatment (Arm A)

Protocol treatment is terminated when one year has elapsed from the date of the first administration of S-1 as adjuvant chemotherapy after surgery for lung cancer.

6.2.8. Administration Simulation (Arm A)

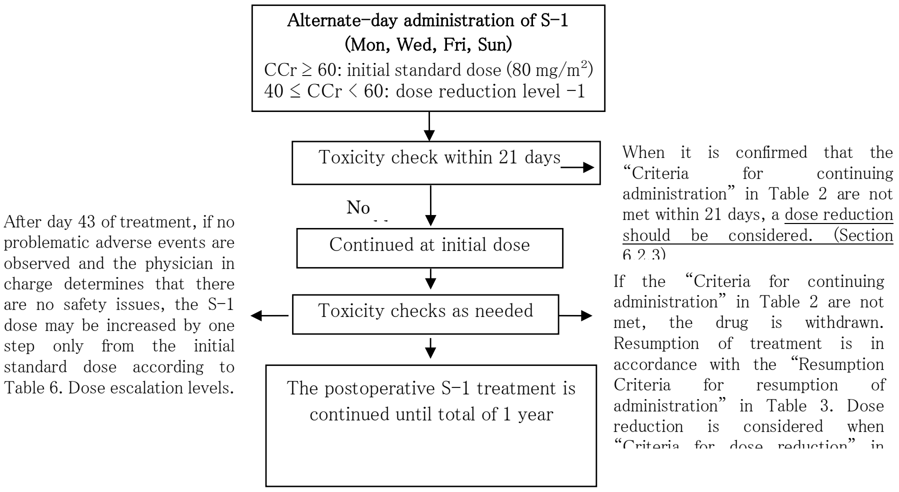

6.3. Arm B: S-1 2-week administration followed by 1-week rest method

6.3.1. Treatment schedule for Arm B (S-1 2-week administration followed by 1-week rest method)

Administration cycle: Oral administration every day for 14 consecutive days from Day 1 (after dinner on Day 1 to after breakfast on Day 15)

The drug is then withdrawn for 7 days, and this is repeated every 3 weeks for one course.

End date of treatment: Administration should be performed until the total treatment period from the protocol treatment start date has reached 12 months. No new courses should be started after the same calendar day 12 months after the protocol treatment start date. The final course of treatment should be administered until a date equivalent to day 14.

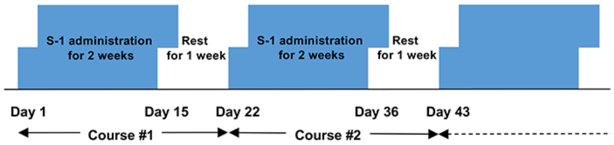

Starting dose and starting method

The daily dose of S-1 is calculated according to body surface area from Table 8. “S-1 initial standard dose and one-step dose reduction” and divided equally into two portions and administered orally after breakfast and dinner. If the creatinine clearance (CCr) at the time of enrollment is between 40 mL/min and 60 mL/min, S-1 should be started at a dose reduced by one step from the initial standard dose according to Table 8. If the patient's body weight has decreased by more than 10% from the baseline, the dose is recalculated and the baseline body weight is considered to be the weight at the time of recalculation.

Table 8. S-1 initial standard dose and one-step dose reduction

Body surface area	Initial standard dose (FT equivalent) (CCr ≥ 60)	Dose reduction level -1 (40 ≤ CCr < 60)
< 1.25m^2	80 mg/day	50 mg/day
≥ 1.25 m^2 and < 1.5 m^2	100 mg/day	80 mg/day
≥ 1.5m^2	120 mg/day	100 mg/day

6.3.2. Course start criteria for Arm B (S-1 2-week administration followed by 1-week rest)

The start of the next course is decided after confirming that all of the criteria in Table 9 below are met on the day before or the day of the scheduled start of the course. If even one of the criteria is not met, administration is postponed, and administration is started as soon as the patient recovers.

However, for the start of the second and subsequent courses, **if these criteria are not met 21 days beyond the scheduled start of the next course, the study of the case will be terminated** (see figure below).

In the case of a postponement of the start of a course, the start date after the postponement shall be Day 1 of the course.

If recurrence is observed during the course of treatment, protocol treatment should be discontinued at that time.

Table 9: Course Start Criteria (Arm B)

Item		Criteria for starting the course
Blood toxicity	White blood cell count	≥ 3,000/mm^3
	Neutrophil count	≥ 1,500/mm^3
	Platelet count	≥ 100,000/mm^3
Non-hematological	Total bilirubin	≤ 1.5 mg/dL

toxicity	AST and ALT	≦ 100 IU/L
	Creatinine	< 1.2 mg/dL
	Pneumonia	Grade 0
	Infection	No fever with infection
	PS	0 to 1
	Other subjective and general clinical findings	Grade 2 or below (but may be administered at the discretion of the physician in charge)

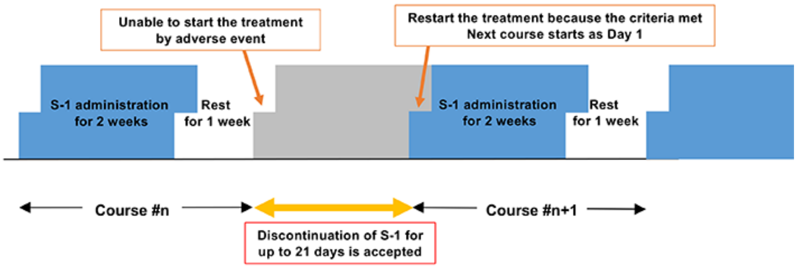

6.3.3. Criteria for in-course withdrawal and resumption (Arm B)

If an adverse event that meets the criteria in Table 10 develops during S-1 administration, S-1 will be withdrawn. If the adverse event has not resolved by the end of breakfast on Day 15, no further S-1 should be administered for that course. If the patient has not recovered from a Grade 4 non-hematologic toxicity (Grade 1 or higher for pulmonary inflammation), protocol treatment should be discontinued.

Table 10. Criteria for decision of withdrawal of S-1 monotherapy

Item		Criteria for withdrawal	Criteria for resumption
Blood toxicity	White blood cell count	< 2,000/mm ^{3}	≥ 2,000/mm^3
	Neutrophil count	<1,000/mm ^{3}	≥ 1,000/mm^3
	Platelet count	< 75,000/mm ^{3}	≥ 75,000/mm^3
Non-hematological toxicity	Total bilirubin	> 1.5 mg/dL	≦ 1.5 mg/dL
	AST and ALT	Greater than 100 IU/L	100 IU/L or less
	Creatinine	≥ 1.2 mg/dL	< 1.2 mg/dL
	Infection	Fever of suspected infection	No fever of suspected infection
	PS	2 or more	0 to 1
	Diarrhea, mouth ulcers	Grade 2 or above	Grade 1 or below
	Other non-hematologic toxicity	Grade 3 or above	Grade 2 or below (However, the physician in charge may decide in his/her discretion to allow administration.)
	Others	Drug withdrawal is allowed when deemed necessary by the physician in charge due to the occurrence of adverse events that do not fall under the above categories.	Adverse event that caused withdrawal from the drug is in remission

Grade 4 non-hematologic toxicities (Grade 1 or higher for pulmonary inflammation) will result in discontinuation of protocol treatment.

6.3.4. Criteria for dose reduction (Arm B)

When resuming dosing in patients with adverse events that meet the criteria for dose reduction specified in Table 11, the dose should be reduced one step at a time according to Table 12, “Dose reduction levels”. However, the minimum dose of S-1 is 50 mg/day, and the dose will not be increased again after a dose reduction. For adverse events other than those listed in Table 11, the S-1 dose may be reduced at the discretion of the physician in charge, again in accordance with the dose reduction levels listed in Table 12.

Table 11. Criteria for dose reduction

Item	Criteria for dose reduction
White blood cell count	< 1,000 /mm³ (Grade 4)
Neutrophil count	< 500 /mm³ (Grade 4)
	Febrile neutropenia (Grade 3 or higher)
Platelet count	< 25,000 /mm³ (Grade 4)
	Grade 3 thrombocytopenia with platelet transfusion
Creatinine	≥ 1.2 mg/dL
Total bilirubin	≥ 2.0 mg/dL
Non-hematologic toxicity (except low Na)	Grade 3 or above

Grade 4 non-hematologic toxicities (Grade 1 or higher for pulmonary inflammation) will result in discontinuation of protocol treatment.

Table 12. Dose reduction levels (dose reduction methods for S-1)

Body surface area	At the start of treatment (FT equivalent)	descent level			
		-1	-2	-3	-4
< 1.25 m²	80 mg/day	50 mg/day	Cessation of treatment	-	-
≥ 1.25 m² and <1.5 m²	100 mg/day	80 mg/day	50 mg/day	Cessation of treatment	-
≥ 1.5 m²	120 mg/day	100 mg/day	80mg/day	50 mg/day	Cessation of treatment

6.3.3. Dose escalation (Arm B)

After the second course, if there are no adverse events that would result in withdrawal/reduction of the dose and the physician in charge determines that there are no safety issues, the dose of S-1 may be increased by one step only from the initial standard dose according to Table 13. Dose escalation levels.

The dose should not be increased in patients whose dose has been reduced since the first dose due to the CCr value at the time of registration (40 ≤ CCr < 60).

The dose should not be increased again in cases where the dose has been reduced.

Dose reductions for increased doses follow the dose reduction levels in Table 14.

Table 13. Dose escalation levels

The method for the dose escalation of S-1		
Body surface area	Initial standard dose (FT equivalent)	Dose escalation level
		+1
< 1.25 m²	80 mg/day	100 mg/day
≥ 1.25 m² and <1.5 m²	100 mg/day	120 mg/day
≥ 1.5 m²	120 mg/day	150 mg/day

Table 14. Dose reduction levels for the cases with dose escalation

The method for the dose reduction for the cases with dose escalation of S-1				
Body surface area	Dose after increase (FT equivalent)	Dose reduction level		
		−1	−2	−3
< 1.25 m^2	100 mg/day	80 mg/day	50 mg/day	Cessation of treatment
≥ 1.25 m^2 and <1.5 m^2	120 mg/day	100 mg/day	80 mg/day	50 mg/day
≥ 1.5 m^2	150 mg/day	120 mg/day	100 mg/day	80 mg/day

7. Protocol treatment discontinuation, termination criteria, and subsequent therapy

7.1. Criteria for discontinuation of protocol treatment

If any of the following criteria are met, the physician in charge will discontinue protocol treatment

1) If protocol treatment cannot be started 28 days after the last day of S-1 treatment (which corresponds to 21 days after the scheduled start of the next course in Arm B) (in this case, the day after the last day of S-1 treatment is considered Day 1). Starting administration on the same day of the week is acceptable).
2) If a withdrawal of more than 28 days is required (in this case, the day after the last day of S-1 administration shall be considered as Day 1. Resumption on the same day of the week 4 weeks after the date of the last dose is acceptable)
3) When an adverse event that meets each of the dose reduction criteria occurs even if the S-1 dose is reduced to the lowest level
   Or if a Grade 4 non-hematologic toxicity (Grade 1 or higher for pulmonary inflammation) develops.
4) When an adverse event occurs that makes it difficult to continue administration
5) If the physician in charge determines that the disease has recurred
6) If the patient requests discontinuation
7) When matters that should be excluded from the subjects turned out after registration
8) Other cases in which the physician in charge deems it necessary to discontinue the study.

7.2. Follow-up for subjects who discontinued the study

If any of the treatment discontinuation criteria are met, protocol treatment should be discontinued, and the timing, reason, and progress should be recorded in the "case report form" and evaluated up to the point of discontinuation. In addition, follow-up of adverse events should be conducted in discontinued cases until 4 weeks (day 28) after the last administration of S-1, or until the adverse events that may have been caused by the drug disappear, resolve, stabilize, or the laboratory values return to normal or stabilize.

7.3. Definition of Protocol treatment termination

Protocol treatment completion is defined as completion of treatment for one year in the case of Group A or one year in the case of Group B from the start date of protocol treatment, without meeting the criteria for discontinuation of protocol treatment in Section 7.1 during the study period. See Section 12.2 for a detailed definition of completed treatment.

7.4. Subsequent therapy

If any of the criteria for discontinuation of treatment in "7.1 Criteria for discontinuation of protocol treatment" are met and this treatment is discontinued, the patient should be followed up with supportive care alone until recurrence is clearly recognized, unless there is a special reason. Although treatment in the event of recurrence or the presence of cancerous lesions other than recurrence will not be restricted, the details should be reported in the case report form.

8. Concomitant medications, and concomitant and supportive care

8.1. Prohibited concomitant drugs and prohibited therapies

Other anticancer drugs, flucytosine, phenytoin, warfarin, dabigatran, radiation therapy, immunotherapy, surgery, hormones

8.2. Concomitant medications and concomitant therapies

If Grade 4 leukopenia or neutropenia is observed, G-CSF may be used within the scope of insurance coverage. However, G-CSF agents should not be administered on the same day as anticancer agents. If thrombocytopenia, nausea/vomiting, hypersensitivity, or other adverse events occur, aggressive symptomatic treatment should be given as necessary.

8.3. Testing and supportive care for HBs antigen-negative, HBc antibody-positive and/or HBs antibody-positive cases

The "Guideline for the prevention of hepatitis B caused by immunosuppression and chemotherapy" recommend that HBs or HBc antibodies should be measured even in HBs antigen-negative cases, and that HBV-DNA quantification should be performed in positive cases.

In HBs antigen-negative cases and HBs antibody- or HBc antibody-positive cases, it has been reported that the use of strong immunosuppressive drugs can cause reactivation of HBV, resulting in the development of severe hepatitis. Therefore, based on the "Guideline for the prevention of hepatitis B caused by immunosuppression and chemotherapy" by the Ministry of Health, Labour and Welfare Research Group "Research on refractory liver and biliary tract diseases (PI: Hirohito Tsubouchi)" and "Research on standardization of treatment for viral liver diseases including cirrhosis (PI: Hiromitsu Kumada)", the following tests should be performed before starting chemotherapy, and the following supportive care is recommended according to the test results. It is strongly recommended that a hepatologist should be consulted when administering entecavir. However, patients positive for HBs antibody alone and have a clear history of HBV vaccination should be excluded.

<u>Response to hepatitis B virus in this study</u>
HBs antigen positive → registration prohibited
HBs antigen negative → HBc antibody and HBs antibody negative → Normal response
HBs antigen negative → HBc antibody and HBs antibody positive → Although HBV-DNA quantification is recommended, detailed measures are left to the each institution (nucleic acid analog administration, regular monitoring of hepatitis, etc.)

1) Tests performed before starting chemotherapy: HBV-DNA quantification

HBV-DNA quantification is performed by PCR or real-time PCR method. The real-time PCR method with higher detection sensitivity is preferred.

2) If HBV-DNA quantification is above detection sensitivity at the time before the start of chemotherapy

Give entecavir as in HBs antigen-positive cases.

(1) Test: HBV-DNA quantification

HBV-DNA quantification should be performed every 4 weeks from the start of chemotherapy until 12 months after the end of entecavir administration after completion of chemotherapy. However, if the patient is receiving entecavir and HBV-DNA quantification has less than detection sensitivity, the testing interval may be extended to allow for the duration of outpatient visits.

Testing for HBe antigen and HBe antibodies is also recommended as appropriate.

(2) Drug used: entecavir hydrate tablets (Bristol-Myers: Balaclude tablets 0.5 mg)

According to the dosage and administration listed below, entecavir should be started as soon as possible before and after the start of chemotherapy and continued for at least 12 months after the end of chemotherapy. If treatment with entecavir is to be terminated after 12 months of chemotherapy, the patient should consult a hepatologist to confirm that HBV-DNA quantification is less than detection sensitivity and to decide when to terminate treatment. Patients should be carefully monitored by HBV-DNA quantification, bearing in mind that reactivation is possible even after termination of entecavir treatment.

- Dosage and administration: Orally administered on an empty stomach (at least 2 hours after a meal and at least 2 hours before the next meal).
- Dosage:

Creatinine clearance (mL/min)	Dose
≥ 50	0.5 mg once daily
≥ 30 and < 50	0.5 mg once every 2 days

≥ 10 and < 30	0.5 mg once every 3 days
< 10	0.5 mg once every 7 days

3) HBV-DNA quantification with less than detection sensitivity at the time before chemotherapy
Monitoring by either HBV-DNA quantification or liver function (AST, ALT).

(1) Tests: HBV-DNA quantification or liver function (AST, ALT)
The MHLW Research Group guidelines recommend monitoring by HBV-DNA quantification every 4 weeks during chemotherapy and for 12 months after chemotherapy. However, the guideline was developed based on data from high-risk cases of HBV reactivation, such as chemotherapy with rituximab, and there is limited evidence for solid tumors, so there is room for consideration of cost-effectiveness in chemotherapy cases where the risk of reactivation is expected to be low. Other than periodic HBV-DNA quantification monitoring, a countermeasure method that involves careful liver function (AST, ALT) monitoring and HBV-DNA quantification as appropriate if abnormalities are observed is an option. However, careful monitoring is necessary because there have been reports of failure to save lives (death due to fulminant hepatitis) when antiviral drugs were administered after hepatotoxicity and hepatitis due to HBV reactivation had occurred.
In view of the above background and the risk of HBV reactivation by chemotherapy, monitoring by either HBV-DNA quantification or liver function (AST, ALT) is strongly recommended in this study. If HBV-DNA quantification is above the detection sensitivity, entecavir should be started immediately according to the dosage and administration described in 2) above.

9. Anticipated adverse drug events

9.1. Drug information

Generic name: S-1, Trade name: TS-1, Manufactured and distributed by Taiho Pharmaceutical Co. For details, please refer to the product document. The latest information can be found at http://www.info.pmda.go.jp/.

9.2. Definition of adverse events

An adverse event is any unwanted or unintended sign (including abnormal changes in laboratory values), symptom, or illness that occurs in a subject as a result of receiving the study treatment, whether or not causally related to the study treatment.

9.3. Assessment of adverse events/adverse reactions

The NCI Common Terminology Criteria for Adverse Events v4.0 (Japanese translation; attached) will be followed. An adverse event is defined as a Grade 1 or greater deterioration from the baseline level.

9.4. Anticipated adverse reactions

Refer to the respective attachment. For the latest information, check http://www.info.pmda.go.jp/.

9.5. Causal relationship with study drug

In determining the causal relationship of an adverse event to the study drug, the subject's general condition, complications, concomitant medications/adjunctive therapies, and temporal relationship should be taken into consideration. The causal relationship will be judged by using two categories: "there is a reasonable causal relationship with the study drug" and "there is no reasonable causal relationship with the study drug". Among adverse events, those with a reasonable causal relationship to the study treatment shall be treated as adverse reactions.

10. Reporting adverse event

In the event of a "serious adverse event" or "unanticipated adverse event", the principal investigator in each

institution will report it to the principal investigator/research secretariat.

The responsibility for reporting to the head at each institution, spontaneous reporting from medical institutions to the Pharmaceutical Affairs Bureau of the Ministry of Health, Labour and Welfare under the "Safety Information Reporting System for Drugs and Other Drugs", a Ministry of Health, Labour and Welfare project, and spontaneous reporting from medical institutions to companies under the "Company Reporting System" under the Pharmaceutical Affairs Law shall be handled appropriately by the principal investigator at each institution in accordance with the regulations of each institution. The responsibility of the principal investigator of each institution shall be to make appropriate reports in accordance with the regulations of each institution.

10.1 Adverse events requiring urgent reporting

Adverse events that fall into any of the following categories are subject to urgent reporting.

(1) All deaths during protocol treatment or within 30 days of the last protocol treatment date.
The presence or absence of a causal relationship with the protocol treatment is not required. In the case of discontinued protocol treatment, even if post-treatment has already started, the case is eligible for urgent report if it is within 30 days of the last protocol treatment date ("30 days" refers to 30 days counting from the day after the last protocol treatment date, which is day 0).

(2) Unexpected Grade 4 non-hematologic toxicity (adverse events outside the blood/bone marrow category in CTCAEv.4.0-JCOG): those not described in the most recent product document.

10.2. Adverse events requiring normal reporting

Adverse events that fall into one of the following categories are subject to normal reporting

(1) Deaths after 31 days from the last protocol treatment date for which a causal relationship to protocol treatment cannot be ruled out. Deaths suspected to be treatment-related deaths fall under this category. Obvious deaths of the disease are not applicable.

(2) Anticipated Grade 4 non-hematologic toxicity (adverse events outside the blood/bone marrow category in CTCAEv.4.0-JCOG)

(3) Unexpected Grade 3 adverse events
Grade 3 equivalent adverse events that do not fall under the "Expected adverse drug events" section.

(4) Permanent or significant impairment
Aplastic anemia, myelodysplastic syndrome, secondary cancers, etc.

(5) Other serious medical events
Information that does not fall into any of the above categories, but is deemed important information that should be shared by the principal investigator and all institutions in the research group

10.3. Reporting obligations and procedures of the institutional principal investigators

Urgent report

(1) Primary report: The physician in charge of the case should fill out the "AE/AR/ADR Rapid Primary Report" within 72 hours, fax/telephone the form to the research secretariat, and also report to the institutional principal investigator as soon as possible.

(2) Secondary report: Prepare an "ADR Report Form" and a "Detailed Case Report (A4 free-form)" with details, and fax both to the research secretariat within 7 days.

(3) Tertiary report: In principle, complete all the prescribed items on the "ADR Report Form" and fax a copy to the research secretariat within 15 days of learning of the occurrence of the adverse event.

(4) Additional report: If there is additional information obtained after the tertiary report, such as an autopsy report in the case of death, or if there is additional information that was not completed in the tertiary report, it should be reported to the research secretariat.

Regular Report

(1) Report within 15 days according to the "ADR Report" and in accordance with the urgent report.

10.4. Responsibilities of principal investigator/research secretariat

(1) Determining whether there is a need to suspend registration and emergency notification to the institutions

Upon receiving a report from the principal investigator, the principal investigator or secretariat will determine the urgency, importance, and degree of impact of the report and, if necessary, take measures such as suspending the registration (contacting the registration center and all participating institutions) or urgently notifying the participating institutions of the matter. Depending on the level of urgency, the registration centers and institutions may be contacted by telephone, but will also be notified in writing (fax, mail, e-mail) as soon as possible afterwards.

(2) Report to the Data and Safety Monitoring Committee

If the principal investigator/research secretariat determines that an adverse event reported by an institution as an urgent or routine report constitutes a "reportable adverse event", it should be reported in writing (fax, mail, or e-mail) to the Data and Safety Monitoring Committee within 15 days of learning of the occurrence of the adverse event, and at the same time, the principal investigator/research secretariat should request a review of the principal investigator's opinion regarding the adverse event and the adequacy of the response to the adverse event. At the same time, the Committee will request a review of the opinion of the principal investigator regarding the adverse event and the appropriateness of the response to the adverse event.

11. Observation/test items and timing

11.1. Definition of treatment period

The treatment period for each subject is from the date of enrollment until 4 weeks after the last dose or until the start of post-treatment within 4 weeks of the last dose.

The principal investigator or a research associate investigator will investigate and confirm the following information prior to enrollment and initiation of administration

11.2. Preoperative endpoints

Chest X-ray/CT, head CT or MRI, abdominal CT, bone scintigraphy. PET can be substituted for abdominal and bone lesion search.

11.3. Pre-treatment (at enrollment) endpoints (within 2 weeks prior to enrollment)

1) Patient background

Sex, age, height, weight, body surface area, date of surgery, pathological stage (stage and TNM classification), medical history, PS, histopathology findings, complications and disease name and treatment, concomitant medications, smoking history (number multiplied by duration)

(2) Subjective and objective symptoms

Gastrointestinal symptoms such as nausea/vomiting, fatigue, fever, skin symptoms, dysgeusia, dyspnea, and other symptoms

(3) General blood/biochemistry/gas

General blood (leukocytes/fraction, hemoglobin, platelets)

Blood biochemistry (Alb, T-Bil, AST, ALT, LDH, BUN, Ccr or Cr, Na, K, Cl, Ca, CRP)

Blood gas test or SpO_2

4) Observation of targeted lesions: chest X-ray, chest CT if necessary

5) Tumor markers: CYFRA, CEA

6) Urinalysis: protein, sugar, urobilinogen, occult blood

11.4. Endpoints during treatment period

Evaluate various test items and the subject's condition.

1) General condition:	PS, weight measurement
2) Subjective and objective symptoms:	Same as 11.3 (2)
3) General blood/biochemistry/gas:	Same as 11.3.(3)

4) Observation of targeted lesions: Same as 11.3.(4)
5) Tumor markers: Same as 11.3.(5)
6) Urinalysis: Same as 11.3.(6)

Item/Period	Before the start of treatment	During treatment (every 1-2 weeks)	At the end of treatment
Patient background	○		
Subjective and objective findings and PS	○	○	○
Target lesion observation (Search for new lesions)	○	In principle, every 2 months (Enforced as needed)	○
General blood/biochemistry/gas and weight measurement	○	○	○
Tumor marker	○	In principle, every 1 month*	○
Urinalysis	○	as necessary	as necessary

If any adverse events occur, they should be treated as often as possible to allow adequate observation of their progress (e.g., most abnormal, date of recovery or lightening), even if they occur on days other than those specified in all the above items.
*This will be performed within the scope of insurance coverage.

11.5. After discontinuation/termination of drug administration

After discontinuation/termination of treatment to a total of 2 years after the first administration

As a rule, an examination and chest X-ray should be performed every 3 months. Imaging tests including chest CT, tumor markers (CEA, CYFRA) every 3 to 6 months, and blood tests (hemoglobin, white blood cell count and fraction, platelet count, Alb, T-Bil, AST, ALT, LDH, BUN, Cr, Na, K Cl, Ca) as necessary.

After discontinuation/termination of treatment to a total of 3 to 5 years after the first administration

As a rule, an examination and chest X-ray should be performed every 6 months. Imaging tests including chest CT, tumor markers (CEA, CYFRA) every 6 to 12 months, and blood tests (hemoglobin, white blood cell count and fraction, platelet count, Alb, T-Bil, AST, ALT, LDH , BUN, Cr, Na, K, Cl, Ca) as necessary.

11.6. With signs of recurrence

At any time after surgery, any sign of recurrence should be investigated (chest CT, bone scintigraphy, head CT or MRI, abdominal CT, PET scan if necessary, etc.).

12. Evaluation methods of assessment items

12.1. Evaluation items

Primary endpoint : Treatment completion rate (Feasibility)
Secondary endpoints: rate and severity of adverse events, recurrence-free survival (RFS), overall survival (OS)

12.2. Evaluation method

Treatment completion rate (Feasibility)

Definition of treatment completion

Patients who have completed six months of treatment with S-1 as postoperative chemotherapy with a dose reduction/withdrawal ratio of at least 70% while following the dose reduction/withdrawal criteria specified in the protocol are defined as those who have completed the protocol treatment (completed administration).

S-1 actual total dose/planned total dose ratio

Percentage of doses administered (%) = Total actual prescriptions during the entire dosing period (mg)/total scheduled doses (mg) x 100

However, total expected dose = initial dose (mg/day) x total expected days (days), and

Defined as the sum of the actual doses taken during the entire treatment period (mg) = Σ(total doses taken up to 6 months or until treatment discontinuation), where Σ(total doses taken up to 6 months or until treatment discontinuation).

Finally, for the primary endpoint, the proportion of patients who "completed the protocol" to all eligible patients will be calculated, excluding those who discontinued or dropped out of the protocol due to recurrence. The completion rate at 9 months, 1 year, etc. of protocol treatment will also be calculated.

Recurrence-free survival (RFS)

削除: Relapse

The period beginning on the date of registration and ending on the earlier date on which the patient is determined to have relapsed or the date of death from any cause.

The term "recurrence" includes both diagnostic imaging and clinical relapse, which is defined as an exacerbation of a disease condition that is not diagnosed by imaging. In the case of a recurrence determined based on imaging diagnosis, the date of recurrence is the date of the examination that performed the imaging test. A period of elevated tumor markers alone shall not be considered as recurrence, but the date of examination when recurrence is confirmed by diagnostic imaging or the date of clinical judgment of recurrence due to worsening of the disease condition shall be considered as recurrence.

削除: relapse

In the case of a surviving patient who has not been judged to have relapsed, the last date of confirmation of survival shall be the date of termination. (Survival confirmation by phone call is also acceptable, but the fact that survival confirmation was made must be recorded in the medical record.)

In cases where chemotherapy is discontinued due to toxicity or patient refusal, and other treatments are added as post-treatment, the event and discontinuation are treated in the same manner. In other words, the discontinuation is not considered at the time of treatment discontinuation or at the date of start of post-treatment.

When the diagnosis of recurrence is based on diagnostic imaging, the event is defined as the "date of examination" of the imaging test on which a "confirmed diagnosis" is obtained at a later date, not the date of examination of the "suspected diagnosis on imaging". If the recurrence is clinically determined to be a recurrence without imaging diagnosis, the event shall be defined as the date when the recurrence is determined to be a recurrence.

When the definitive diagnosis of recurrence is made by biopsy pathology, the event shall be the date of clinical diagnosis if the recurrence can be diagnosed clinically before biopsy, or the date of biopsy if the recurrence cannot be diagnosed clinically but is diagnosed by biopsy pathology.

The occurrence of a second cancer (iatrogenic overlapping cancer) is neither an event nor a censored event, but rather a recurrence-free survival period until another event is observed.

Differences in recurrence-free survival stratified by completion rate will also be identified.

Overall survival (OS)

The period beginning on the date of the first postoperative administration of S-1 and ending on the date of death from any cause.

In surviving cases, the last date of confirmation of survival is the date of termination (telephone confirmation of survival is also acceptable, but should be documented in the patient's medical record). (Survival confirmation by phone call is also acceptable, but the fact that the patient survived must be recorded in the patient's medical record.)

In the case of untraceable cases, the last date of confirmed survival prior to the loss of follow-up is considered to be the termination date.

Differences in survival stratified by percentage of completion will also be identified.

Percentage of occurring adverse events (adverse reactions)

Using all treated cases as the denominator and the incidence of adverse events judged to be caused by the study treatment as the numerator, the proportion of the worst grade in the entire course according to the CTCAE v4.0 of the Japanese translation JCOG version is determined.

13. Target cases and study period

13.1. Case accumulation period/follow-up period

Case collection period: May 2012 - April 2016 (4 years)
Follow-up period for primary endpoint: 6 months after the end of enrollment
Research period: April 2012 - June 2022

All endpoints will be analyzed 6 months after the end of enrollment, when the protocol treatment and completion rates of all enrolled patients have been assessed. Survival analyses for secondary endpoints will be conducted at 2 years and at the end of the 5-year follow-up period after the end of enrollment. The data center will be responsible for conducting the analyses until the second year after the completion of the final case enrollment, and the secretariat will conduct the analyses thereafter, to be finalized upon consultation with the secretariat. After the analysis of the primary endpoint of the study is completed, the data center will, upon request of the secretariat, transfer to the secretariat free of charge all data information and analysis results related to the study that exist at the data center, as long as it is judged that there will be no bias in the publication of the results or in the analysis of secondary endpoints. The data center shall transfer all data information and analysis results related to the study that exist at the data center to the secretariat free of charge upon request of the secretariat.

13.2. Selection of subjects for analysis

In this study, all enrolled cases, all eligible cases, and all treated cases are defined as follows: "all treated cases" are used for safety evaluation in the final analysis, and "all enrolled cases" or "all eligible cases" are used for efficacy evaluation.

1) All registered cases

The population of registered patients, excluding duplicate or mis-registered patients.

2) All eligible cases

The population excluding "ineligible cases" considered by the group review from the total enrolled cases. However, for periodic monitoring and analysis at the time of conference presentation prior to the submission of the final analysis report, "ineligible cases" may not be included in the total eligible cases with the consent of the study office.

3) All treated cases

All enrolled patients who received some or all of the protocol treatment.

13.3. Basis for setting the number of cases

When conducting an analysis of the primary endpoint using the "Definition of the percentage of treatment completion" in the "Evaluation Methodology" section of the endpoints, cases of protocol discontinuation due to relapse should be treated as discontinuations (dropouts), and are not appropriate as eligible cases for the analysis. The percentage of patients who "completed treatment" was calculated based on the number of patients who were excluded from the total number of eligible cases.

In the LOGIK0601 study of S-1 80 mg/m^2/day 2-week administration followed by 1-week rest (6 months administration) in patients with Stage IB or more completely resected non-small cell lung cancer, treatment completion was 56.7% (95% CI: 37.4-74.5, relative dose rate 73.9) and the completion rate in patients 70 years and older was 42.9% (6/14)[16)] . On the other hand, although the data are not available for elderly patients, the use of S-1 every other day (1 year) after gastric cancer surgery resulted in 19.6 % and 13.7 % higher compliance and RDI, respectively, than the usual administration regimen (1 year) (91.8% vs 72.2% and 81.2% vs 67.5%, respectively) .[15)]

Assume that 55% of the patients in the better group and 40% of the patients in the worse group complete treatment for 6 months with the biweekly or 2-week administration followed by 1-week rest method. Based on the selection theory, 37 cases in each group are required to detect this 15% difference with a 90% probability of correct selection. Assuming dropout cases, a total of 100 cases are required. The expected annual enrollment is 50 cases, and the enrollment period is 2 years.

In order to achieve ITT analysis, Kaplan-Meyer curves for the actual total number of days of S-1 administration in each case were drawn for all enrolled patients. An event is considered to have occurred at the time of discontinuation due to S-1 toxicity. If the drug is discontinued due to complications (recurrence, second cancer) that are not directly related to S-1, the event is considered to have occurred at that time.

The continuation rate calculated from the Kaplan-Meyer curve will also be evaluated as a secondary measure of compliance with the study treatment.

13.4. Decision criteria for standard treatment (S-1 administration) based on the results

This study was designed to verify that alternate-day administration is a superior method of administration with better continuity than the daily administration method. However, the difference in administration continuity alone does not necessarily mean that the true objective of adjuvant chemotherapy, namely, survival benefit, is achieved.

Therefore, at the end of the trial, the decision criteria for selecting either study arm for future Phase III trials shall be as follows.

(1) If the six-month completion rate for both groups is less than 40%, we conclude that both groups are not promising adjuvant chemotherapy in this study.

(2) If the completion rate of six-month treatment in one group exceeds 40% and is more than 15% higher than that in another group, we conclude that the treatment in the group with the higher completion rate of six-month treatment is the most promising adjuvant chemotherapy in the study and is more suitable for the administration of S-1 in future Phase III trials.

(3) If the completion rate in one group is greater than 40% and the difference between one group and the other is within 15%, the two groups will not be directly compared based on retention rate numbers alone, but rather on toxicity, quality of life, convenience, cost, retention rate after 6 months, recurrence-free survival, and survival rate to determine which is more suitable as S-1 therapy in future Phase III trials.

14. Discontinuation of the study

Enrollment will be terminated when the expected number of enrolled patients is reached, but enrollment will be accepted for eligible patients who have already been informed of their participation in the study at that time. If, during the course of the study, it is determined that patient safety is seriously compromised and the entire study must be terminated due to a serious adverse event or new information on the study drug or treatment, the Data and Safety Monitoring Committee will recommend to the principal investigator that the study should be terminated. The principal investigator will review the recommendation and make a decision to discontinue

the study with the approval of the Clinical Trial Review Committee. After the decision to discontinue the study, the principal investigator will promptly inform the participating institutions of the discontinuation and the reason for discontinuation in writing.

15. Data collection and storage

15.1. Data collection

The Case Report Forms (CRFs) to be used in this study and the submission deadlines are as follows. In principle, all CRFs below should be sent by mail. All CRFs should be sent to the data center.

(1) Basic subject data: Promptly after registration
(2) Anticancer Drug Administration Record Form: At the time of discontinuation of treatment/within 2 weeks after completion
(3) Treatment Termination Report Form: At the time of discontinuation of treatment/within 2 weeks after completion
(4) Follow-up: Every 1 year or upon confirmation of recurrence
(5) Blood tests, abnormal subjective and objective findings, abnormal values, etc.: At the time of discontinuation of treatment/within 2 weeks after completion
(6) Tracking Report Form: At the time of request of the secretariat

15.2. Preservation of records

Records shall be kept at the data center in accordance with the "Standard Procedures" for record keeping, and documents and records related to the study shall be kept at the data center. The retention period shall be 5 years after the date of the report of the completion of the study or 3 years after the date of the final publication of the results of the study, whichever is later. Patient consent records, data related to the preparation of reports (e.g., laboratory data), IRB/IEC approval forms, and records and documents prepared at participating institutions will be retained by the investigator. The retention period will be 5 years from the date of the report of the completion of the study or 3 years from the date of the final publication of the results of the study, whichever is later.

16. Ethical matters

16.1. Protection of subjects

All researchers involved in this research will conduct the research in compliance with the "Declaration of Helsinki" and the "Clinical Research Act". When handling information related to the implementation of the research, we will assign a research subject code unique to the research and give due consideration to the protection of the confidentiality of the research subjects. When publishing the results of the research, information that can immediately identify the research subjects, such as names and dates of birth, will not be included. In addition, information on research subjects obtained in the research will not be used for any purpose other than the purpose of the research.

16.2. Consent obtained

Prior to enrolling subjects, the attending physician will provide a thorough explanation of the following items using the consent document The subject will be given the opportunity to ask questions and sufficient time to decide whether to participate in the study. After confirming that the subject fully understands the study, the subject's free and voluntary consent to participate will be obtained in writing. The attending physician will promptly hand a copy of the signed consent document to the subject. The original consent document will be kept in the medical record. The period of retention will be 5 years after the date of the report of the completion of the study or 3 years after the date of the final publication of the study, whichever is later.

16.3. Explanation

1) Background, 2) Purpose of this study, 3) Method/content of treatment in this clinical trial, 4) Expected medical contribution, 5) Duration of this study, 6) Number of expected

participants in this study/place of study, 7) Expected effects and side effects, 8) Other effective treatment methods, 9) Samples used in the study, 10) If your health is harmed; 11) Free and voluntary consent; 12) If new significant information is obtained; 13) Possibility of discontinuation during the clinical trial; 14) Protection of personal information, protection of human rights, and disclosure of research results; 15) Things you should be aware of; 16) Your responsibility for the cost of treatment; 17) research funding, conflicts of interest, and research benefits; 18) benefits, disadvantages, and risks you may receive if you participate in this study; 19) secondary use of data; 20) contact information

16.4. Privacy protection and subject identification

Identification and matching of registered patients will be performed using the registration number issued at the time of registration, and the names of registered patients will not be shared with other facilities. In addition, patient names, medical record numbers, and other personally identifiable information will not be registered with the secretariat. Although the possibility of leakage of personal information cannot be completely eliminated, physicians participating in this study will make their utmost efforts to protect personal information in light of the possible risks.

16.5. Compliance with protocols

Investigators participating in this study will comply with this study protocol to the extent that patient safety and human rights are not compromised.

16.6. Approval of Institutional Review Board (IRB) or Ethics Review Board

Upon approval by the IRB or Ethics Review Board, the principal investigator at each institution will send a copy of the approval letter to the research secretariat. The original approval letter will be retained by the institution and a copy will be retained by the research secretariat.

16.7. Changes in protocol content

Classification of change in protocol content

In the event of a change in protocol content, a "Protocol Revision Application" must be submitted to the Data and Safety Monitoring Committee for approval prior to implementation of the change.

Changes in protocol content are divided into two types: amendments and revisions. The distinction between amendments and revisions is made by the Data and Safety Monitoring Committee members. Supplemental explanatory notes that do not fall under the category of changes in protocol content are distinguished as memorandums.

(1) Amendment: Partial changes that may increase the risk to patients participating in the study or with respect to the primary endpoint.

(2) Revision: Changes that are not likely to increase the risk to patients participating in the study and are not related to the primary endpoint.

(3) Memorandum/Memorandum of Understanding: A supplement to the protocol distributed by the principal investigator/research secretariat to study personnel, not to change the content, but to unify variations in interpretation of the text, especially to call attention to them, etc.

Institutional IRB or Ethics Review Board approval at the time of protocol amendment/revision

If any amendments are made to the study protocol or patient instructions with the approval of the Data and Safety Monitoring Committee members during the study, the amended study protocol or patient instructions must be approved by the IRB or Ethics Review Board at each site.

If the content change is a revision rather than an amendment, it is up to each institution to determine whether it requires approval review by its IRB or Ethics Review Board.

Upon IRB or Ethics Review Board approval of the amendment, the principal investigator at each site will send a copy of the approval letter to the research secretariat. The original approval letter will be retained by the institution and a copy will be retained by the secretariat.

16.8. Changes in the contents of the case report form (CRF)

If, after the start of the study, the CRF is found to be incomplete, such as missing required data items or inappropriate categorization, etc., as long as it is determined that the scope of data collected does not exceed that specified in the "Observation, Examination, and Evaluation Items and Schedule" section, and the modification of the CRF will not increase the medical and financial burden on enrolled patients The CRF will be revised upon agreement between the Data and Safety Monitoring Committee and the research secretariat. The reporting of CRF amendments to the head of the medical institution and the application for revision of the CRF shall be in accordance with the regulations of the institution.

16.9. Secondary use of data

For data obtained in this study, there may be secondary use of data (e.g., meta-analysis) in a form that is not linked to personally identifiable information.

17. Monitoring and auditing

Monitoring will be conducted as necessary, led by the research secretariat/principal investigator, to ensure that the study is being conducted safely and according to protocol and that data are being collected accurately. The CRF is prepared by the principal investigator/associate investigator or CRC (clinical research coordinator). Monitoring will be conducted centrally based on the data entered in the CRF collected at the research secretariat, and will not include site visits for site monitoring, including cross-checking with source documents. No directly identifiable patient information will be entered into the database of the secretariat. Maximum care will be taken to ensure that personal information is strictly protected.

17.1. Monitoring items

Accumulation achieved, patient appropriateness, percentage of treatment completed, protocol treatment/completion status, serious adverse events, adverse reactions/adverse events, protocol deviations, and other issues related to study progress and safety.

17.2. Deviation from protocol

(1) Violation: As a rule, a "violation" is defined as any deviation from the protocol provisions that falls under more than one of the following items.

(1) affects the primary endpoint, (2) is caused by the physician/institution, (3) is intentional or systematic, (4) the degree of risk or deviation is extreme, or (5) is clinically inappropriate.

(2) Deviations: Deviations that do not fall under either violations or tolerances. If many specific deviations are found, they shall be noted in the publication of the test results.

(3) Acceptable scope: To be determined in advance between the research group and the research secretariat.

18. Costs and compensation

Since S-1 used in this study is already covered by insurance for non-small cell lung cancer, the cost of the treatment will be covered by the regular insurance reimbursement. Any adverse health effects resulting from the study treatment will be handled under general medical care and will be borne partially by the subject, as with insurance treatment. No special compensation will be provided for hospitalization or transportation costs. It is recommended that physicians participating in the study obtain medical liability insurance.

19. Research funding and conflicts of interest

Funding for this study will be provided by a non-profit organization Epidemiological & Clinical Research Information Network (ECRIN).

In the design, conduct, and reporting of this study, there are no "possible conflicts of interest" so as to affect the results of the study and the interpretation of the results and that the conduct of the study will not prejudice the rights and interests of the subjects.

20. Method of disclosing information on the study (registration of study plans and publication of study results)

This study will be registered in the Japan Registry of Clinical Trials (jRCT) for the publication of clinical research protocols and research summaries. In addition, the results obtained from this study will be presented at major domestic and international academic conferences and published as articles in English-language professional journals. In both cases, only statistically processed results will be published. In principle, the author(s) of the published paper will be selected by the principal investigator or facility

coordinator for each institution in the order of the number of enrollments, with the final decision being made by the principal investigator.

The principal investigator shall prepare a primary endpoint report and a summary report and brief overview.

(1) Primary endpoint report: within one year after the end of the period for collecting data on the main evaluation item.

(2) Summary report and brief overview: within one year after the end of the period for collecting data on all evaluation items.

The report will be submitted to the Certified Review Board to hear its opinion, and will be made publicly available on jRCT within one month from the date the Certified Review Board gives its opinion. In addition, a summary of the research protocol and summary report shall be submitted to the Minister of Health, Labour and Welfare.

When the principal investigator publishes a summary of the primary endpoint report or summary report, he/she shall promptly report it to the administrator of the implementing medical institution and provide information to the other principal investigators to that effect. In this case, said other principal investigators shall promptly report the details of said provision of information to the administrator of the implementing medical institution.

21. Analysis for quality of life

The program will be conducted at institutions where participation is possible.

21.1. Required samples

Attached QOL questionnaire (sample) “Care Notes”

21.2. Method of investigation

Patients complete a prescribed QOL questionnaire over a 12-month period from enrollment.

21.3. Sample delivery

Once the relevant patient has completed the form, the physician in charge will mail it to the data center.

21.4. Sample analysis

Analysis will be conducted at the Department of Biomedical Statistics and Bioinformatics, Kyoto University Graduate School of Medicine. The QOL survey will be analyzed at the Department of Respiratory Medicine, Okayama University Hospital.

21.5. Post-analysis processing of samples

Samples will be stored at the Department of Thoracic Surgery, Okayama University Hospital for 5 years after the end of this study after analysis is completed.

22. Disease reports

In the event of illness, death, disability, or infection suspected to have resulted from the conduct of this research, a report will be made to the Certified Review Board and, in the case of unexpected serious cases, to the Minister of Health, Labour and Welfare.

Serious illnesses, etc. suspected to be due to the conduct of this study are defined as follows

(1) Death
(2) Diseases, etc. that may lead to death
(3) Diseases requiring hospitalization or extended hospitalization for treatment, etc.
(4) Disability
(5) Diseases, etc. that may lead to disability
(6) Diseases (3) to (5) inclusive and diseases that are as serious as death or diseases that may lead to death
(7) Congenital diseases or anomalies in later generations

When a principal investigator and a sub investigator (principal investigator, etc.) learn of the occurrence of a

serious illness, etc. in the conduct of Specified Clinical Research, they must take the necessary measures, including explanations to the research subjects, etc.
Since this is a multicenter collaborative study, the principal investigator will be responsible for reporting illnesses, etc. When the principal investigator becomes aware of an outbreak of illness, etc., he/she will report it to the administrator of the implementing medical institution, notify the principal investigator, and promptly provide information to other principal investigators. In such cases, the other principal investigators shall promptly report the details of the provision of said information to the administrator of the implementing medical institution. The principal investigator must report the report of disease, etc. to the authorized clinical research review committee described in the implementation plan of said specific clinical research using the Uniform Form 8, as specified by an Ordinance of the Ministry of Health, Labour and Welfare. The reporting deadline is as follows.

Predictability	Diseases, etc.	Report to the Certified Review Board	Report to the Minister of Health, Labor and Welfare
Unpredictable	Above (1) and (2)	7 days	7 days
	Above (3) to (7)	15 days	15 days
Predictable	Above (1) and (2)	15 days	–
	Above (3) to (7)	At the time of periodic report	–

23. Research organization

23.1. Principal investigator (Setouchi Lung Cancer Study Group)

Shinichi Toyooka, Department of General Thoracic Surgery and Breast and Endocrinological Surgery, Okayama University Graduate School of Medicine, Dentistry and Pharmaceutical Sciences

23.2. Research secretariat

Department of Thoracic Surgery, Okayama University Hospital
Tel: 086-235-7265, Fax: 086-235-7269

23.3. Planned participating institutions

Department of Thoracic Surgery, Okayama University Hospital
Department of Thoracic Oncology, Kansai Medical University Hospital
Department of General Thoracic Surgery, Kawasaki Medical School Hospital
Department of Thoracic Surgery and Internal Medicine, Kurashiki Central Hospital, etc.

23.4. Data and Safety Monitoring Committee

Masahiro Tabata: Center for Clinical Oncology, Okayama University Hospital
Kenji Nishii: Okayama Health Foundation Hospital
Takeshi Nagayasu: Nagasaki University Graduate School of Biomedical Sciences

23.5. Center for statistical analysis/registration

Keitaro Matsuo, Department of Preventive Medicine, Kyushu University Faculty of Medical Sciences (until study protocol version 1.4)
Satoshi Morita, Department of Biomedical Statistics and Bioinformatics, Kyoto University Graduate School of Medicine (from study protocol version 1.5)

23.6. Data center

A non-profit organization Epidemiological & Clinical Research Information Network (ECRIN)
Director: Junichi Sakamoto
Japan Mutual Aid Association of Public School Teachers Tokai Central Hospital
Aichi Branch: Keiko Arai, Yumi Miyashita
Tel: 0564-64-7300, Fax: 0564-64-7301

24. References

1) N Engl J Med 2004; 350: 351-60.
2) N Engl J Med 2005; 352: 2589-97.
3) Lancet Oncol 2006; 7 :719-27.
4) J Clin Oncol 2005; 23: 4999-5006.
5) Cancer and Chemotherapy 1998;25: 371-384.
6) Anticancer Drugs 1996;7: 548-557.
7) Br J Cancer 2001;85: 939-943.
8) The 50th Japanese Lung Cancer Conference (2009), P-194, Kimihiro Shimizu et al., Gunma University Graduate School of Medicine
9) Br J Cancer 2005;93:884.
10) Gastroenterogy 1963;45;721-9.
11) Cancer 1965;18:1189-213.
12) Little Brown & Co. 1960;56:70-98.
13) Int J Clin Oncol. 2004;9:143-8.
14) The 49th Annual Meeting of Japan Society of Clinical Oncology (2011), OS38-1, Yamagami et al., Wakayama Medical University
15) S. Nakamura et al. ASCO-GI2012 #114 "Randomized controlled phase II study of alternate-day S-1 as adjuvant chemotherapy for gastric cancer."
16) Lung Cancer 2010;67:184-187.
