# Supplementary material for: Randomized phase II study of daily versus alternate-day administrations of S-1 for the elderly patients with completely resected pathological stage IA (tumor diameter > 2 cm)—IIIA of non-small cell lung cancer: Setouchi Lung Cancer Group Study 1201
Source: PLoS One. 2023 May 19;18(5):e0285273. doi: 10.1371/journal.pone.0285273 (PMC10198543; doi:10.1371/journal.pone.0285273)
Supplement: S2 File — (PDF) [file pone.0285273.s025.pdf]

Randomized phase II study of daily versus alternate-day administration of S-1 for the elderly patients with completely resected pathological stage IA (T1bN0M0)/IB/II/IIIA non-small cell lung cancer

Study protocol (separate sheet)

1. Name of the medical institutions from which and to which the information is provided

Provider medical institutions

Norihito Okumura, Chief, Department of Thoracic Surgery, Kurashiki Central Hospital, 1-1-1 Miwa, Kurashiki, Okayama 710-8602
Tel: 086-422-0210
Number of registered cases: 26

Yutaka Shio, Senior Assistant Professor, Department of Chest Surgery, Fukushima Medical University Hospital, 1 Hikarigaoka, Fukushima 960-1295
Tel: 024-547-1111
Number of cases registered: 11

Shinichi Toyooka, Professor, Department of Thoracic Surgery, Okayama University Hospital, 2-5-1 Shikata-cho, Kita-ku, Okayama 700-8558
Tel: 086-235-7265
Number of registered cases: 9

Masao Nakata, Professor, Department of General Thoracic Surgery, Kawasaki Medical School Hospital, 577 Matsushima, Kurashiki, Okayama 701-0192
Tel: 086-462-1111
Number of cases registered: 7

Motoki Matsuura, Chief, Department of Thoracic Surgery, Hiroshima City Hiroshima Citizens Hospital, 7-33 Motomachi, Naka-ku, Hiroshima 730-8518
Tel: 082-221-2291
Number of cases registered: 6

Kenichi Gemba, Chief, Department of Respiratory Medicine and Oncology, Chugoku Central Hospital, 148-13 Kamiiwanari, Miyuki-cho, Fukuyama, Hiroshima 720-0001
Tel: 084-970-2121
Number of cases registered: 6

Isao Sano, Chief, Department of Respiratory Surgery, Japanese Red Cross Nagasaki Genbaku Hospital, 3-15 Morimachi, Nagasaki 852-8511
Tel: 095-847-1511

Number of registered cases: 5

Takuji Fujinaga, Chief, Department of General Thoracic Surgery, National Hospital Organization Nagara Medical Center, 1300-7 Nagara, Gifu 502-8558
Tel: 058-232-7755
Number of cases registered: 5

Masafumi Kataoka, Director, Department of Surgery and Respiratory Center, Okayama Saiseikai General Hospital, 2-25 Kokutai-cho, Kita-ku, Okayama 700-8511
Tel: 086-252-2211
Number of cases registered: 5

Yasuhiro Terazaki, Chief, Department of Respiratory Surgery, Saga-Ken Medical Centre Koseikan, 400 Kasemachinakabaru, Saga 840-8571
Tel: 0952-24-2171
Number of cases registered: 3

Hiroshi Date, Professor, Department of Thoracic Surgery, Kyoto University Hospital, 54 Shogoinkawahara-cho, Sakyo-ku, Kyoto 606-8507
Number of registered cases: 3
Tel: 075-751-3111

Nobukazu Fujimoto, Chief, Department of Medical Oncology and Respiratory Medicine, Okayama Rosai Hospital, 1-10-25 Chikkomidorimaschi, Minami-ku, Okayama 702-8055
Tel: 086-262-0131
Number of registered cases: 3

Kazuhiko Kataoka, Chief, Department of Thoracic Surgery, National Hospital Organization Iwakuni Clinical Center, 1-1-1 Atagomachi, Iwakuni, Yamaguchi 740-8510
Tel: 0827-34-1000
Number of registered cases: 3

Shinji Kosaka, Director, Department of Thoracic Surgery, Shimane Prefectural Central Hospital, 4-1-1 Himebara, Izumo, Shimane 693-8555
Tel: 0853-22-5111
Number of cases registered: 2

Motohiro Yamashita, Deputy Director, Thoracic Surgery, National Hospital Organization Shikoku Cancer Center, 160 Minamiumemotomachi-Ko, Matsuyama, Ehime 791-0280
Tel: 089-999-1111
Number of registered cases: 2

Hidetoshi Inokawa, Chief, Department of Thoracic Surgery, National Hospital Organization Yamaguchi-Ube Medical Center, 685 Higashi-kiwa, Ube, Yamaguchi 755-0241
Tel: 0836-58-2300
Number of registered cases: 2

Masaaki Inoue, Chief, Department of Chest Surgery, Shimonoseki City Hospital, 1-13-1 Koyocho, Shimonoseki, Yamaguchi 750-8520
Tel: 083-231-4111
Number of cases registered: 1

Hiroshige Nakamura, Professor, Division of General Thoracic Surgery, Tottori University Hospital, 36-1 Nishi-cho, Yonago, Tottori 683-8504
Tel: 0859-33-1111
Number of registered cases: 1

Yoshinori Yamashita, Chief, Department of Thoracic Surgery, National Hospital Organization Kure Medical Center and Chugoku Cancer Center, 3-1 Aoyamacho, Kure, Hiroshima 737-0023
Tel: 0823-22-3111
Number of registered cases: 1

2. Sample/information items

noted

3. History of acquisition of samples and information

noted

4. How to manage the correspondence table

(1) Samples and information obtained in this study

The principal investigator at each institution will manage the data appropriately and will not provide the data to outside parties.
