# Supplementary material for: Randomized phase II study of daily versus alternate-day administrations of S-1 for the elderly patients with completely resected pathological stage IA (tumor diameter > 2 cm)—IIIA of non-small cell lung cancer: Setouchi Lung Cancer Group Study 1201
Source: PLoS One. 2023 May 19;18(5):e0285273. doi: 10.1371/journal.pone.0285273 (PMC10198543; doi:10.1371/journal.pone.0285273)
Supplement: S3 File — (PDF) [file pone.0285273.s026.pdf]

瀬戸内肺癌研究会
SLCG1201

高齢者非小細胞肺癌完全切除後病理病期 IA(T1bN0M0)/IB/II/IIIA 期症例の術後補助化学療法に対する S-1 の連日投与法および隔日投与法のランダム化第二相試験

実施計画書

瀬戸内肺癌研究会代表者
豊岡伸一
岡山大学大学院医歯薬学総合研究科
呼吸器・乳腺内分泌外科学

SLCG1201 事務局
諏澤憲、山本寛斉、岡﨑幹生
岡山大学病院　呼吸器外科

プロトコル作成者
吉岡弘鎮[1]、堀田勝幸[2]、豊岡伸一[3]
関西医科大学附属病院　呼吸器腫瘍内科[1]
岡山大学病院　新医療研究開発センター[2]　呼吸器外科[3]

2012 年 2 月 25 日：幹事会コンセプト承認
2012 年 3 月 15 日：計画書 version1.0
2012 年 7 月 10 日：計画書 version 1.1
2013 年 1 月 4 日：計画書 version 1.2
2014 年 3 月 4 日：計画書 version 1.3
2017 年 5 月 24 日：計画書 version 1.4（指針改正に係る修正のため補遺のみで対応）
2018 年 4 月 29 日：計画書 version 1.5
2018 年 10 月 13 日：計画書 version 1.6
2019 年 11 月 17 日：計画書 version 1.7
2020 年 10 月 1 日：計画書 version 1.8

0. 概要

0.1. 試験の概要（シェーマ）

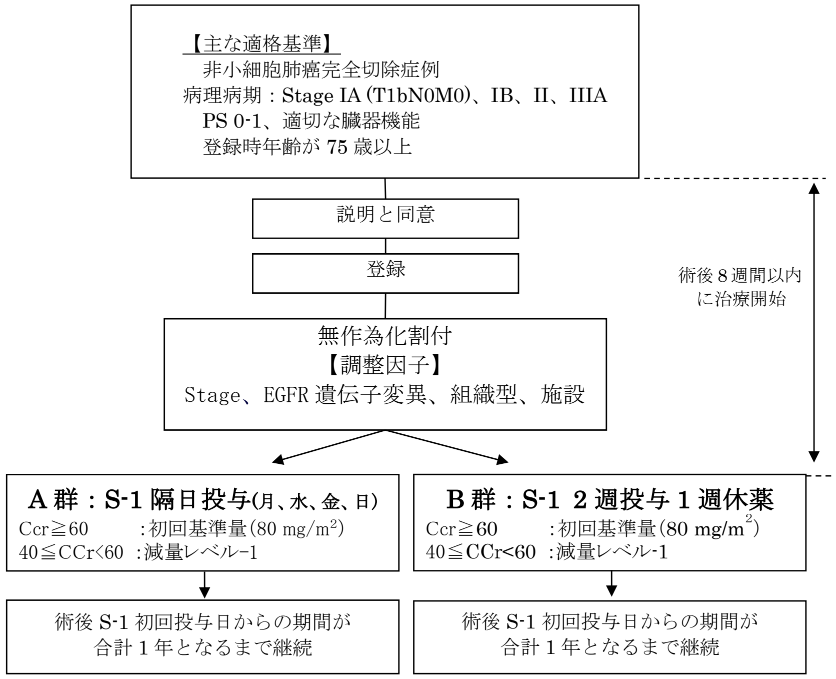

0.2. 目的

病理病期 IA(T1bN0M0)/IB/II/IIIA 期の高齢者非小細胞肺癌完全切除例に対する術後化学療法として S-1 隔日投与の認容性・有効性を検討する。

Primary endpoint　：投与完遂率（Feasibility）
Secondary endpoint ：有害事象発生割合と程度、無再発生存期間(RFS)、全生存期間(OS)、QOL

0.3. 治療

A 群：S-1 隔日投与

S-1 投与は月、水、金、日に行うこととし、治療中止基準のいずれかに該当するまで投与を繰り返す。投与する曜日はずらさない。S-1 隔日投与による治療は、S-1 初回投与日から起算して 1 年間行う。

投与最終日から 28 日を超えても投与が開始できない場合は本試験治療を中止する(最終投与翌日を 1 日目とする。最終投与日の 4 週間後の同じ曜日の投与は可とする)。

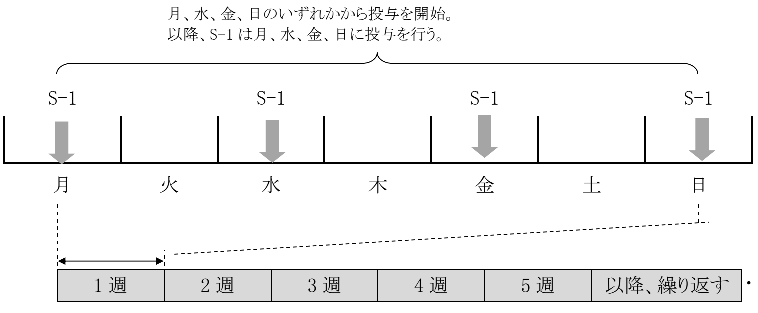

B 群：S-1 2 週投与 1 週休薬

S-1 を第 1 日目～14 日間連日経口内服（第 1 日目の夕食後～第 15 日朝食後）し、その後 7 日間休薬、これを 3 週間ごとに 1 コースとして、繰り返す。プロトコル治療開始日からの治療期間が計 12 ヵ月となるまで行う。プロトコル治療開始日から 12 ヵ月後の同じ暦日以降は新たなコースに入らないこととする。最終コースは day14 相当日まで投与を行うこと。投与最終日から 28 日を超えても投与が開始できない場合は本試験治療を中止する（最終投与翌日を 1 日目とする。最終投与日の 4 週間後の同じ曜日の投与は可とする）。

試験治療の中止基準のいずれかに該当するまで投与を繰り返す。

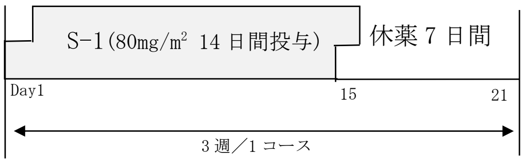

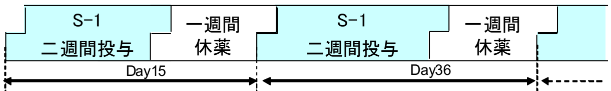

0.4. 対象

0.4.1. 対象疾患

病理病期 IA(T1bN0M0)/IB/II/IIIA 期の高齢者非小細胞肺癌完全切除例。

0.4.2. 選択基準

1）病理学的に非小細胞肺癌と診断された病理病期 IA(T1bN0M0)/IB/II/IIIA 期の症例。ただしカルチノイド、粘表皮癌、腺様嚢胞癌などの低悪性腫瘍を除く。
2） 肺葉切除以上の外科切除が行われた症例で、リンパ節郭清が行われ(原則として ND2a 以上)、病理学的に完全切除を確認できた症例。
3）原発巣に対して手術以外の前治療がない症例。
4）年齢が登録時に 75 歳以上（満年齢）の症例。
5）ECOG の Performance Status（PS）が 0～1 の症例
6）重篤な術後合併症がなく、検査値が以下の基準を満たすこと。
・白血球数：3,000～12,000/mm^3 未満
・血小板数：10×10^4/mm^3 以上
・血色素量：9.0g/dl 以上
・総ﾋﾞﾘﾙﾋﾞﾝ：1.5mg/dl 以下
・AST（GOT）、ALT（GPT）：100(IU/L)未満
・PaO2：60mmHg 以上（もしくは SpO2　90%以上でも可）
・クレアチニン：1.2mg/dl 未満
・クレアチニンクリアランス（CCr）値※：40 mL/min 以上
※登録に当たっては、24 時間蓄尿によるクレアチニンクリアランス実測値、または Cockcroft-Gault 式の推定値を用いる。ただし実測値を優先する。推定値で 50mL/分であった場合は、実測値の測定を追加することを推奨する。
7）術後 8 週以内に化学療法による治療の開始が見込める症例
8）本試験の被験者となることについて本人に同意説明文書を用いて説明し、文書にて同意が得られている症例。

0.4.3. 除外基準

以下の項目のいずれかに該当する症例は除外する。
1）S-1 投与禁忌の症例
①S-1 の成分に対し重篤な過敏症の既往歴のある症例
②重篤な骨髄抑制または腎障害または肝障害のある症例
③他のフッ化ピリミジン系抗悪性腫瘍剤を投与中の症例
④フルシトシンを投与中の症例
2）重篤な薬剤アレルギーの既往を有する症例
3）6 ヶ月以内に発症した、心筋梗塞の既往を有する症例
4）胸部 X 線写真で明らかな間質性肺炎、肺線維症の症例
5）ワルファリンまたはダビガトランの定期的な内服を要する症例
6）心電図（心エコーも要に応じて）上、臨床的に問題となる変化を有する症例
7）臨床上問題となる心疾患を有する症例、精神疾患等を有する症例、感染症を有する症例、重篤な合併症を有する症例
8）コントロール困難な糖尿病を合併している症例
9）腸管麻痺、腸閉塞を有する症例
10）水様性下痢のある症例
11）活動性の重複癌*を有する症例
12）HBs 抗原陽性症例
13）その他、試験責任医師が不適当と判断した症例
*重複癌：同時多発性および無病期間が 5 年以内の異時性重複癌であり、局所治療により治癒と判断される Carcinoma in situ（上皮内癌）もしくは粘膜内癌相当の病変は活動性重複癌に含めないこととする。

0.5. 予定登録数と研究期間

予定登録患者数：100 例

登録期間：4 年（2012 年 5 月～2016 年 4 月）

主要評価項目に関する追跡期間：登録終了後 6 ヶ月

研究期間：2012 年 4 月～2022 年 6 月

0.6. 問い合わせ先

研究代表者（瀬戸内肺癌研究会）

豊岡伸一

岡山大学大学院医歯薬学総合研究科

呼吸器・乳腺内分泌外科学

研究事務局

諏澤憲、山本寛斉、岡﨑幹生

岡山大学病院　呼吸器外科　　〒700-8558　岡山市北区鹿田町 2-5-1

Tel: 086-235-7265、Fax: 086-235-7269

目　次

0. 概要 2
1. 目的 8
2. 背景 8
　2.1. 本邦における補助化学療法に対するこれまでの知見 8
　2.2. S-1 について 8
　2.3. S-1 の隔日投与法について 8
3. 本試験で用いる基準・定義 10
　3.1. TNM 腫瘍分類 10
　3.2. 病期分類（病理病期） 11
　3.3. 治療に関する用語（延期・休薬・休止・中止）の定義 11
4. 患者選択基準 12
　4.1. 選択基準 12
　4.2. 除外基準 12
5. 登録 13
　5.1. 登録の手順 13
　5.2. 登録に際しての注意事項 13
　5.3. ランダム割付と割付調整因子 13
6. プロトコール治療 14
　6.1. 概要 14
　6.2. A 群：S-1 隔日投与法 14
　6.3. B 群：S-1 2 週投与 1 週休薬法 18
7. プロトコル治療中止、終了基準、後治療 21
　7.1. プロトコル治療中止基準 21
　7.2. 試験を中止した被験者に対するフォローアップ 21
　7.3. プロトコル治療終了の定義 21
　7.4. 後治療 21
8. 併用薬および併用療法、支持療法 21
9. 予期される薬物有害事象 23
　9.1. 薬剤情報 23
　9.2. 有害事象の定義 23
　9.3. 有害事象/有害反応の評価 23
　9.4. 予期される有害反応 23
　9.5. 試験薬との因果関係 23
10. 有害事象の報告 23
　10.1. 急送報告義務のある有害事象 23
　10.2. 通常報告義務のある有害事象 23
　10.3. 施設研究責任者の報告義務と報告手順 24
　10.4. 研究代表者/研究事務局の責務 24
11. 観察・検査項目および実施時期 24
　11.1. 治療期間の定義 24
　11.2. 手術前の評価項目 24
　11.3. 治療開始前（登録時）の評価項目（登録前 2 週間以内） 24
　11.4. 治療期間中の評価項目 25
　11.5. 薬剤投与中止/終了後 25
　11.6. 再発の兆候を認めた場合 25
12. 評価項目の評価方法 26
　12.1. 評価項目 26
　12.2. 評価方法 26
13. 目標症例と試験実施期間 27
　13.1. 症例集積期間/追跡期間 27
　13.2. 解析の対象となる被験者の選択 27

13.3. 症例数の設定根拠 27
13.4. 試験終了後の結果による標準的治療法(S-1 投与法)の Decision criteria 28
14.試験の中止 28
15.データの収集および保存 28
16. 倫理的事項 28
16.1. 被験者の保護 29
16.2. 同意の取得 29
16.3. 説明事項 29
16.4. プライバシーの保護と被験者識別 29
16.5. プロトコルの遵守 29
16.6. 施設の試験審査委員会（IRB）または倫理審査委員会の承認 29
16.7. プロトコルの内容変更について 29
16.8. 症例報告用紙（CRF）の内容変更について 30
16.9. デ-タの二次利用について 30
17. モニタリングと監査 31
17.1. モニタリングの項目 31
17.2. プロトコル逸脱 31
18. 費用と補償 31
19. 研究資金および利益の衝突 31
20. 研究に関する情報公開の方法（研究計画の登録および研究結果の発表） 31
21. QOL 解析... 32
21.1. 必要な試料 32
21.2. 調査方法 32
21.3. 試料送付 32
21.4. 試料解析 32
21.5. 試料の解析後処理 32
22. 疾病等報告 32
23. 研究組織 33
23.1. 研究代表者（瀬戸内肺癌研究会） 33
23.2. 研究事務局 33
23.3. 参加予定施設 33
23.4. 効果・安全性評価委員会（順不同、敬称略） 33
23.5. 統計解析・登録センター 33
23.6. データセンター 33
24.参考文献 34

1. 目的

病理病期 IA(T1bN0M0)/IB/II/IIIA 期の高齢者非小細胞肺癌完全切除例に対する術後化学療法としてS-1 隔日投与の認容性・有効性を検討する。

Primary endpoint: 投与完遂率（Feasibility）

Secondary endpoint: 有害事象発生割合と程度、無再発生存期間(RFS)、全生存期間(OS)、QOL

2. 背景

2.1. 本邦における補助化学療法に対するこれまでの知見

I-IIIA 期非小細胞肺癌に対する第一選択は従来外科的切除とされてきたが、最近欧米で実施された 3 つの大規模比較試験 [1)2)3)] において、プラチナベースの化学療法により生存割合が有意に改善することが示された。一方、本邦では術後 UFT 単剤療法に関するメタ解析が行われ、UFT の有効性が確認された [4)]。これを受けて 2004 年以降、外科手術により完全切除された病理病期 IA(T1ｂN0M0)、IB～IIIA 期は手術に術後化学療法を加えることが標準治療と考えられている。但、高齢患者に焦点を当てると、他の健康障害を併発していることが多く、積極的に抗癌治療を行ってよいと判断できる十分なエビデンスは得られていない。つまり、高齢患者への術後化学療法については、化学療法によって生じる副作用を上回る効果が期待できるのか結論は出ておらず、治療成績の向上のための治療法開発が求められている。

2.2. S-1 について

S-1（テガフール・ギメラシル・オテラシルカリウム配合剤）は本邦で開発された経口フッ化ピリミジン系抗癌剤であり、胃癌に対して有効な薬剤として 1999 年 1 月に認可された。本剤は 5-FU のプロドラッグであるテガフールにギメラシル（5-FU 分解系の律速酵素の可逆的拮抗剤）およびオテラシルカリウム（消化管毒性の抑制を目的とした 5-FU のリン酸化酵素の可逆的阻害剤）を配合することにより、血中 5-FU 濃度を高めて抗腫瘍効果を増強し、また、付随して増大する消化管毒性の軽減をねらった薬剤である [5) 6)]。

国内での未治療進行非小細胞肺癌における S-1 単剤(80mg/m2/日/相当)の 4 週投薬 2 週休薬法の奏効率は 22.0％であり、テガフールとウラシルの合剤である UFT 単剤の奏効率 6％を大きく上回り、従来の新規抗癌剤(タキサン等)と比較しても遜色はない [7)]。主な有害事象は消化器毒性であり、食思不振 10%、下痢 9%などであった。

また、非小細胞肺癌完全切除例（StageⅠB～ⅢA）を対象とした S-1 単剤（80mg/m2/日、2 週投薬 1 週休薬、術後 1 年間）の２相試験が実施され、１年 DFS は 83.7%（I 期：95%、II 期：72.7%、III 期：75.0%)と有望な結果であった [8)]。頭頸部癌切除症例では、S-1 単剤(80mg/m2/日/相当)の補助化学療法のスケジュールとして、従来の投与法である 4 週投薬 2 週休薬を 2 週投薬 1 週休薬の投与法と無作為化比較したところ、後者の方が内服コンプライアンスは良好であり(6 ヶ月間: 54% vs. 69%)、下痢の出現率も低かった(10% vs. 28%) [9)]。以上から後者の投与法がより安全性・認容性に勝ることが示唆された。

2.3. S-1 の隔日投与法について

1960 年初期、Lipkin [10)]、Clarkson [11)]、Cronkite [12)] らは、宿主の正常細胞（消化管粘膜細胞、骨髄細胞）と癌細胞との細胞周期に大きな違いがあることを報告している。

そこで Shirasaka らは、この違いを利用して、時間依存性の高い代謝拮抗剤である 5-FU に関して、先ず、宿主の正常細胞に着目し、消化管毒性および骨髄毒性を軽くすることを考え、次に癌細胞に対して殺細胞効果が落ちない方法を生物学的に見いだした。

宿主の正常細胞の細胞周期は約半日から 1 日で、その殆どの時間が 5-FU が作用する S 期であり、それは約 12 時間であり、一日（24 時間）の 5-FU の非曝露（休薬）でかなりの正常細胞は 5-FU の作用を受けない。一方、癌細胞の細胞周期は 4-5 日と正常細胞より長く、さらに S 期は 24 時間以上と長いため、休薬を挟んで 24 時間毎に 5-FU を繰り返し作用させることによって、癌細胞に対する殺細胞効果は減弱しないと考えた。また、Shirasaka らは in vitro の系において、5-FU の殺細胞活性(IC_{50})は、6 時間または 12 時間毎の on/off にしても繰り返すことで減弱しないことを見い出していることから S-1 の隔日投与法を提案した。

隔日投与法は基礎および臨床での成績から有効性が明らかになりつつあり [13) 14)]、自治医大・消化器外科のレトロスペクティブなデータでは、再発、術後の胃癌 92 症例に対し、S-1 の基準投与法である、4 週間連日投与 2 週間休薬治療法で開始し、Gr1 以上の非血液毒性が 72.8%（悪心・嘔吐； 16.3%、食欲

不振； 15.2%、下痢； 21.7%、全身倦怠感； 19.6%）が認められ、患者の申し出により継続投与が困難となった 72 症例に対し、約 1 週間の休薬の後、隔日投与に変更し治療を継続した。その結果、Gr 1 以上の非血液毒性が食欲不振 0%、悪心・嘔吐 2 例（2.8%）および下痢 2 例（2.8%）と著明に減少し、そのため平均治療継続期間は連日投与の 47 日に対し隔日投与は 272 日と大幅に延長した。またこの際の TTP は 170 日、MST は 11 ヵ月であり、評価可能症例における病勢制御率は 53%（31/58）であり [12]、良好な成績であった。因みに隔日投与法における薬物動態からも、5-FU の血中濃度の Cmax は連日投与のそれと同等であることも認められている [13]。

前向きの臨床試験としては、現在膵癌と胃癌で報告がある [14)15)]。膵癌においては、第 49 回日本癌治療学会にて、和歌山県医大の山上らが切除不能進行膵癌患者に S-1 隔日投与を検討した第Ⅱ相臨床試験の安全性データを報告している。結果としては、消化器関連の副作用は G1：4.2%、G2：6.2%、G3≧：0%、血液毒性は G1:6.2%、G2:16.7%、G3:4.2%と Grade3 以上の副作用が低い傾向であり、今後のフォローアップ後の全生存期間の結果が期待される [14]。胃癌では、山陰胃癌化学療法研究会の辻谷らが 2012 年での ASCO GI にて S-1 の 4 週間投与 2 週間休薬群および隔日投与群を比較検討した第Ⅱ相臨床試験結果を公表しており、コンプライアンス（72.2 % vs 91.8 %）、1 年無再発生存率（82.9% vs 91.7%）と、隔日投与法が胃癌術後化学療法の治療オプションの 1 つと考察している [15]。只、実地臨床として S-1 の隔日投与による治療を他の癌腫で実施するためには臨床試験のデータが不足しており、早急なデータ構築が求められている。

以上の事から、本研究では高齢患者に合わせたより良い術後補助化学療法を構築するため、S-1 隔日投与および先述の連日投与法（2 週間投与 1 週間休薬）の投与継続性を比較検討することとした。

3.本試験で用いる基準・定義

本試験においては、日本肺癌学会編「臨床病理　肺癌取り扱い規約」改訂第7版の定義を用いる。

3.1. TNM腫瘍分類

T-原発腫瘍

T0 原発腫瘍を認めない

Tis 上皮内癌 (carcinoma in situ)

T1：腫瘍最大径≦3 cm，肺か臓側胸膜に覆われている，葉気管支より中枢への浸潤が気管支鏡上なし（すなわち主気管支に及んでいない）

T1a 腫瘍最大径≦2 cm

T1b 腫瘍最大径＞2 cm でかつ≦3 cm

T2：腫瘍最大径＞3 cm でかつ≦7 cm，または腫瘍最大径≦3 cm でも以下のいずれかであるもの（T2a）

- 主気管支に及ぶが気管分岐部より≧2 cm 離れている
- 臓側胸膜に浸潤
- 肺門まで連続する無気肺か閉塞性肺炎があるが一側肺全体には及んでいない

T2a 腫瘍最大径＞3 cm でかつ≦5 cm，あるいは≦3 cm で胸膜浸潤有り（PL1，PL2，葉間の場合はPL3）

T2b 腫瘍最大径＞5 cm でかつ≦7 cm

T3：最大径＞7 cm の腫瘍；胸壁（superior sulcus tumor を含む），横隔膜，横隔神経，縦隔胸膜，心嚢のいずれかに直接浸潤；分岐部より2 cm 未満の主気管支に及ぶが分岐部には及ばない；一側肺に及ぶ無気肺や閉塞性肺炎；同一葉内の不連続な副腫瘍結節

T4：大きさを問わず縦隔，心，大血管，気管，反回神経，食道，椎体，気管分岐部への浸潤，あるいは同側の異なった肺葉内の副腫瘍結節

N-所属リンパ節

N0 所属リンパ節転移なし

N1 同側の気管支周囲かつ または同側肺門,肺内リンパ節への転移で原発腫瘍の直接浸潤を含める

N2 同側縦隔かつ または気管分岐部リンパ節への転移

N3 対側縦隔，対側肺門，同側あるいは対側の前斜角筋，鎖骨上窩リンパ節への転移

M-遠隔転移

M0 遠隔転移なし

M1 遠隔転移がある

M1a 対側肺内の副腫瘍結節，胸膜結節，悪性胸水（同側，対側），悪性心嚢水

M1b 他臓器への遠隔転移がある

M1 は転移臓器によって以下のように記載する

肺 PUL、骨髄 MAR、骨 OSS、胸膜 PLE、肝 HEP、腹膜 PER

脳 BRA、副腎 ADR、リンパ節 LYM、皮膚 SKI、その他 OTH

pTNM 分類・病理学的分類

pT，pN，pM 各分類は T，N，M 各分類に準ずる。

pN0と判定するには肺門と縦隔リンパ節摘出標本が通常6 個以上組織学的に検索されていることが望ましい.

3.2.　病期分類（病理病期）

ステージ	T	N	M
0期	上皮内がん	N0	M0
IA期	T1a または T1b	N0	M0
IB期	T2a	N0	M0
IIA期	T1a または T1b	N1	M0
	T2a	N1	M0
	T2b	N0	M0
IIB期	T2b	N1	M0
	T3	N0	M0
IIIA期	T1a または T1b	N2	M0
	T2a	N2	M0
	T2b	N2	M0
	T3	N2	M0
	T3	N1	M0
	T4	N0	M0
	T4	N1	M0
IIIB期	T は関係なし	N3	M0
	T4	N2	M0
IV期	T は関係なし	N は関係なし	M1a または M1b

3.3.　治療に関する用語（延期・休薬・休止・中止）の定義

延期： S-1 の投与を規定より遅延させること。
休薬： プロトコルで規定したスケジュール通りに薬剤投与を停止すること。
休止： 有害事象等発現、その他の理由等によってスケジュール予定外で薬剤投与を停止することで、投与再開の可能性があるもの。
中止： プロトコル治療の途中終了で、再開の可能性がないもの。

4. 患者選択基準

4.1. 選択基準

1）病理学的に非小細胞肺癌と診断された病理病期 IA(T1bN0M0)/IB/II/IIIA 期の症例。ただしカルチノイド、粘表皮癌、腺様嚢胞癌などの低悪性腫瘍を除く。
2）肺葉切除以上の外科切除が行われた症例で、リンパ節郭清が行われ(原則として ND2a 以上)、病理学的に完全切除を確認できた症例。
3）原発巣に対して手術以外の前治療がない症例。
4）年齢が登録時に 75 歳以上（満年齢）の症例。
5）ECOG の Performance Status（PS）が 0～1 の症例
6）重篤な術後合併症がなく、検査値が以下の基準を満たすこと。
- ・白血球数：3,000～12,000/mm^3 未満
- ・血小板数：10×10^4/mm^3 以上
- ・血色素量：9.0g/dl 以上
- ・総ﾋﾞﾘﾙﾋﾞﾝ：1.5mg/dl 以下
- ・AST（GOT）、ALT（GPT）：100(IU/L)未満
- ・PaO_2：60mmHg 以上（もしくは SpO_2 90%以上でも可）
- ・血清ｸﾚｱﾁﾆﾝ：1.2mg/dl 未満
- ・クレアチニンクリアランス（CCr）値※：40 mL/min 以上

※登録に当たっては、24 時間蓄尿によるクレアチニンクリアランス実測値、または Cockcroft-Gault 式の推定値を用いる。ただし実測値を優先する。推定値で適応下限であった場合は、実測値の測定を追加することを推奨する。

7）術後 8 週以内に化学療法による治療の開始が見込める症例
8）本試験の被験者となることについて本人に同意説明文書を用いて説明し、文書にて同意が得られている症例。

4.2. 除外基準

以下の項目のいずれかに該当する症例は除外する。

1）S-1 投与禁忌の症例
①S-1 の成分に対し重篤な過敏症の既往歴のある症例
②重篤な骨髄抑制または腎障害または肝障害のある症例
③他のフッ化ピリミジン系抗悪性腫瘍剤を投与中の症例
④フルシトシンを投与中の症例
2）重篤な薬剤アレルギーの既往を有する症例
3）6 ヶ月以内に発症した、心筋梗塞の既往を有する症例
4）胸部 X 線写真で明らかな間質性肺炎、肺線維症の症例
5）ワルファリンまたはダビガトランの定期的な内服を要する症例
6）心電図（心エコーも要に応じて）上、臨床的に問題となる変化を有する症例
7）臨床上問題となる心疾患を有する症例、精神疾患等を有する症例、感染症を有する症例、重篤な合併症を有する症例
8）コントロール困難な糖尿病を合併している症例
9）腸管麻痺、腸閉塞を有する症例
10）水様性下痢のある症例
11）活動性の重複癌*を有する症例
12）HBs 抗原陽性症例
13)その他、試験責任医師が不適当と判断した症例

*重複癌：同時多発性および無病期間が 5 年以内の異時性重複癌であり、局所治療により治癒と判断される Carcinoma in situ（上皮内癌）もしくは粘膜内癌相当の病変は活動性重複癌に含めないこととする。

5. 登録

5.1. 登録の手順

研究責任/研究分担医師は、対象患者が適格基準を全て満たし、除外基準に抵触しないことを確認し、「症例登録用紙」に必要事項をすべて記入のうえ、登録センターへ症例登録用紙を FAX 送付する。登録センターでは適格性を確認した後、症例登録番号を記入した、症例登録結果通知書を担当医宛てに FAX にて返信する。登録確認通知書の指示に従い術後 8 週以内に開始すること。

【登録事務局】

登録事務局：岡山大学病院呼吸器外科
枝園和彦、山本寛斉、宗淳一
Fax 番号: 086-235-7269（呼吸器外科専用）

5.2. 登録に際しての注意事項

(1) プロトコル治療開始後の事後登録は許容されない。
(2) 症例登録用紙の記載が不十分な時は、すべて満たされるまでは登録は受け付けられない。
(3) 登録センターで適格性が確認された後に、症例登録番号が発行される。
(4) 登録されると症例登録番号が記入された症例登録結果通知書が登録センターより FAX にて担当医に返信されるので適切に保管すること。
(5) 一度登録された症例は登録取り消し（データベースから抹消）はなされない。重複登録の場合は初回の登録情報（症例登録番号）を採用する。
(6) 誤登録・重複登録が判明した際には速やかに登録センターに連絡すること。
(7) 登録は原則として治療開始予定日の前日までに症例登録用紙を FAX 送付すること。

5.3. ランダム割付と割付調整因子

症例登録にあたって各症例の治療法は九州大学大学院医学研究院予防医学分野松尾恵太郎医師においてランダムに割り付けられる。ランダム割付には病理病期（IA(T1bN0M0)/ⅠB 期 or Ⅱ期 or ⅢA 期)、組織型（非扁平上皮癌 or 扁平上皮癌（確定困難な場合は非扁平上皮癌とすること))、EGFR 遺伝子変異(変異あり or 変異なし又は不明)、施設の 4 因子を割付調整因子とした最小化法を用いる。

6. プロトコール治療

6.1. 概要

本試験にて実施するS-1隔日投与法およびS-1 2週投与1週休薬法をプロトコル治療と定義し、原則として登録後1週間以内、かつ、術後8週間以内にプロトコル治療を開始する。術後8週間を超えた場合は、データセンターへ連絡をし、プロトコル治療開始可能か否かを確認し、可能であれば開始する。開始後は、8週間以内に開始できなかった理由を「試験開始・治療前中止報告書」のコメント欄へ記載する。なお、TS-1は、ティーエスワン配合カプセルT20,T25、ティーエスワン配合顆粒T20,T25、ティーエスワン配合OD錠T20,T25（大鵬薬品工業株式会社）とする。

6.2. A群：S-1隔日投与法

6.2.1. ：A群（S-1隔日投与法）の治療スケジュール

・治療は術後8週間以内に開始する。

<u>投与スケジュール</u>

・S-1の初回投与は月、水、金、日のいずれかの曜日（規定曜日）で開始する。S-1を投与開始した日をもって、Day1とする。
・S-1を朝食後に内服できない場合は、夕食後から内服を開始して良い。夕食後から開始した場合も初回投与開始日とし、次回の投与は規定曜日に行う。
（例：金曜日の夕から内服した場合、次の投与は日曜日の朝）
・S-1投与は月、水、金、日に行うこととし、治療中止基準のいずれかに該当するまで投与を継続する。
<u>S-1を投与する曜日はずらさない。</u>
・ 投与最終日から28日を超えても投与が開始できない場合は本試験治療を中止する（最終投与翌日を1日目とする。最終投与日の4週間後の同じ曜日の投与は可とする）。

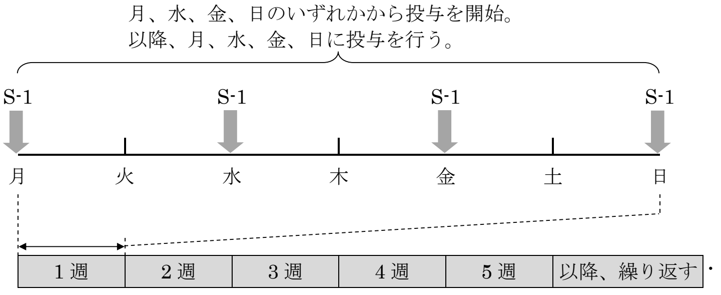

<u>投与開始量・投与開始方法</u>

S-1は体表面積に合わせて1日量を表1.「S-1初回基準量と一段階減量用量」から算出し、均等に2分割して、朝食後および夕食後に経口投与する。症例登録時のクレアチニンクリアランス（CCr）が40mL/min以上60mL/min未満であれば、表1「S-1初回基準量と一段階減量用量」に従いS-1を初回基準量から一段階減量して投与開始する。初回化学療法投与前に投与量を計算した体重をベースラインとし、そこから10%以上減少した場合は、再計算を行い、同時にそれ以後のベースライン体重は再計算時の体重におきかえて考える。

表1.　S-1初回基準量と一段階減量用量

体表面積	初回基準量（FT相当量）<br>（CCr≧60）	減量レベル　−1<br>（40≦CCr＜60）
1.25m^2未満	80 mg/日	50 mg /日
1.25m^2以上〜1.5m^2未満	100 mg/日	80 mg /日
1.5m^2以上	120 mg/日	100 mg /日

6.2.2. 投与開始基準（A 群）

初回投与開始時には投与開始日の前日または当日に 4.1.選択基準 6)を満たしていることを確認の上、投与を開始する。

基準に満たない場合には投与開始を延期し、該当する項目が 4.1.選択基準 6)を満たしたことを確認してから初回投与を開始する。

6.2.3. 投与継続基準（A 群）

初回投与以降、表 2 の「投与継続基準」を満たしていない場合は S-1 の投与を休止する。

初回投与開始 21 日以内に表 2 の「投与継続基準」を満たしていないことを確認した際は、S-1 を休止し、次回投与から表 5. 減量用量レベルに従って S-1 の減量を考慮する。

表 2. 「投与継続基準」

項目	投与継続基準
白血球数	2,000 /mm³ 以上　12,000 /mm³ 以下
好中球数	1,000 /mm³ 以上
血小板数	75,000 /mm³ 以上
総ビリルビン	1.5 mg/dL 以下
AST および ALT	100 IU/L 以下
クレアチニン	1.2 mg/dL 未満
肺臓炎	Grade 0
感染	感染を疑う発熱なし
PS	0～1
下痢、口内炎	Grade 1 以下
その他の非血液毒性	Grade 2 以下<br>（但、主治医の判断で投与可能とする場合もある）
その他	上記に該当しない有害事象の発現で担当医師が必要と判断した場合には休薬できる

6.2.4. 投与再開基準（A 群）

休止後、再開する場合は表 3 の「投与再開基準」を満たしていることを確認する。ただし、有害事象が表 4 の「減量基準」に該当した場合は、再開時から減量すること。また、表 2「投与継続基準」以外の有害事象により休止した場合は、原因となった有害事象のグレードが 1 段階以上改善し、投与が可能と担当医師が判断していること。

28 日以上経過しても S-1 治療を再開できない場合は、プロトコル治療を中止する。

表 3「投与再開基準」

項目	投与再開基準
白血球数	3,000/mm³ 以上 12,000 /mm³ 以下
好中球数	1,500/mm³ 以上
血小板数	100,000 /mm³ 以上
総ビリルビン	1.5 mg/dL 以下
AST および ALT	100 IU/L 以下
クレアチニン	1.2 mg/dL 未満
肺臓炎	Grade 0
感染	感染を疑う発熱なし
PS	0～1
下痢、口内炎	Grade 1 以下
その他の自他覚所見<br>および一般臨床所見	Grade 2 以下<br>（但、主治医の判断で投与可能とする場合もある）

6.2.5. 減量基準（A 群）

表 4. に定める減量基準に該当する有害事象を認めた症例に対して、投与を再開する場合は、表 5.「減量用量レベル」に従って 1 段階ずつ減量を行う。ただし、S-1 の最低投与用量は 50 mg/日とし、減量した症例の再増量は行わない。表 4 以外の有害事象に関しても、担当医師の判断により S-1 の減量を行うことができるが、その際も表 5.の減量用量レベルに従う。

表 4「減量基準」

項目	減量基準
白血球数	1,000 /mm^3 未満（Grade4）
好中球数	500 /mm^3 未満（Grade4）
	発熱性好中球減少（Grade3 以上）
血小板数	25,000 /mm^3 未満（Grade 4）
	血小板輸血を行った Grade 3 の血小板減少
クレアチニン	1.2 mg/dL 以上
総ビリルビン	2.0 mg/dL 以上
非血液学的毒性(低 Na は除く)	Grade 3 以上

※Grade 4 の非血液学的毒性（肺臓炎は Grade 1 以上）はプロトコル治療中止とする。

表 5. 減量用量レベル(S-1 の減量方法)

体表面積	治療開始時（FT 相当量）	減量レベル			
		−1	−2	−3	−4
1.25m^2 未満	80 mg/日	50 mg /日	治療中止	—	—
1.25m^2 以上～1.5m^2 未満	100 mg/日	80 mg /日	50 mg /日	治療中止	—
1.5m^2 以上	120 mg/日	100 mg /日	80mg/日	50 mg /日	治療中止

6.2.6. 増量基準（A 群）

・投与開始 43 日目以降、休薬/減量となる有害事象が認められず、安全性に問題がないと担当医師が判断した場合は、表 6. 増量用量レベルに従い初回基準量から 1 段階に限って S-1 を増量することができる。
  - ・登録時 CCr 値（40≦CCr＜60）により、初回投与から減量している症例は増量しない。
  - ・減量した症例の再増量は行わない。
  - ・増量した症例の減量は表 7. 増量症例の減量用量レベルに従う。

表 6. 増量用量レベル

S-1 の増量方法		
体表面積	初回基準量（FT 相当量）	増量レベル ＋1
1.25m^2 未満	80 mg/日	100 mg/日
1.25m^2 以上～1.5m^2 未満	100 mg/日	120 mg/日
1.5m^2 以上	120 mg/日	150 mg/日

表 7. 増量症例の減量用量レベル

S−1 の増量症例の減量方法				
体表面積	増量後の投与量（FT 相当量）	減量レベル		
		−1	−2	−3
1.25m^2 未満	100 mg/日	80 mg/日	50 mg /日	治療中止
1.25m^2 以上～1.5m^2 未満	120 mg/日	100 mg/日	80 mg/日	50 mg/日
1.5m^2 以上	150 mg/日	120 mg/日	100 mg/日	80 mg/日

6.2.7. プロトコール治療終了の基準（A 群）

肺癌術後補助化学療法としての S-1 初回投与日から起算して 1 年経過した時点で、プロトコル治療を終了とする。

6.2.8. 投与シミュレーション（A 群）

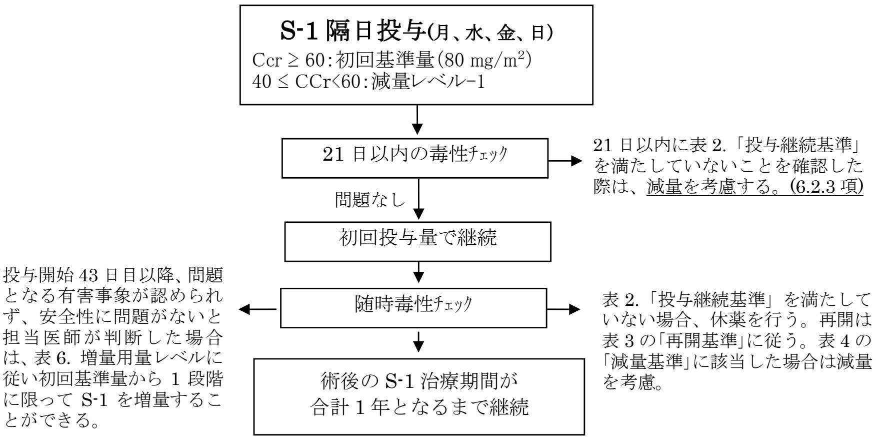

6.3.　B群：S-1　2週投与1週休薬法
6.3.1.　B群（S-1 2週投与1週休薬法）の治療スケジュール
<u>投与サイクル</u>：第1日目～14日間連日経口内服（第1日目の夕食後～第15日朝食後）
その後7日間休薬、これを3週間ごとに1コースとして、繰り返す。
<u>投与終了日</u>：プロトコル治療開始日からの治療期間が計12ヵ月となるまで行う。プロトコル治療開始日から12ヵ月後の同じ暦日以降は新たなコースに入らないこととする。最終コースはday14相当日まで投与を行うこと。

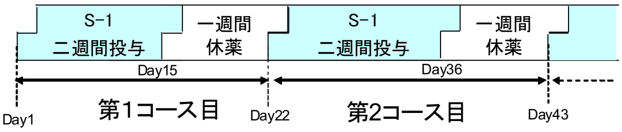

<u>投与開始量・投与開始方法</u>

S-1は体表面積に合わせて1日量を表8.「S-1初回基準量と一段階減量用量」から算出し、均等に2分割して、朝食後および夕食後に経口投与する。症例登録時のクレアチニンクリアランス（CCr）が40mL/min以上60mL/min未満であれば、表8「S-1初回基準量と一段階減量用量」に従いS-1を初回基準量から一段階減量して投与開始する。初回化学療法投与前に投与量を計算した体重をベースラインとし、そこから10%以上減少した場合は、再計算を行い、同時にそれ以後のベースライン体重は再計算時の体重におきかえて考える。

表8.　S-1初回基準量と一段階減量用量

体表面積	初回基準量（FT相当量）（CCr≧60）	減量レベル　−1（40≦CCr＜60）
1.25m^2未満	80 mg/日	50 mg /日
1.25m^2以上～1.5m^2未満	100 mg/日	80 mg /日
1.5m^2以上	120 mg/日	100 mg /日

6.3.2.：B群（S-1　2週投与1週休薬法）のコース開始基準
次コース開始は、投与開始予定の前日又は当日に、以下の「表9　コース開始基準」全ての基準を満たすことを確認した上で決定する。基準を1つでも満たしていない場合は投与延期し、其々回復次第、投与開始する。
ただし、2コース目以降の開始に際しては、**次コース開始予定日より21日を越えても本基準を満たさない場合は、当該症例の試験を中止**する(下記図参照)。
なお、コース開始延期の場合、延期後の開始日をそのコースのday1とする。
また、治療経過中に再発が見られた場合は、プロトコル治療をその時点で中止すること。

表9　コース開始基準（B群）

項目		程度
血液毒性	白血球数	3,000/mm^3以上
	好中球数	1,500/mm^3以上
	血小板数	100,000/mm^3以上
非血液毒性	総ビリルビン	1.5mg/dL以下
	ASTおよびALT	100 IU/L以下
	ｸﾚｱﾁﾆﾝ	1.2mg/dL未満
	肺臓炎	Grade 0
	感染	感染を伴う発熱がない
	PS	0～1
	その他の自他覚所見および一般臨床所見	Grade 2以下（但、主治医の判断で投与可能とする場合もある）

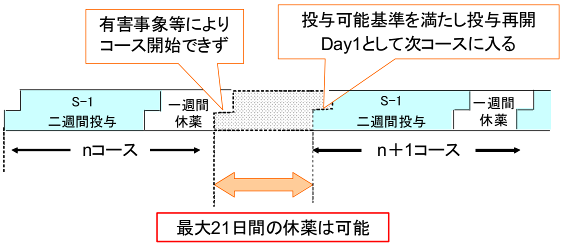

6.3.3.：　コース内における休薬、再開の基準（B 群）

S-1 投与中に、表 10 の基準に該当する有害事象が発現した場合は、S-1 を休薬する。休薬した場合は、再開基準まで回復を待って投与を再開する。Day15 朝食後までに同有害事象が回復しなかった場合は、当該コースの S-1 投与はそれ以上行わない。再開後の服用は day15 朝食後までとする。Grade 4 の非血液学的毒性（肺臓炎は Grade　1 以上）はプロトコル治療中止とする。

表 10　　S-1 単独治療の休薬判断基準

項目		休薬基準	再開基準
血液毒性	白血球数	2,000/mm³ 未満	2,000/mm³ 以上
	好中球数	1,000/mm³ 未満	1,000/mm³ 以上
	血小板数	75,000/mm³ 未満	75,000/mm³ 以上
非血液毒性	総ビリルビン	1.5mg/dL を超える	1.5mg/dL 以下
	AST および ALT	100　IU/L を超える	100　IU/L　以下
	ｸﾘｱﾁﾆﾝ	1.2mg/dL 以上	1.2mg/dL 未満
	感染	感染を疑う発熱	感染を疑う発熱なし
	PS	2 以上	0～1
	下痢・口内炎	Grade　2 以上	Grade　1 以下
	その他の非血液毒性	Grade　3 以上	Grade　2 以下<br>（但、主治医の判断で投与可能とする場合もある）
	その他	上記に該当しない有害事象の発現で担当医師が必要と判断した場合には休薬できる	休薬の原因となった有害事象が軽快

※Grade 4 の非血液学的毒性（肺臓炎は Grade　1 以上）はプロトコル治療中止とする。

6.3.4.：減量基準（B 群）

表 11　に定める減量基準に該当する有害事象を認めた症例に対して、投与を再開する場合は、表 12.「減量用量レベル」に従って 1 段階ずつ減量を行う。ただし、S-1 の最低投与用量は 50　mg/日とし、減量した症例の再増量は行わない。表 11 以外の有害事象に関しても、担当医師の判断により S-1 の減量を行うことができるが、その際も表 12.の減量用量レベルに従う。

表 11　減量基準

項目	減量基準
白血球数	1,000 /mm³ 未満（Grade4）
好中球数	500 /mm³ 未満（Grade4）
	発熱性好中球減少（Grade3 以上）
血小板数	25,000 /mm³ 未満（Grade 4）
	血小板輸血を行った Grade 3 の血小板減少
クレアチニン	1.2 mg/dL 以上
総ビリルビン	2.0 mg/dL 以上
非血液学的毒性(低 Na は除く)	Grade 3　以上

※Grade 4 の非血液学的毒性（肺臓炎は Grade　1 以上）はプロトコル治療中止とする。

表 12. 減量用量レベル(S-1 の減量方法)

体表面積	治療開始時（FT 相当量）	減量レベル			
		－1	－2	－3	－4
1.25m² 未満	80 mg/日	50 mg /日	治療中止	—	—
1.25m² 以上～1.5m² 未満	100 mg/日	80 mg /日	50 mg /日	治療中止	—
1.5m² 以上	120 mg/日	100 mg /日	80mg/日	50 mg /日	治療中止

6.3.3. 増量について（B 群）

・2 コース目以降、休薬/減量となる有害事象が認められず、安全性に問題がないと担当医師が判断した場合は､表 13. 増量用量レベルに従い初回基準量から 1 段階に限って S-1 を増量することができる。

・登録時 CCr 値（40≦CCr＜60）により、初回投与から減量している症例は増量しない。

・減量した症例の再増量は行わない。

・増量した症例の減量は表 14. 増量症例の減量用量レベルに従う。

表 13. 増量用量レベル

S-1 の増量方法		
体表面積	初回基準量（FT 相当量）	増量レベル
		＋1
1.25m² 未満	80 mg/日	100 mg/日
1.25m² 以上～1.5m² 未満	100 mg/日	120 mg/日
1.5m² 以上	120 mg/日	150 mg/日

表 14. 増量症例の減量用量レベル

S-1 の増量症例の減量方法				
体表面積	増量後の投与量（FT 相当量）	減量レベル		
		－1	－2	－3
1.25m² 未満	100 mg/日	80 mg/日	50 mg /日	治療中止
1.25m² 以上～1.5m² 未満	120 mg/日	100 mg/日	80 mg/日	50 mg/日
1.5m² 以上	150 mg/日	120 mg/日	100 mg/日	80 mg/日

7. プロトコル治療中止、終了基準、後治療

7.1. プロトコル治療中止基準

以下の基準に該当した場合、担当医師はプロトコル治療を中止する。

1) S-1 治療の最終投与日から 28 日（B 群でいう次コース開始予定日から 21 日に当たる）が経過してもプロトコル治療を開始できなかった場合（この場合、S-1 治療の最終投与日の翌日を 1 日目とする。同じ曜日の投与開始は許容する）
2) 28 日以上の休薬を要する場合（この場合、S-1 最終投与日の翌日を 1 日目とする。最終投与日の 4 週間後の同じ曜日の再開は許容する）
3) S-1 を最低段階まで減量しても各々の減量基準に該当する有害事象が発現した場合
   または、Grade 4 の非血液学的毒性（肺臓炎は Grade　1 以上）が発現した場合。
4) 投与継続が困難な有害事象が発現した場合
5) 担当医師が再発と判断した場合
6) 患者からの中止の申し出があった場合
7) 対象から除外すべき事項が登録後に判明した場合
8) その他、担当医師が中止を必要と認めた場合

7.2. 試験を中止した被験者に対するフォローアップ

治療中止基準に該当した場合は、プロトコル治療を中止し、その時期、理由、経過を「症例報告書」に記録し、中止時点までの評価を行う。なお、中止例においても有害事象の追跡調査を S-1 最終投与後 4 週間（28 日目）まで、あるいは本剤に起因すると考えられる有害事象が消失、軽快、安定化あるいは臨床検査値が正常値に回復、安定化するまで行う。

7.3. プロトコル治療終了の定義

試験期間中に 7.1 項のプロトコル治療中止基準に該当せず、プロトコル治療開始日から A 群であれば 1 年間治療を完遂した場合を、B 群であれば 1 年間治療を完遂した場合を、プロトコル治療終了と定義する。投与完遂例の詳細な定義は 12.2 項を参照のこと。

7.4. 後治療

「7.1　治療中止基準」のいずれかに該当し本治療を中止した場合、特別な理由がない限り、再発が明らかに認められるまで支持療法のみで経過観察する。再発または再発以外のがん病変を認めた場合の治療は制限しないが、症例報告書にその内容を報告する。

8. 併用薬および併用療法、支持療法

8.1. 併用禁止薬および併用禁止療法

他の抗癌剤、フルシトシン、フェニトイン、ワルファリン、ダビガトラン(プラザキサ)、放射線療法、免疫療法、手術、ホルモン剤

8.2. 併用可能薬・併用可能療法

Grade 4 の白血球減少または好中球減少が認められた場合、G－CSF 製剤の使用は、保険適応範囲において可とする。但、抗癌剤と G-CSF 製剤の同日投与は行わない。その他、血小板減少、悪心・嘔吐、過敏症、その他の有害事象が発現した場合は、必要に応じ対症療法を積極的に実施する。

8.3. HBs 抗原陰性で HBc 抗体陽性 and/or HBs 抗体陽性例に対する検査と支持療法

「免疫抑制・化学療法により発症する B 型肝炎対策ガイドライン」では、HBs 抗原陰性例でも、HBs 抗体あるいは HBc 抗体を測定し、陽性例では HBV-DNA 定量を行うことが推奨されている。

HBs 抗原陰性例、HBs 抗体あるいは HBc 抗体陽性のような例においては、強力な免疫抑制剤の使用により HBV の再活性化が起こり、重症肝炎が発症することが報告されている。このため、厚生労働省研究班「難治性の肝・胆道疾患に関する調査研究(主任研究者：坪内 博仁)」および「肝硬変を含めたウイルス性肝疾患の治療の標準化に関する研究(主任研究：熊田 博光)」による「免疫抑制・化学療法により発症する

B 型肝炎対策ガイドライン」に基づき、化学療法開始前に以下の検査を行い、検査結果に従って以下の支持療法を行うことを推奨する。エンテカビル投与に際しては、肝臓専門医にコンサルトすることを強く推奨する。ただし、HBs 抗体単独陽性の場合で、HBV ワクチン接種歴が明らかな場合は対象外とする。

<u>本試験における B 型肝炎ウィルスの対応</u>
HBs 抗原陽性 → 登録禁止
HBs 抗原陰性 → HBc 抗体・HBs 抗体陰性 → 通常の対応
HBs 抗原陰性 → HBc 抗体・HBs 抗体陽性 → HBV-DNA 定量を行うことを推奨するが、詳細な対応は施設に一任（核酸アナログ投与・肝炎の定期的モニタリングなど）

1) 化学療法開始前に行う検査：HBV-DNA 定量
HBV-DNA 定量は、PCR 法またはリアルタイム PCR 法により実施する。より検出感度の高いリアルタイム PCR 法が望ましい。

2) 化学療法開始前の時点で、HBV-DNA 定量が検出感度以上の場合
HBs 抗原陽性例と同様にエンテカビルを投与する。
①検査：HBV-DNA 定量
化学療法開始から化学療法終了後のエンテカビル投与終了後 12 ヶ月までは、4 週毎に HBV-DNA 定量を行う。ただし、エンテカビル投与中で、かつ HBV-DNA 定量で検出感度未満の場合は、外来通院の期間を考慮し、検査間隔を延長しても良い。
HBe 抗原および HBe 抗体の検査も、適宜行うことを推奨する。

②使用薬剤：エンテカビル水和物錠(ブリストル・マイヤーズ：バラクルード錠 0.5mg)
下記の用法用量に従い、化学療法開始前後できる限り早期にエンテカビルの投与を開始し、化学療法終了後、少なくとも 12 か月間継続する。化学療法終了 12 ヶ月後以降、エンテカビルの投与を終了する場合は、HBV-DNA 定量で検出感度未満であることを確認の上で、肝臓専門医と相談し、終了時期を決めること。エンテカビル投与終了後にも再活性化があり得ることを念頭におき、慎重に HBV-DNA 定量により経過観察を行うこと。

- 用法：空腹時(食後 2 時間以降かつ次の食事の 2 時間以上前)に経口投与する。
- 用量：

クレアチニンクリアランス (mL/min)	用量
50 以上	0.5 mg を 1 日に 1 回
30 以上 50 未満	0.5 mg を 2 日に 1 回
10 以上 30 未満	0.5 mg を 3 日に 1 回
10 未満	0.5 mg を 7 日に 1 回

3) 化学療法前の時点で、HBV-DNA 定量で検出感度未満の場合
HBV-DNA 定量または肝機能(AST、ALT)のいずれかによるモニタリングを行う。

①検査：HBV-DNA 定量、または肝機能(AST、ALT)
厚生労働省研究班ガイドラインでは、化学療法中および化学療法後 12 か月間は 4 週間ごとの HBV-DNA 定量によるモニタリングを推奨している。しかし、同ガイドラインは、リツキシマブ併用化学療法など HBV 再活性化ハイリスク例のデータをもとに作成されており、固形腫瘍を対象としたエビデンスは限られているため、再活性化リスクが低いことが予想される化学療法例においては、費用対効果の面で検討の余地がある。定期的な HBV-DNA 定量モニタリング以外の方法として、慎重に肝機能(AST、ALT)をモニタリングし、異常が見られた場合は適宜 HBV-DNA 定量を行う対策法が選択肢として挙げられる。ただし、HBV 再活性化による肝障害・肝炎が起こってから抗ウイルス薬を投与しても、救命できなかった(劇症肝炎による死亡)との報告もあるため慎重なモニタリングが必要である。

以上の背景、および、用いる化学療法による HBV 再活性化リスクを勘案し、本試験では、HBV-DNA 定量、あるいは肝機能(AST, ALT)のいずれかによるモニタリングを強く推奨する。HBV-DNA 定量で検出感度以上となった場合、上記 2)の用法・用量に準じて直ちにエンテカビルの投与を開始する。

9. 予期される薬物有害事象

9.1. 薬剤情報

一般名：S-1、商品名：TS-1、製造販売元：大鵬薬品工業。詳細は添付文書を参照のこと。最新情報は、http://www.info.pmda.go.jp/ で確認可能。

9.2. 有害事象の定義

有害事象とは、試験治療を受けたことによって被験者に生じたあらゆる好ましくないあるいは意図しない徴候（臨床検査値の異常変動を含む）、症状または疾病のことであり、試験治療との因果関係の有無は問わない。

9.3. 有害事象/有害反応の評価

NCI Common Terminology Criteria for Adverse Events v4.0（日本語訳版；添付)に従う。試験治療開始前（ベースライン）と比べて Grade が 1 以上悪化したものを有害事象とする。

9.4. 予期される有害反応

各添付文書参照。最新情報は、http://www.info.pmda.go.jp/ を確認。

9.5. 試験薬との因果関係

有害事象における試験薬との因果関係の判定に際しては、被験者の全身状態、合併症、併用薬・併用療法、時間的関係を勘案して判断する。なお、因果関係の判断は「試験薬との合理的な因果関係がある」、「試験薬との合理的な因果関係がない」の 2 つの判定区分を用いる。有害事象のうち、試験治療との合理的な因果関係がある場合を副作用として取り扱うこととする。

10. 有害事象の報告

“重篤な有害事象”もしくは“予期できない有害事象”が生じた場合、施設研究責任者は研究代表者/研究事務局へ報告する。

なお、各施設の医療機関の長への報告、厚生労働省事業「医薬品等安全性情報報告制度」による医療機関から厚生労働省医薬局への自発報告や、薬事法に基づく「企業報告制度」による医療機関から企業への自発報告は、それぞれの医療機関の規定に従って、各施設研究責任者の責任において適切に行うこととする。

10.1. 急送報告義務のある有害事象

以下のいずれかに該当する有害事象は<u>急送報告</u>の対象となる。

(1) プロトコル治療中または最終プロトコル治療日から 30 日以内のすべての死亡。
　プロトコル治療との因果関係の有無は問わない。プロトコル治療中止例の場合、後治療が既に開始されていても、最終プロトコル治療日から 30 日以内であれば急送報告対象となる(「30 日」とは、最終プロトコル治療日を day 0 とし、その翌日から数えて 30 日を指す)。

(2) 予期されない Grade 4 非血液毒性（CTCAEv.4.0-JCOG における血液/骨髄区分以外の有害事象）：最新の添付文書に記載されないものが該当する。

10.2. 通常報告義務のある有害事象

以下のいずれかに該当する有害事象は<u>通常報告</u>の対象となる。

(1) 最終プロトコル治療日から 31 日以降で、プロトコル治療との因果関係が否定できない死亡。治療関連死の疑いがある死亡が該当。明らかな原病死は該当しない。

(2) 予期される Grade4 非血液毒性(CTCAEv.4.0-JCOG における血液/骨髄区分以外の有害事象)

(3) 予期されないGrade3の有害事象
「予期される薬物有害事象」の項に該当しないGrade 3相当の有害事象
(4) 永続的または顕著な障害
再生不良性貧血、骨髄異形成症候群、二次がん等
(5) その他重大な医学的事象
上記のいずれにも該当しないが、研究代表者・研究グループ全施設で共有すべきと思われる重要情報と判断されるもの

10.3. 施設研究責任者の報告義務と報告手順

急送報告

(1)1次報告：当該症例担当医は、72時間以内に「AE/AR/ADR急送一次報告書」に所定事項を記入し、研究事務局へFAX/電話連絡し、可及的速やかに施設研究責任者への報告も行う。
(2) 2次報告：「ADR報告書」および詳細を記入した「症例報告の詳細(A4自由形式)」を作成し、両者を7日以内に研究事務局へファックスする。
(3)3次報告：原則として「ADR報告書」の所定事項をすべて記入し、そのコピーを有害事象発生を知りえてから15日以内に研究事務局へファックスする。
(4)追加報告：死亡の場合の剖検報告書など、3次報告以降に得られた情報や3次報告で未記入があった場合の追加情報がある場合、研究事務局へ報告する。

通常報告

(1)「ADR報告書」にて、急送報告に準じて15日以内に報告を行う。

10.4. 研究代表者/研究事務局の責務

(1)登録停止と施設への緊急通知の必要性の有無の判断
施設研究責任者から報告を受けた研究代表者または事務局は、報告内容の緊急性、重要性、影響の程度を判断し、必要に応じて登録の一時停止（登録センターと全参加施設への連絡）や、参加施設への周知事項の緊急連絡の対策を講ずる。登録センターや施設への連絡においては緊急度に応じて電話連絡も可だが、追って可及的速やかに文書(FAX、郵送、E-mail）による連絡も行う。
(2)効果・安全性評価委員会への報告
研究代表者/事務局は、施設から急送報告もしくは通常報告された有害事象が「報告義務のある有害事象」に該当すると判断した場合、有害事象の発生を知りえてから15日以内に効果安全性評価委員会宛に文書（FAX、郵送、E-mail）で報告し、同時に当該有害事象に対する研究代表者の見解と有害事象に対する対応の妥当性についての審査を依頼する。

11. 観察・検査項目および実施時期

11.1. 治療期間の定義

各被験者の治療期間は、登録日から最終投与日の４週後まで又は最終投与日から４週以内の後治療開始時までとする。
研究責任医師または研究分担医師は以下の内容を登録及び投与開始前に調査・確認する。

11.2. 手術前の評価項目

胸部X線/CT、頭部CTまたはMRI、腹部CT、骨シンチ。腹部・骨病変検索はPETで代用可。

11.3. 治療開始前（登録時）の評価項目（登録前2週間以内）

１）患者背景
性別、年齢、身長、体重、体表面積、手術日、病理病期（病期・TNM分類)、病歴、PS、病理組織所見、合併症の有無と疾患名および治療法、併用薬剤、喫煙歴（本数x期間）
２）自他覚症状
悪心/嘔吐等消化器症状、倦怠感、発熱、皮膚症状、味覚異常、呼吸困難感、流涙、その他の症状
３）血液一般/生化学/ガス

・血液一般（白血球/分画、ヘモグロビン、血小板）
・血液生化学（Alb、T-Bil、AST、ALT、LDH、BUN、Ccr 又は Cr、Na、K、Cl、Ca、CRP）
・血液ガス検査または SpO_2

4）対象病変観察：胸部レントゲン、必要に応じて胸部ＣＴ
5）腫瘍マーカー：CYFRA、CEA
6）尿検査：蛋白、糖、ウロビリノーゲン、潜血

11.4. 治療期間中の評価項目

各種検査項目および被験者の状態の評価を行う。
1)全身状態：　ＰＳ，体重測定
2)自他覚症状：　11.3 (2)と同じ
3)血液一般/生化学/ガス：11.3.(3)と同じ
4)対象病変観察：　11.3.(4)と同じ
5)腫瘍マーカー：　11.3.(5)と同じ
6)尿検査：　11.3.(6)と同じ

項目/時期	治療開始前	治療中/(1～２週毎)	治療終了時
患者背景	○		
自他覚所見・ＰＳ	○	○	○
対象病変観察<br>（新規病変検索）	○	原則２ヵ月<br>(必要に応じて適宜施行)	○
血液一般/生化学/ガス・体重測定	○	○	○
腫瘍マーカー	○	原則１ヵ月*	○
尿検査	○	必要に応じて	必要に応じて

何らかの有害事象が発現した場合、上記項目すべてにおいて規定されている日以外でも、その経過（最異常度、回復または軽快日など）を十分観察できる頻度で適宜施行する。
*保険適応の範囲内で実施する。

11.5. 薬剤投与中止/終了後

投与中止/終了後～初回投与より通算して２年間

原則として３ヶ月に１回診察、胸部単純レントゲンを行う。また３～６ヶ月毎に胸部 CT を含む画像検査、腫瘍マーカー（CEA、CYFRA)、必要に応じて血液検査(ヘモグロビン、白血球数および分画、血小板数、Alb、T-Bil、AST、ALT、LDH、BUN、Cr、Na、K、Cl、Ca)を行う。

投与中止/終了後～初回投与より通算して３年目～５年間

原則として６ヶ月に１回診察、胸部単純レントゲンを行う。また６～１２ヶ月毎に胸部 CT を含む画像検査、腫瘍マーカー（CEA、CYFRA)、必要に応じて血液検査(ヘモグロビン、白血球数および分画、血小板数、Alb、T-Bil、AST、ALT、LDH、BUN、Cr、Na、K、Cl、Ca)を行う。

11.6. 再発の兆候を認めた場合

術後いつの時点においても、再発の兆候を認めた場合は随時検査(胸部 CT、骨シンチ、頭部 CT or MRI、腹部 CT 等、必要に応じて PET 検査)を行う。

12. 評価項目の評価方法

12.1. 評価項目

Primary endpoint　:　投与完遂率（Feasibility）
Secondary endpoint　:　有害事象発生割合と程度、無再発生存期間(RFS)、全生存期間(OS)

12.2. 評価方法

投与完遂率（Feasibility）

投与完遂の定義

プロトコルに規定されている減量・休薬基準に従いながら、投与実施割合が 70%以上かつ術後化学療法としての S-1 投与治療を半年間治療完遂できた症例をプロトコル治療完遂（投与完遂）症例と定義する。

S-1 実総投与量/予定総投与量の割合

投与実施割合(%) =全投与期間での実際の処方量の合計(mg)/全服薬予定量(mg)　x 100
ただし、　全服薬予定量＝初回投与量(mg/day)　x　全投与予定日数(day)、かつ、
全投与期間での実際の服薬量の合計(mg) ＝ Σ(半年間あるいは治療中止までの総服薬量)、
と定義する。

最終的に主要評価項目評価に関し、「プロトコル治療完遂」症例の再発によるプロトコル途中中止例や打ち切り（途中脱落）例を除いた全適格例に対する割合を算出する。また、プロトコル治療 9 ヶ月時点、1 年時点等の治療完遂率も算出する。

無再発生存期間（RFS：Recurrence-free survival）　**削除:** Relapse

・登録日を起算日とし、再発と判断された日またはあらゆる原因による死亡日のうち早い方までの期間。
・「再発 recurrence」は、画像診断に基づいて判断されるものと、画像診断に依らない病状の増悪による再発の判断（臨床的再発）の両者を含む。画像診断に基づいて再発と判断した場合はその画像検査を行った検査日を再発日とし、臨床的再発の場合は臨床的判断日を再発日とする。腫瘍マーカーの上昇のみの期間は再発とせず、画像診断で再発を確認した検査日または病状の増悪により臨床的に再発の判断を行った日をもって再発とする。　**削除:** relapse
・ 再発と判断されていない生存例では、最終生存確認日をもって打ち切りとする（電話連絡による生存確認も可。ただし生存確認を行ったことをカルテに記録すること）。
・ 毒性や患者拒否などの理由による化学療法中止例で、後治療として他の治療が加えられた場合も、イベントと打ち切りは同様に扱う。すなわち、治療中止時点や後治療開始日で打ち切りとしない。
・ 再発の診断が画像診断による場合、「画像上疑い」の検査日ではなく、後日「確診」が得られた画像検査の「検査日」をもってイベントとする。画像診断によらず臨床的に再発と判断した場合は、再発と判断した日をもってイベントとする。
・ 再発の確定診断が生検病理診断による場合、生検前に臨床上再発と診断し得た場合は臨床診断日を、臨床上再発と診断し得ず生検病理診断によって再発と診断した場合は生検施行日をもってイベントとする。
・ 二次がん（異時性重複がん）の発生はイベントとも打ち切りともせず、他のイベントが観察されるまで無再発生存期間とする。
・ 完遂割合により層別した無再発生存期間の違いも明らかにする。

全生存期間(OS：Overall survival)

術後に S-1 を始めて投与した日を起算日とし、あらゆる原因による死亡日までの期間。
・ 生存例では最終生存確認日をもって打ち切りとする（電話連絡による生存確認も可。ただし生存確認を行ったことをカルテに記録すること）。
・ 追跡不能例では追跡不能となる以前で生存が確認されていた最終日をもって打ち切りとする。
・ 完遂割合により層別した生存期間の違いも明らかにする。

有害事象（有害反応）発生割合

全治療例を分母とし、試験治療によると判断される有害事象発症を分子として、CTCAE v4.0 日本語訳 JCOG 版による全コース中の最悪の Grade の割合を求める。

13. 目標症例と試験実施期間

13.1. 症例集積期間/追跡期間

症例集積期間：2012 年月 5 月～2016 年 4 月（4 年間）
主要評価項目に関する追跡期間：登録終了後 6 ヶ月
研究期間：2012 年 4 月～2022 年 6 月

全登録患者のプロトコル治療と完遂割合の評価が終了する時期である登録終了 6 ヶ月後を目途に全ての評価項目に対する解析を行う。また副次的評価項目については、登録終了後 5 年間を追跡期間とし、2 年後および追跡期間終了時に生存解析を行う。解析を実施する主体は最終症例登録終了後 2 年目まではデータセンター、それ以降は事務局とするが、事務局で協議の上最終決定する。本試験で設定した主要評価項目の解析が終了した後、その結果の公表や副次的評価項目解析上バイアスがかからないと判断される場合に限り、データセンターは事務局の依頼に応じて、データセンターに存在する本試験関連のデータ情報・解析結果内容の一切を事務局へ無償譲渡することとする。

13.2. 解析の対象となる被験者の選択

本試験では、全登録例、全適格例及び全治療例を下記と定義し、最終解析における安全性評価は「全治療例」を用い、有効性評価には「全登録例」又は「全適格例」を用いる。

１）全登録例
登録された患者のうち、重複登録や誤登録を除いた集団。

２）全適格例
全登録例から、グループでの検討によって検討された「不適格例」を除く集団。不適格例の決定に際しては、最終解析においてグループ代表者の承認を要するが、定期モニタリング、最終解析レポート提出以前の学会発表の際の解析においては、研究事務局の了解の上で全適格例に事務局判定による「不適格例」を含めないことができる。

３）全治療例
全登録例のうち、プロトコル治療の一部又は全部が施行された全患者。

13.3. 症例数の設定根拠

評価項目の評価方法の項の「投与完遂割合の定義」を用いて主要評価項目に関する解析を実施する場合、再発によるプロトコル途中中止例は打ち切り（途中脱落）の扱いとすべきであり、解析上対象症例としてそぐわないため、ここでは全適格例からこれらを除いた症例数で、「投与治療完遂」の割合を算出する。

Stage I B 以上の非小細胞肺癌完全切除例を対象にした S-1 80 mg/m²/day 2 週投与 1 週休薬（半年投与）の LOGIK0601 試験では、投与完遂は 56.7%(95%CI:37.4～74.5、相対服用率 73.9)、70 歳以上の投与完遂率は 42.9%(6/14)という結果が得られている[16]。一方、高齢者を対象としたデータではないが、胃癌術後の S-1 を隔日投与(1 年) は、通常投与法(1 年間)よりもコンプライアンスおよび RDI がそれぞれ 19.6 %、13.7 % 高いという結果が得られている（91.8% vs 72.2%、81.2% vs 67.5%）[15]。

隔日投与法、あるいは、2 週投与 1 週休薬法の半年間投与治療完遂割合のうち、良い方を 55%、悪い方を 40%と仮定する。この 15%の差を正しく選択する確率を 90%で検出する症例数を Selection theory に基づき算定すると各群 37 症例ずつ必要となる。脱落例を想定すると計 100 症例を要する。予定年間登録数は 50 例が見込まれると考え、登録期間は 2 年間とする。

なお、ITT 解析を実現すべく、全登録例を対象として、各症例の S-1 実総投与日数の Kaplan-Meyer 曲線を描く。S-1 の毒性による内服中止の時点でイベント発生とするが、S-1 に直接関係しない

complication（再発、２次癌）のため途中中止となった場合は、その時点で打ち切りとする。
Kaplan-Meyer　曲線より算出される継続投与率も副次的に評価し、本試験治療のコンプライアンスの指標項目とする。

13.4.　試験終了後の結果による標準的治療法(S-1 投与法)の Decision criteria

本研究は、隔日投与が連日投与法よりも継続性の優れた投与法であることを検証する試験である。しかしながら、投与継続性の差だけで術後補助化学療法の真の目的である survival benefit が得られるとは限らず、副作用の程度や QOL を含めた形で評価すべきである。

そこで、試験終了時、将来の第三相試験に向けてどちらの試験群を選択するかの Decision criteria は、以下の通りとする。

①両群ともに半年間投与治療完遂割合が 40%以下である場合は、両群ともに本対象における promising な術後補助化学療法ではないと結論する。
②一方の群において半年間投与治療完遂割合が 40%を超え、かつもう一方の群と 15%以上の差があった場合には、半年間投与治療完遂割合の高い群の治療法が本対象における promising な術後補助化学療法であり、将来の第三相試験における S-1 の投与法としてより適していると結論する。
③一方の群において治療完遂率が 40%を上回り、かつもう一方の群との差が 15%以内であった場合には、治療継続率の数字だけで両群の直接の比較は行わず、毒性、QOL、利便性、Cost、6 ヶ月以降の治療継続率、無再発生存率、生存率などを総合的に加味して、どちらが将来の第三相試験における S-1 療法としてより適しているかを決定する。

14.試験の中止

症例登録が予定症例登録数に達した時点で登録終了とするが、同時点で既に試験参加の説明をおこなっていた適格患者に関しては登録を受けつけることとする。また、試験の進行中に重篤な有害事象または試験薬・治療法の新たな情報等により、患者の安全を著しく損ない、試験全体を中止せざるを得ないと判断された場合、効果安全性評価委員会は研究代表者に対し、試験中止を勧告する。研究代表者は勧告内容を検討し、臨床試験審査委員会の承認を得て試験中止を決定する。中止決定後は速やかに参加施設にその旨および中止理由の詳細を文書で連絡する。

15.データの収集および保存

15.1.　データの収集

本試験で用いる症例報告書（Case Report Form:CRF)と提出期限は以下の通りとする。原則下記すべての CRF は郵送にて送付する。送付先はデータセンターとする。

①被験者基本データ・・・・・・・・・・	登録後すみやかに
②抗がん剤投与記録用紙・・・・・・・	治療中止時/終了後２週間以内
③治療終了報告用紙・・・・・・・・・	治療中止/終了後２週間以内
④追跡調査・・・・・・・・・・・・・	１年ごとまたは再発確認時
⑤血液検査、自他覚異常値所見等・・・・	治療中止時/終了後２週間以内
⑥追跡報告用紙・・・・・・・・・・・	事務局依頼時

15.2.　記録の保存

記録は、データセンターにて記録の保存に関する「標準手順書」に従い、保管すべき試験に係わる文書および記録を保管する。保管期間は当該研究の終了について報告された日から５年を経過した日または当該研究の結果の最終公表について報告された日から３年を経過した日のいずれか遅い日までの期間とする。患者の同意に関する記録、報告書作成に係わるデータ(検査データ等)、IRB/IEC 承認書、参加医療機関において作成された記録文書等については試験責任医師が保管する。保管期間は当該研究の終了について報告された日から５年を経過した日または当該研究の結果の最終公表について報告された日から３年を経過した日のいずれか遅い日までの期間とする。

16. 倫理的事項

16.1. 被験者の保護

本研究に係わるすべての研究者は、「ヘルシンキ宣言」および「臨床研究法」を遵守して実施する。研究実施に係る情報を取扱う際は、研究独自の研究対象者コードを付して管理し、研究対象者の秘密保護に十分配慮する。研究の結果を公表する際は、氏名、生年月日などの直ちに研究対象者を特定できる情報を含まないようにする。また、研究の目的以外に、研究で得られた研究対象者の情報を使用しない。

16.2. 同意の取得

主治医は被験者の登録前に、同意説明文書を用いて下記項目の十分な説明を行なう。被験者に対して質問する機会と試験に参加するか否かを判断するのに十分な時間を与える。被験者が本試験の内容を十分に理解したことを確認後、被験者本人の自由意思による参加同意を文書により取得する。主治医は署名された同意説明文書の写しを被験者に速やかに手渡す。同意文書の原本はカルテに保存する。保管期間は当該研究の終了について報告された日から5年を経過した日または当該研究の最終公表について報告された日から3年を経過した日のいずれか遅い日までの期間とする。

16.3. 説明事項

1) 背景、2) この試験の目的、3) 本臨床試験での治療方法/内容、4) 予想される医学上の貢献、5) この試験の実施期間、6) この試験への予定参加人数について・研究実施場所、7) 予想される効果と副作用について、8) 他に有効な治療法、9) 研究に使用する試料、10) あなたの健康に被害が生じた場合、11) 自由意思に基づいた同意、12) 新たに重大な情報が得られた場合、13) 臨床試験途中での中止の可能性、14) 個人情報の保護・人権の保護・研究結果の開示、15) 注意していただきたいこと、16) あなたの治療費用負担について、17) 研究資金、利益相反、研究利益、18) この研究に参加した場合に受ける利益、不利益、危険性、19) データの二次利用について 20) 連絡先

16.4. プライバシーの保護と被験者識別

登録患者の同定や照合は、登録時に発行される登録番号を用いて行われ、登録患者氏名は他施設へ知らされることはない。また、患者名・診療録番号等個人識別可能な情報が、事務局に登録されることはない。個人情報が漏洩する可能性を完全除去することは出来ないが、本研究参加医師は考えられるリスクを踏まえた上で、個人情報保護のため最大限の努力を払う。

16.5. プロトコルの遵守

本試験に参加する研究者は患者の安全と人権を損なわない限りにおいて本研究実施計画書を遵守する。

16.6 施設の試験審査委員会（IRB）または倫理審査委員会の承認

本試験への参加に際しては、本研究実施計画書および患者への説明文書が各施設の IRB または倫理審査委員会で承認されなければならない。IRB または倫理審査委員会の承認が得られた場合、各施設の研究責任者は承認書のコピーを研究事務局に送付する。承認書原本は当該施設が保管、コピーは研究事務局が保管する。

16.7. プロトコルの内容変更について

<u>プロトコルの内容変更の区分</u>

プロトコル内容変更の際には、変更内容の実行に先立って「プロトコル改定申請」を効果・安全性評価委員会に提出し承認を得なければならない。

プロトコル内容の変更を改正・改訂の2種類に分けて取り扱うが、この改正・改訂の区別は効果・安全性評価委員が行う。プロトコル内容の変更に該当しない補足説明追記はメモランダムとして区別する。

①改正：試験に参加する患者の危険を増大させる可能性のある、又は主要評価項目に関する部分的変更。
②改訂：試験に参加する患者の危険を増大させる可能生がなく、かつ主要評価項目にも関連しない変更。
③メモランダム/覚書：内容変更ではなく、文面の解釈上のばらつきを統一、特に注意を喚起するなどの目的で、研究代表者/研究事務局から試験の関係者に配布するプロトコルの補足説明。

プロトコル改正/改訂時の施設 IRB または倫理審査委員会承認

試験中に効果・安全性評価委員の承認を得て本研究実施計画書または患者への説明文書の改正があった場合は、改正された本研究実施計画書または患者への説明文書が各施設の IRB または倫理審査委員会で承認されなければならない。

内容変更が改正ではなく改訂の場合に、各施設の IRB または倫理審査委員会の承認審査を必要とするか否かは各施設の取り決めに従う。

改正に対する IRB または倫理審査委員会の承認が得られた場合、各施設の研究責任者は承認書のコピーを研究事務局に送付する。承認書原本は施設が保管、コピーは事務局が保管する。

16.8. 症例報告用紙（CRF）の内容変更について

試験開始後に、CRF に必要なデータ項目の欠落や不適切なカテゴリー分類等の不備が判明した場合、「観察・検査・評価項目とスケジュール」の項で規定した収集データの範囲を超えず、かつ CRF の修正により登録患者の医学的・経済的負担を増やさないと判断される限りにおいて、効果安全性委員会と研究事務局の合意の上で CRF の修正を行う。プロトコル本文の改訂を要さない CRF の修正はプロトコル改訂としない。CRF の修正に関する医療機関の長への報告や改訂申請の有無は施設の規定に従う。

16.9. データの二次利用について

本試験で得られたデータについては、個人識別情報とリンクしない形でデータを二次利用（メタアナリシスなど）することがあり得る。

17. モニタリングと監査

試験が安全にかつプロトコルに従って実施されているか、データが正確に収集されているかを確認する目的で、研究事務局・研究代表者を中心に必要に応じてモニタリングが行われる。CRFは研究責任医師/分担医師またはCRC（clinical research coordinator）が作成する。モニタリングは研究事務局に収集される記録用紙（CRF）の記入データに基づいて行われる中央モニタリングであり、施設訪問にて原資料との照合を含めて行う施設モニタリングは行わない。患者を直接識別できる情報が事務局のデータベースに登録されることはない。個人的情報が厳重に守られるよう最大限配慮を行う。

17.1. モニタリングの項目

集積達成状況、患者適正、治療完遂割合、プロトコル治療/終了状況、重篤な有害事象、有害反応/有害事象、プロトコル逸脱、その他、研究の進捗や安全性に関する問題点

17.2. プロトコル逸脱

(1)違反：原則として以下の複数項目に該当するプロトコル規定からの逸脱を「違反」とする。
①主要評価項目に影響を及ぼす、②担当医/施設に原因がある、③故意または系統的、④危険または逸脱の程度が甚だしい、⑤臨床的に不適切である

(2)逸脱：違反にも許容範囲にも該当しない逸脱。特定の逸脱が多く認められた場合は、試験結果公表の際に、その旨記載する。

(3)許容範囲：研究グループと研究事務局間で事前に決定する。

18. 費用と補償

本試験で用いるS-1は、既に非小細胞肺癌における保険適応を取得しているため、治療にかかる費用は通常の保険診療による負担で行われる。本試験治療により健康被害が生じた場合においても一般診療で対処され、保険診療と同様、被験者の一部自己負担となる。入院費、あるいは交通費などの特別な補償はない。また試験参加医師は医師賠償保険に加入することを推奨する。

19. 研究資金および利益の衝突

本研究の資金は、特定非営利活動法人疫学臨床試験研究支援機構の資金により実施する。
本研究の計画・実施・報告において、研究の結果および結果の解釈に影響を及ぼすように「起こり得る利益の衝突」は存在しない。および研究の実施が被験者の権利・利益を損ねることがないことを確認する。

20. 研究に関する情報公開の方法（研究計画の登録および研究結果の発表）

本研究は、臨床研究実施計画・研究概要公開システム(jRCT)に登録する。また、本研究で得られた結果は、国内外の主要な学会等で発表し、英文の専門学術誌に論文として公表する予定である。いずれの場合においても、公表する結果は統計的な処理を行ったものだけとする。公表論文の著者は登録数の多い順に施設研究責任者もしくは施設コーディネーターを施設毎に選ぶことを原則として、最終的には研究代表医師が決定する。

研究代表医師は、主要評価項目報告書並びに総括報告書及びその概要を作成する。
①主要評価項目報告書：主たる評価項目に係るデータの収集を行うための期間が終了してから1年以内
②総括報告書及びその概要：全ての評価項目に係るデータの収集を行うための期間が終了してから1年以内
報告書を認定臨床研究審査委員会に提出し、意見を聴くとともに、認定臨床研究審査委員会が意見を述べた日から1か月以内にjRCTに公開する。また、研究計画書、総括報告書の概要を厚生労働大臣に提出する。
研究代表医師は、主要評価項目報告書又は総括報告書の概要を公表したときは、速やかに、実施

医療機関の管理者に報告するとともに、その旨を他の研究責任医師に情報提供する。この場合において、当該他の研究責任医師は、速やかに、当該情報提供の内容を実施医療機関の管理者に報告する。

21. QOL 解析

参加可能な施設で実施する。

21.1. 必要な試料

別添の QOL 調査票（試料）「ケアーノート」

21.2. 調査方法

登録より 12 ヶ月間にわたり患者が規定の QOL 調査票に記入する。

21.3. 試料送付

該当患者が記入終了した段階で、担当医師がデータセンターへ郵送する

21.4. 試料解析

京都大学大学院医学研究科医学統計生物情報学で解析を行う。また、QOL 調査については岡山大学病院呼吸器内科で解析を行う。

21.5. 試料の解析後処理

試料は解析終了後、岡山大学病院呼吸器外科で本試験終了後５年間保管する。

22. 疾病等報告

本研究の実施に起因することが疑われる疾病・死亡・障害・感染症が発生した場合には、認定臨床研究審査委員会への報告を行うとともに、予期しない重篤なものについては、厚生労働大臣への報告を行う。

本研究の実施によると疑われる重篤な疾病等は、以下の通り定義する。

①死亡
②死亡につながるおそれのある疾病等
③治療のために医療機関への入院又は入院期間の延長が必要とされる疾病等
④障害
⑤障害につながるおそれのある疾病等
⑥③から⑤まで並びに死亡及び死亡につながるおそれのある疾病等に準じて重篤である疾病等
⑦後世代における先天性の疾病又は異常

研究責任医師及び研究分担医師（研究責任医師等）は、特定臨床研究の実施において重篤な疾病等の発生を知った場合には、研究対象者等への説明等、必要な措置を講じなければならない。

本研究は多施設共同研究であるため、疾病等報告は研究代表医師が行う。研究責任医師は、疾病等の発生を知ったときは、これを実施医療機関の管理者に報告した上で、研究代表医師に通知し、また、その旨を速やかに他の研究責任医師に情報提供する。この場合において、当該他の研究責任医師は、速やかに当該情報提供の内容を実施医療機関の管理者に報告する。研究代表医師は、厚生労働省令で定めるところにより、統一様式８を用いて、疾病等報告を当該特定臨床研究の実施計画に記載されている認定臨床研究審査委員会に報告しなければならない。報告期限は以下のとおりである。

予測性	疾病等	認定臨床研究審査委員会への報告	厚生労働大臣への報告
予測できない	上記①・②	7日	7日
	上記③～⑦	15日	15日
予測できる	上記①・②	15日	―
	上記③～⑦	定期報告時	―

23. 研究組織

23.1. 研究代表者（瀬戸内肺癌研究会）

岡山大学大学院医歯薬学総合研究科呼吸器・乳腺内分泌外科学　豊岡伸一

23.2. 研究事務局

岡山大学病院呼吸器外科　諏澤憲、山本寛斉、岡﨑幹生
電話　086-235-7265　　　FAX　086-235-7269

23.3. 参加予定施設

岡山大学病院呼吸器外科
関西医科大学附属病院呼吸器腫瘍内科
川崎医科大学附属病院呼吸器外科
倉敷中央病院呼吸器外科・内科　他

23.4. 効果・安全性評価委員会（順不同、敬称略）

田端雅弘：岡山大学病院　腫瘍センター
西井研治：岡山県健康づくり財団附属病院
永安武：長崎大学大学院医歯薬学総合研究科

23.5. 統計解析・登録センター

九州大学大学院医学研究院予防医学分野　松尾恵太郎（計画書 Ver 1.4 まで）
京都大学大学院医学研究科医学統計生物情報学　森田智視（計画書 Ver 1.5 より）

23.6. データセンター

特定非営利活動法人　疫学臨床試験研究支援機構（ECRIN）
理事　坂本純一
　公立学校共済組合東海中央病院
愛知支部　担当　荒井恵子・宮下由美
　　電話：0564-64-7300　FAX:0564-64-7301

24. 参考文献

1) N Engl J Med 2004;350:351-60.
2) N Engl J Med 2005;352:2589-97.
3) Lancet Oncol 2006;7:719-27.
4) J Clin Oncol 2005;23:4999-5006.
5) 癌と化学療法 1998;25:371-384.
6) Anticancer Drugs 1996;7:548-557.
7) Br J Cancer 2001;85:939-943.
8) 第50回日本肺癌学会(2009)、P-194 群馬大学大学院　病態外科学　清水公裕ら
9) Br J Cancer 2005;93:884.
10) Gastroenterogy 1963;45;721-9.
11) Cancer 1965;18:1189-213.
12) Little Brown & Co.1960;56:70-98.
13) Int J Clin Oncol. 2004;9:143-8.
14) 第49回日本癌治療学会(2011), OS38-1, 和歌山県医大　山上ら
15) S. Nakamura et al. ASCO-GI2012　＃114「Randomized controlled phase II study of alternate-day S-1 as adjuvant chemotherapy for gastric cancer.」
16) Lung Cancer 2010;67:184-187.
