# Supplementary material for: Randomized phase II study of daily versus alternate-day administrations of S-1 for the elderly patients with completely resected pathological stage IA (tumor diameter > 2 cm)—IIIA of non-small cell lung cancer: Setouchi Lung Cancer Group Study 1201
Source: PLoS One. 2023 May 19;18(5):e0285273. doi: 10.1371/journal.pone.0285273 (PMC10198543; doi:10.1371/journal.pone.0285273)
Supplement: S4 File — (PDF) [file pone.0285273.s027.pdf]

高齢者非小細胞肺癌完全切除後病理病期IA (T1bN0M0)/IB/II/IIIA期症例の術後補助化学療法に対するS-1の連日投与法および隔日投与法のランダム化第二相試験

研究実施計画書（別紙）

１．提供元及び提供先の機関名

提供元機関

倉敷中央病院　呼吸器外科　主任部長　奥村典仁　登録数：26例
　岡山県倉敷市美和1-1-1　086-422-0210
福島県立医科大学附属病院　呼吸器外科　講師　塩豊　登録数：11例
　福島県福島市光が丘1　024-547-1111
岡山大学病院　呼吸器外科　教授　豊岡伸一　登録数：9例
　岡山県岡山市北区鹿田町2-5-1　086-235-7265
川崎医科大学附属病院　呼吸器外科　教授　中田昌男　登録数：7例
　岡山県倉敷市松島577　086-462-1111
広島市立広島市民病院　呼吸器外科　主任部長　松浦求樹　登録数：6例
　広島県広島市中区基町7-33　082-221-2291
中国中央病院　呼吸器内科・腫瘍内科　部長　玄馬顕一　登録数：6例
　広島県福山市御幸町大字上岩成148-13　084-970-2121
日本赤十字社長崎原爆病院　呼吸器外科　部長　佐野　功　登録数：5例
　長崎県長崎市茂里町3-15　095-847-1511
国立病院機構長良医療センター　呼吸器外科　部長　藤永卓司　登録数：5例
　岐阜県岐阜市長良1300-7　058-232-7755
岡山済生会総合病院　外科 呼吸器病センター　センター長　片岡正文　登録数：5例
　岡山県岡山市北区国体町2-25　086-252-2211
佐賀県医療センター好生館　呼吸器外科　部長　寺﨑泰宏　登録数：3例
　佐賀県佐賀市嘉瀬町大字中原400　0952-24-2171
京都大学医学部附属病院　呼吸器外科　教授　伊達洋至　登録数：3例
　京都府京都市左京区聖護院川原町54　075-751-3111
岡山労災病院　腫瘍内科　部長　藤本伸一　登録数：3例
　岡山県岡山市南区築港緑町1-10-25　086-262-0131
国立病院機構岩国医療センター　胸部外科　診療部長　片岡和彦　登録数：3例
　山口県岩国市愛宕町1-1-1　0827-34-1000
島根県立中央病院　呼吸器外科　病院長　小阪真二　登録数：2例
　島根県出雲市姫原4-1-1　0853-22-5111
国立病院機構四国がんセンター　呼吸器外科　副院長　山下素弘　登録数：2例
　愛媛県松山市南梅本町甲160　089-999-1111
国立病院機構山口宇部医療センター　呼吸器外科　医長　井野川英利　登録数：2例
　山口県宇部市東岐波685　0836-58-2300
下関市立市民病院　呼吸器外科　部長　井上政昭　登録数：1例
　山口県下関市向洋町1-13-1　083-231-4111
鳥取大学医学部附属病院　胸部外科　教授　中村廣繁　登録数：1例
　鳥取県米子市西町36-1　0859-33-1111

国立病院機構呉医療センター 中国がんセンター　呼吸器外科　科長　山下芳典　登録数：1例
広島県呉市青山町 3-1　0823-22-3111

２．試料・情報の項目

記載あり

３．試料・情報の取得の経緯

記載あり

４．対応表の管理方法

（１）本研究で得られた試料・情報

各施設の研究責任者が適切に管理を行い、外部への提供は行わない。
