# Supplementary material for: Randomized phase II study of daily versus alternate-day administrations of S-1 for the elderly patients with completely resected pathological stage IA (tumor diameter > 2 cm)—IIIA of non-small cell lung cancer: Setouchi Lung Cancer Group Study 1201
Source: PLoS One. 2023 May 19;18(5):e0285273. doi: 10.1371/journal.pone.0285273 (PMC10198543; doi:10.1371/journal.pone.0285273)
Supplement: Attachment — Submitted filename: Yamamoto et al PONE-D-22-18798 Responses to the Reviewers.docx [file pone.0285273.s028.docx]

**PONE-D-22-18798**

**Yamamoto et al.**

**Randomized phase II study of daily versus alternate-day administrations of S-1 for the elderly patients with completely resected pathological stage IA (tumor diameter > 2 cm) - IIIA of non-small cell lung cancer: Setouchi Lung Cancer Group Study 1201**

**Responses to the Reviewers**

We thank the reviewers and the editor for their careful and conscientious reading of the manuscript and for their useful suggestions that have resulted in a much-improved manuscript. As indicated in the responses that follow, we have taken the comments and suggestions into account as much as possible in the revised version of our paper. The lines we indicate in the following comments are based on the revised manuscript with track changes.

***Comments by Reviewer #1 and our responses to each comment***

- The design: Why choosing feasibility based on the compliance as a primary endpoint for the study? How is compliance to treatment correlated to the harder endpoints such as OS or Disease-free survival. Were there any data to support the surrogacy? Understand that the current endpoint can be observed earlier (at 6 months) but the study is conducted for much longer time and there is a good opportunity to observe harder endpoint such as Recurrence free or progression survival rate.

**Response.**

We are grateful for the comments from Reviewer #1. As we focused on the feasibility of S-1 administration on elderly patients, we set the feasibility as a primary endpoint. As the Reviewer #1 pointed out, this endpoint can be observed earlier. However, it took more time to enroll the patients into this study than originally expected and thus the registration period was extended from 2 years to 4 years. As a result, we conducted the study much longer, getting the enough data on the survival. To improve the quality of this study, we put all the data including 5-year survival data into the article. We have no data to support that feasibility can be a surrogate to RFS or OS.

- Treatment completion was 69.4% in Arm A and 64.6% in Arm B yet the study Conclusion: Alternate-day oral administration of S-1 was demonstrated to be feasible with low toxicity in elderly patients with completely resected NSCLC solely focused on Arm A. Why was treatment B not mentioned to be feasible? Since both treatment arms passed the criteria to be feasible, was there any rule for choosing the better treatment Arm applied?

**Response.**

We set the decision criteria for standard treatment of S-1 administration based on the results as shown in the section 13.4. of the study protocol (attached as Supporting information). The following is the statement:

“If the completion rate in one group is greater than 40% and the difference between one group and the other is within 15%, the two groups will not be directly compared based on retention rate numbers alone, but rather on toxicity, quality of life, convenience, cost, retention rate after 6 months, recurrence-free survival, and survival rate to determine which is more suitable as S-1 therapy in future Phase III trials.”

As shown in Table 2, relative dose intensity of S-1 at 12 months is better in Arm A than in Arm B. As for the treatment completion rate, Table 3 shows that the completion rate at 12 months in Arm A (63.3%) is more than 15% higher than that in Arm B (45.8%). Table 3 also shows completion of S-1 administration without dose reduction or postponement at 12 months was better in Arm A than in Arm B. Regarding adverse events, anorexia, skin symptoms, and lacrimation were significantly more frequent in Arm B compared with Arm A as shown in Table 4. Considering the results above, we chose Arm A as the better treatment arm.

We added the following phrase in Line 267-270 in the section of “Study design and statistical analysis” in “Patients and Methods”:

3) If the 6-month treatment completion rate of one group is more than 40% and exceeds that of another group by 15% or less, the comparison between the two groups should not be performed with the treatment completion rate alone, but rather with toxicity, quality of life, convenience, cost, treatment completion rate after 6 months, recurrence-free survival (RFS), and overall survival (OS) to determine which is more suitable as S-1 therapy in future Phase III trials.

We also added the following phrase in Line 351-353 in the section of “Feasibility” in “Results”:

The treatment completion rate based on an RDI of 70% or more for 6 months in Arm A was greater than 40%. However, it did not exceed that of Arm B by more than 15%, indicating that the comparison between the two groups should not be performed with the treatment completion rate alone, but rather with toxicity, quality of life, convenience, cost, treatment completion rate after 6 months, RFS, and OS to determine which is more suitable as S-1 therapy in future Phase III trials.

We added the following sentence in Line 464-471 in “Discussion”:

As shown in Table 2, RDI of S-1 at 12 months is significantly better in Arm A than in Arm B. As for the treatment completion rate, Table 3 shows that the completion rate at 12 months in Arm A (63.3%) is more than 15% higher than that in Arm B (45.8%). Table 3 also shows completion of S-1 administration without dose reduction or postponement at 12 months was significantly better in Arm A than in Arm B. Regarding adverse events, anorexia, skin symptoms, and lacrimation were significantly more frequent in Arm B compared with Arm A as shown in Table 4. Considering the results above, we chose Arm A as the better treatment arm.

We revised “Results” and “Conclusion” in “Abstract” as follows:

**Results:** We enrolled 101 patients in which 97 patients received S-1 treatment. The treatment completion rate at 6 months was 69.4% in Arm A and 64.6% in Arm B (*p* = 0.67). Treatment completion rate in Arm B tended to be lower compared to Arm A, as the treatment period becomes longer (at 9 and 12 months). RDI of S-1 at 12 months and completion of S-1 administration without dose reduction or postponement at 12 months was significantly better in Arm A than in Arm B (*p* = 0.026 and *p* < 0.001, respectively). Among adverse events, anorexia, skin symptoms and lacrimation of any grade were significantly more frequent in Arm B compared with Arm A (*p* = 0.0036, 0.023 and 0.031, respectively). The 5-year recurrence-free survival rates were 56.9% and 65.7% for Arm A and B, respectively (*p* = 0.22). The 5-year overall survival rates were 68.6% and 82.0% for Arm A and B, respectively (*p* = 0.11).

**Conclusion:** Alternate-day oral administration of S-1 was demonstrated to be feasible with better treatment completion at 12 months and low toxicity in elderly patients with completely resected NSCLC.

- In the current study there seems to be a trend that arm B is slightly better in term of OS and recurrence free survival. If phase II is warranted, how will this be taking into consideration given the fact that feasibility show that arm A has slightly better compliance?

**Response.**

As described in Results and Discussion, Arm A had male and ever smoker cases more frequently compared to Arm B. Especially, both male and ever smoker cases with pStage IB were more frequent in Arm A than in Arm B (sex and smoking history were not adjustment factors). Male and ever smoking status are both reported to be poor prognostic factors in NSCLC. As shown in S6 and S7 Figs, these factors were also shown to be poor prognostic factors in the current study. They may have affected the trend of unfavorable prognosis in Arm A. As this study targets elderly patients and thus we focused on the feasibility, we chose Arm A as the better treatment arm.

Regarding this, we corrected the following sentence in Line 462 (textual error).

As shown in S6~~5~~ and S7~~6~~ Figs, these factors were also shown to be poor prognostic factors in the current study.

- Lines 395-398 “However, its drug discontinuation free survival rate, which is similar to the treatment completion rate at 1 year, was 52.7 %, suggesting that the compliance is not satisfactory despite no treatment-related death and low rate of any severe adverse events.”

Question: How is this with respect to your feasibility criteria (hypothesis). Is not Arm B passing the treatment completion rate according to the hypothesis in the protocol?

**Response.**

Although toxicity was less frequent in Group B (2-week administration followed by a 1-week rest period) compared to Group A (4-week administration followed by a 2-week rest period) in Setouchi Lung Cancer Group Study 0701, the compliance in Group B is not satisfactory, because the authors assumed that the drug discontinuation free survival rate at 1 year in Group B was 65% based on the previous report.

We added the following phrase in Line 436-437 in “Discussion”:

However, its drug discontinuation free survival rate, which is similar to the treatment completion rate at 1 year, was 52.7 %, lower than 65% (hypothesis in that study [25] based on the previous report [13]), suggesting that the compliance is not satisfactory despite no treatment-related death and low rate of any severe adverse events.

In the current study, as shown in Table 3, treatment completion rate in Arm B (2-week administration followed by a 1-week rest period) tends to be low compared to Arm A, as the treatment period becomes longer (at 9 and 12 months). Although the treatment completion rate at 6 months in Arm B was more than 40%, we consider that Arm B is still not passing, based on the statement of 13.4. of the study protocol (as shown below), as we focused on the feasibility.

The following is the statement of 13.4. of the study protocol:

If the completion rate in one group is greater than 40% and the difference between one group and the other is within 15%, the two groups will not be directly compared based on retention rate numbers alone, but rather on toxicity, quality of life, convenience, cost, retention rate after 6 months, recurrence-free survival, and survival rate to determine which is more suitable as S-1 therapy in future Phase III trials.

As already mentioned, we added the following sentence in Line 464-471 in “Discussion”:

As shown in Table 2, RDI of S-1 at 12 months is significantly better in Arm A than in Arm B. As for the treatment completion rate, Table 3 shows that the completion rate at 12 months in Arm A (63.3%) is more than 15% higher than that in Arm B (45.8%). Table 3 also shows completion of S-1 administration without dose reduction or postponement at 12 months was significantly better in Arm A than in Arm B. Regarding adverse events, anorexia, skin symptoms, and lacrimation were significantly more frequent in Arm B compared with Arm A as shown in Table 4. Considering the results above, we chose Arm A as the better treatment arm.

- Anti PD-L1 therapies are introduced for the study population/in the market during the study conduct. Were any of the patients received anti-PD-L1 in this study? Was there any data collected related to the use of anti PD-L1 therapy(ies)?

**Response.**

We had 33 patients who had the recurrence in 101 enrolled patients. Among them, 3 patients received anti PD-1 antibody nivolumab. However, we do not have the detailed data related the use of nivolumab. We have no patients who received anti PD-L1 antibody therapy.

- With the existence of new anti PD-1/L1 in this patient population, how do you see the possibility of running phase III study based on the current study?

**Response.**

We designed the current study in 2012, when time background about chemotherapeutic agents was different from the present day. Fluoropyrimidine agents including UFT and S-1 are inexpensive in drug price and have better tolerability. Thus, we consider that S-1 is still valuable if we select appropriate patients for it. However, as the spread use of new anti PD-1/L1antibodies in this patient population, there may be low possibility of running the phase III study if we plan S-1 monotherapy. A previous study designed phase III trial comparing S-1 plus carboplatin with paclitaxel plus carboplatin in chemotherapy-naïve patients with advanced NSCLC (Okamoto et al. JCO 2010). Based on such a concept, we may be able to design the treatment arm of the combination of S-1 and new anti PD-1/L1antibodies.

- Was subset analysis of survival in page 357 pre-planned in the protocol or in SAP? If not why was this analysis chosen?

**Response.**

As mentioned in the section 12.2. of the study protocol (attached as Supporting information), differences in RFS and OS stratified by the treatment completion rate were planned. Regarding the other factors used for the stratification of subset analysis of survival performed in this article, they were not pre-planned in the protocol. Besides the treatment completion rate, we focused on pStage, *EGFR* mutational status, smoking history and sex for subset analysis of survival, as those are considered to be prognostic factors.

- The statement about not significantly difference in this article, when analyzing secondary endpoints and particularly when in the subgroup analyses should be interpreted carefully. In addition to small sample size, multiple comparisons that may require adjustment of type 1 error, the study was not powered for those secondary endpoints and any statement related significant/non-significant would potentially be misleading.

**Response.**

We thank the Reviewer #1 for his/her valuable comment. We recognized that this point is extremely critical. Thus, we added the following description based on the comment by Reviewer #1 as a limitation in Line 485-490 in “Discussion”:

For analyzing secondary endpoints RFS and OS, particularly in the subset analyses, the results should be interpreted carefully. Because of the small sample size, in multiple comparisons that may require adjustment of type 1 error, the current study does not have power for those secondary endpoints and any statement related to the significant/non-significant results would potentially be misleading.

Minor:

- Relapse free and recurrence free survival used in this article while presumably means the same thing. Please confirm and if so, please choose 1 term consistently throughout the article

**Response.**

As the Reviewer #1 pointed out, we had used the both words “relapse-free survival” and “recurrence-free survival” as the same meaning. We chose “recurrence-free survival” consistently throughout the article. We changed “relapse” to “recurrence” in Line 93 and deleted “relapse-free survival” in Line 275.

We also revised the related section in the study protocol of both English and Japanese version and atttached them as the revised files (page 29 in “SLCG1201 protocol ver1.8 English version revised” and page 26 in “SLCG1201 protocol ver1.8 Japanese version revised”).

- Reference on randomized phase II selection design, please use the original paper of Simon et al.(1985 *)

*R Simon, R E Wittes, S S Ellenberg : Randomized phase II clinical trials

Cancer Treat Rep. 1985 Dec;69(12):1375-81.

**Response.**

We added the paper written by Simon et al. for the reference on randomized phase II selection design, instead of the paper written by Rubinstein et al (reference number: 21). We highlighted the reference number 21 in yellow in Lines 255 and 633.

- P-values: when less than 0.001 should simply be stated (p < 0.001)

**Response.**

We changed the p value less than 0.001 to “< 0.001” in Table 3 and Supporting information (S6 Fig B and S7 Fig B). We also changed “*P*” to “*p*” for p values throughout the revised article including the revised manuscript with track changes and Supporting information.

- Toxicity: as this a phase II study, would suggest that toxicity table should be included in the main body (not as supplementary)

**Response.**

We included “S7 Table” in the original version into the main body and thus “S7 Table” was renamed as “Table 4”. We also created “new” S7 Table based on the first comment from Reviewer #2. S8-S11 Tables in the original version were not renamed.

***Comments by Reviewer #2 and our responses to each comment***

Reviewer #2: Authors present results from a Phase II clinical trial of 1-year treatment with S-1 in elderly patients with completely resected pathological stage IA to IIIA non-small cell lung cancer (NSCLC), comparing two different cycles of treatment: 1) alternate-day oral administration of S-1 for 4 days a week; and 2) daily administration of S-1 for 2 weeks followed by a 1 week-rest (each cycle is repeated for the entire year.) Authors report on feasibility, safety and preliminary assessments of efficacy. The manuscript will be strengthened if the authors consider the following points.

1. Figure 1 is a CONSORT diagram. Ideally it would include a box before the 1st box, which shows how many people were considered for eligibility before enrollment. It seems highly unlikely that authors only considered 101 individuals for the study, and they all met inclusion/exclusion criteria.

**Response.**

We are grateful for the comments from Reviewer #2. We reviewed the candidate cases for the current study in the study period (from May 2012 to April 2016) in each participating institution. Namely, we reviewed the cases of the elderly patients (75 years old or higher) with completely resected pathological stage IA (tumor diameter > 2 cm) - IIIA of NSCLC undergoing lobectomy with mediastinal lymph node dissection. Although we were not able to obtain the number of the candidate cases from 3 out of 19 participating institutions, we got it from the other institutions. We show the number of enrolled and candidate cases in “new” S7 Table. Among 16 institutions that had the data of candidate cases, 94 cases were enrolled from 872 cases. Regarding the non-enrolled cases, we were not able to obtain the data of the reason why candidate cases were not enrolled in the current study.

We added the following sentences in Line 304-307 in “Patient characteristics” in “Results”:

The number of enrolled and candidate cases in each participating institution is shown in S7 Table. We were not able to obtain the number of candidate cases from 3 out of 19 participating institutions. Among 16 institutions that had the data of candidate cases, 94 cases were enrolled from 872 cases.

2. A primary outcome being assessed is RDI which is the ratio of actual total administration dose and planned total administration dose. However, in the discussion, they mention a limitation of no pill counts. What is then meant by "actual total administration dose"?

**Response.**

We are sorry about ambiguous expression. “Actual total administration dose” is based on the self-reported dose of the patients who took medication of S-1. We or patients did not exactly count the remaining pills.

We revised the following sentence in Line 247-249 in “Study design and statistical analysis” in “Patients and Methods”:

RDI was defined as the rate between the actual total administered~~ration~~ dose (based on the self-reported dose of the patients who took medication of S-1) and the planned total administered~~ration~~ dose.

Minor points:

1. I was confused by S1 Fig until I read the protocol, since I did not see mention in the text of the manuscript that S-1 was administered twice a day. Authors should state this in the main text, since that seems to be an important detail about the administration of the treatment. This may not be known by all readers.

**Response.**

We added the following sentence in Lines 209-210 in the section of “Treatment and follow-up” in “Patients and Methods”:

Patients received S-1 twice per day in the morning and in the evening, by equally dividing the amount of administration per day into two.

2. Authors should specifically state in the statistical analysis section that they are performing "intent to treat" analyses for the survival outcomes.

**Response.**

We added the phrase of “as intent-to-treat analyses” in Line 282 in the section of “Study design and statistical analysis” in “Patients and Methods”.

3. In general, I would recommend rounding p-values - 3 digits beyond the decimal are a bit excessive, especially given the sample size. 1 or 2 non-zero digits beyond the decimal should be sufficient.

**Response.**

We changed the description of p value from 3 digits to 2 digits beyond the decimal throughout the article.

4. lines 331-332: authors already mentioned the lack of treatment related deaths in lines 327-328.

**Response.**

We deleted the sentence “There were no treatment-related deaths in either group during treatment.” in Line 361-362 in the section of “Toxicity” in “Results”.

5. line 345: I suspect "monitored for at least 5 years from the registration" needs some clarification. The range of follow-up time was 6.2 to 103.9 months, so not everyone was followed for 5 years.

**Response.**

We are sorry about ambiguous expression. We meant that all censored cases were monitored for at least 5 years from the registration. We added the phrase “until death or” in Line 377 in the section of “Survival” in “Results”. Clarified sentence is as follows:

Patients who received the protocol treatment (n = 97) and all enrolled patients (n = 101) were monitored until death or for at least 5 years from the registration.

6. In the "Subset analysis of survival" section, authors should clarify which figures actually correspond to their statements. For example, lines 357-359 refer the reader to S3 Fig A-D, yet the sentence really only talks about S3 Fig B & D. Authors do not seem to mention the results of S3 Fig A & C.

**Response.**

We are sorry that we did not mention all the detailed results for subset analysis of survival. We added and revised the sentences in Lines 389-396, 404-409 and 415-416 in the section of “Subset analysis of survival” in “Results” as follows:

In Lines 389-396:

Regarding the impact of the treatment completion rate of S-1 administration at 6 months, it seems that RFS and OS were better in the cases with the completion of S-1 administration (S3 Fig A, C). However, there were no significant differences of RFS or OS if excluding the recurrent cases within 6 months (S3 Fig B, D, 6 cases were excluded). For the group with the completion of S-1 administration at 6 months, there were no significant differences of RFS or OS (S3 Fig E-F). For the group without the completion of S-1 administration at 6 months, RFS and OS tended to be worse in Arm A compared to Arm B. (S3 Fig G-J).

In Lines 404-409:

As for RFS, there were no significant differences between Arm A and Arm B in the patients with pStage IA/IB (S4 Fig C) and with pStage IB (S4 Fig D). As for OS, Arm A was worse in the patients with pStage IA/IB (S4 Fig E), especially with pStage IB (S4 Fig F). There were no significant differences of RFS or OS between Arm A and Arm B in the patients with pStage IIA/IIB or IIIA (S4 Fig G-J).

In Line 415-416:

Regarding *EGFR* mutational status, there was no significant difference in RFS (S5 Fig A), whereas OS was ~~worse~~better in the cases with~~out~~ *EGFR* mutation (S5 Fig B).

Regarding S5 Fig A&B, we corrected the caption of “*EGFR* wild type” to “*EGFR* wild type or unknown”, as there is one squamous cell carcinoma case, in which *EGFR* mutational status was unknown, in the group of “*EGFR* wild type”, as shown in Table 1.

**Additional changes in the revised article**

We shortened the full title of the manuscript as follows because the character limit of the title is 250 characters.

**Randomized phase II study of daily versus alternate-day administrations of S-1 for the elderly patients with completely resected pathological stage IA (tumor diameter > 2 cm) - IIIA of non-small cell lung cancer: ~~results of the~~ Setouchi Lung Cancer Group Study 1201 ~~(SLCG1201)~~**

We added the following sentence at the end of author affiliations (Line 44): All the authors are the members of Setouchi Lung Cancer Group.

We removed funding statement from the manuscript, as PLOS ONE editorial staff said that funding information should not appear in any areas of the manuscript.

As we created “new” S7 Table, the caption of it in Line 727-728 was revised as follows:

**S7 Table.** Enrolled and candidate cases for SLCG1201 study in each participating institution.

We corrected *EGFR* mutational status in case #76 from “negative” to “unknown” in Excel files “SLCG1201 Patient data revised” and “SLCG1201 Patient data 2 revised” (textual error).
