# Supplementary material for: Randomized phase II study of daily versus alternate-day administrations of S-1 for the elderly patients with completely resected pathological stage IA (tumor diameter > 2 cm)—IIIA of non-small cell lung cancer: Setouchi Lung Cancer Group Study 1201
Source: PLoS One. 2023 May 19;18(5):e0285273. doi: 10.1371/journal.pone.0285273 (PMC10198543; doi:10.1371/journal.pone.0285273)
Supplement: Attachment — Submitted filename: Yamamoto et al PONE-D-22-18798R1 Responses to the Reviewers.docx [file pone.0285273.s029.docx]

**PONE-D-22-18798R1**

**Yamamoto et al.**

**Randomized phase II study of daily versus alternate-day administrations of S-1 for the elderly patients with completely resected pathological stage IA (tumor diameter > 2 cm) - IIIA of non-small cell lung cancer: Setouchi Lung Cancer Group Study 1201**

**Responses to the Reviewers**

We thank the reviewers and the editor for their careful and conscientious reading of the manuscript and for their useful suggestions that have resulted in a much-improved manuscript. As indicated in the responses that follow, we have taken the comments and suggestions into account as much as possible in the revised version of our paper. The lines we indicate in the following comments are based on the revised manuscript with track changes.

***Comments by Reviewer #1 and our responses to each comment***

Reviewer #1: Thanks for your response. Concerning the following:

- Treatment completion was 69.4% in Arm A and 64.6% in Arm B yet the study conclusion: Alternate-day oral administration of S-1 was demonstrated to be feasible with low toxicity in elderly patients with completely resected NSCLC solely focused on Arm A. Why was treatment B not mentioned to be feasible? Since both treatment arms passed the criteria to be feasible, was there any rule for choosing the better treatment Arm applied?

From your answer:

“ …

As for the treatment completion rate, Table 3 shows that the completion rate at 12 months in Arm A (63.3%) is more than 15% higher than that in Arm B (45.8%). “

This question is not answered appropriately. The primary endpoint (used to power the study) is the completion rate at 6 months and what you quoted here is the treatment completion rate at 12 months. You need to have your decision concerning feasibility of the study be based on your primary endpoint. The original question still needs to be addressed.

In addition, you should stick with the below rule, keeping in mind your primary endpoint as stated above. In this case treatment arm A and B both are successful wrt the primary endpoint and the difference between the 2 is much less than 15%.

“If the completion rate in one group is greater than 40% and the difference between one group and the other is within 15%, the two groups will not be directly compared based on retention rate numbers alone, but rather on toxicity, quality of life, convenience, cost, retention rate after 6 months, recurrence-free survival, and survival rate to determine which is more suitable as S-1 therapy in future Phase III trials.”

The following sentence in the discussion and throughout the manuscript should be corrected

As shown in Table 2, RDI of S-1 at 12 months is significantly better in Arm A than in Arm B. As for the treatment completion rate, Table 3 shows that the completion rate at 12 months in Arm A (63.3%) is more than 15% higher than that in Arm B (45.8%) ….

**Response.**

We are grateful for the comments from Reviewer #1. Based on the results of the primary endpoint, we corrected the description in the article.

We revised “Conclusion” in “Abstract” as follows:

**Conclusion:** Both alternate-day and daily oral administrations of S-1 were demonstrated to be feasible, a~~A~~lthough alternate-day oral administration of S-1 was ~~demonstrated to be feasible with~~advantageous for better treatment completion at 12 months and low toxicity in elderly patients with completely resected NSCLC.

We revised the following sentences in Line 347-354 in “Feasibility” of “Results”:

The treatment completion rate based on an RDI of 70% or more for 6 months in Arm A was greater than 40%~~. However,~~  and it did not exceed that of Arm B by more than 15%, indicating that both Arm A and Arm B were feasible. To determine which is more suitable as S-1 therapy in future Phase III trials, the comparison between the two groups should not be performed with the treatment completion rate alone, but rather with toxicity, quality of life, convenience, cost, treatment completion rate after 6 months, RFS, and OS ~~to determine which is more suitable as S-1 therapy in future Phase III trials~~.

We revised the following sentence in Line 450-452 in “Discussion”:

In the current study, both alternate-administration of S-1 (Arm A) and ~~was superior to~~ daily administration (Arm B) were feasible in terms of the treatment completion at 6 months, whereas there is a trend of unfavorable prognosis in Arm A.

We revised the following sentence in Line 471-474 in “Discussion”:

In clinical practice, which treatment arm to select for elderly patients with completely resected NSCLC depends on the physician’s discretion in consideration of ~~Considering~~ the results above~~, we chose Arm A as the better treatment arm~~.

We revised the following sentence in Line 494-497 in “Discussion”:

In conclusion, both alternate-day and daily oral administrations of S-1 ~~was~~ were demonstrated to be feasible ~~with low toxicity~~ as adjuvant chemotherapy in elderly patients with stage IA to IIIA NSCLC, although alternate-day oral administration of S-1 was advantageous for better treatment completion at 12 months and low-toxicity**.**

**Additional changes in the revised article**

None
