# Supplementary material for: Randomized phase II study of daily versus alternate-day administrations of S-1 for the elderly patients with completely resected pathological stage IA (tumor diameter > 2 cm)—IIIA of non-small cell lung cancer: Setouchi Lung Cancer Group Study 1201
Source: PLoS One. 2023 May 19;18(5):e0285273. doi: 10.1371/journal.pone.0285273 (PMC10198543; doi:10.1371/journal.pone.0285273)
Supplement: Attachment — Submitted filename: Yamamoto et al PONE-D-22-18798R2 Responses to Reviewers.docx [file pone.0285273.s030.docx]

**PONE-D-22-18798R2**

**Yamamoto et al.**

**Randomized phase II study of daily versus alternate-day administrations of S-1 for the elderly patients with completely resected pathological stage IA (tumor diameter > 2 cm) - IIIA of non-small cell lung cancer: Setouchi Lung Cancer Group Study 1201**

**Responses to Journal Requirements**

Please review your reference list to ensure that it is complete and correct. If you have cited papers that have been retracted, please include the rationale for doing so in the manuscript text, or remove these references and replace them with relevant current references. Any changes to the reference list should be mentioned in the rebuttal letter that accompanies your revised manuscript. If you need to cite a retracted article, indicate the article’s retracted status in the References list and also include a citation and full reference for the retraction notice.

**Response.**

We reviewed our reference list and confirmed that it was complete and correct. We did not cite any papers that have been retracted.

**Responses to the Reviewers**

We thank the reviewers and the editor for their careful and conscientious reading of the manuscript and for their useful suggestions that have resulted in a much-improved manuscript. As indicated in the responses that follow, we have taken the comments and suggestions into account as much as possible in the revised version of our paper. The lines we indicate in the following comments are based on the revised manuscript with track changes.

***Comments by Reviewer #3 and our responses to each comment***

Reviewer #3: The study compared two different dose of S-1 for elderly NSCLC stage IA patients after surgery. The statistical design was acceptable, and the data were analyzed properly. The study showed that alternative schedule of S-1 may have similar efficacy to standard dose of S-1, with more tolerable toxicity. The result is also important for other indications of S-1, especially for elderly patients.

One question for the author: since UFUR is the only drug approved for the indication of adjuvant therapy for NSCLC, why did the author not consider using UFUR in two different dosing schedule?

**Response.**

We are grateful for the comments from Reviewer #3. UFT has low toxicity compared to S-1. Daily administration of UFT has been already established and thus, we did not consider the study using UFT in two different dosing schedules.

***Comments by Reviewer #5 and our responses to each comment***

Reviewer #5:

1. "the threshold 6-month treatment completion rate for the current protocol in both groups was 40%' - seems to be an extremely low threshold for adjuvant treatment set. Shouldn't a higher threshold be more clinically meaningful? eg 50% at least.

**Response.**

We are grateful for the comments from Reviewer #5. The objective of the current study is elderly patients, and we considered that the treatment completion rate of S-1 in elderly patients was lower than the patients younger than 75 years old. Although we set the treatment completion rate of S-1 as 60% in our previous study targeting the patients younger than 75 years old (SLCG1301, Ref. 26), we assumed that the threshold 6-month treatment completion rate for the current protocol in both groups was 40%.

2. 1) If the 6-month treatment completion rate is 40% or less in both groups, the protocol treatments for both groups is considered not promising for postoperative adjuvant chemotherapy - why is 6-month completion rate used as primary endpoint, when the adjuvant treatment is for a duration of one year?

**Response.**

The objective of the current study is elderly patients, and we initially considered the possibility that only small number of cases continued the administration of S-1 for one year. Thus, we set the treatment completion rate at 6 months as the primary endpoint.

3. EGFR mutation was detected in 31 (30.7%) patients - looks like a big confounding factor, were these patients treated with osimertinib?

**Response.**

*EGFR* mutational status was one of the adjustment factors for randomization and thus there is no difference in the frequency of *EGFR* mutations in both arms. We had 33 patients who had the recurrence in 101 enrolled patients. Among 33 patients, 10 patients had *EGFR* mutations and 2 patients received osimertinib. As for other EGFR-TKIs, 4 patients received gefitinib, 1 patient received afatinib, and 1 patient received erlotinib.

4. Median actual total administered dose of S-1 was 17340 mg for Arm A and 17640 md (typo) for Arm B, respectively (p = 0.82)

**Response.**

This is just the description in “Feasibility” of “Results” of the main text, and there are no further comments or questions from Reviewer #5.

5. To determine which is more suitable as S-1 therapy in future Phase III trials, the comparison between the two groups should not be performed with the treatment completion rate alone, but rather with toxicity, quality of life, convenience, cost, treatment completion rate after 6 months, RFS, and OS - given the "toxicities were generally well tolerated in both groups and there were no grade 4 adverse events", and numerically lower RFS (56.9% vs 65.7%) and OS (68.6% vs 82.0%) with the arm A, why did the authors come to a conclusion that arm A is 'more advantageous due to lower toxicity'? The numerical difference in survival rate is huge (and sample not adequate to get a low p value).

Another conclusion should be derived, which is "the experimental regime (arm A) has not been demonstrated to be a better alternative to the standard daily administration of TS1, and daily dosing remains the standard of care."

**Response.**

Regarding OS and RFS, there is a trend of unfavorable prognosis in Arm A. However, there is no statistical difference between them. As described in Discussion, disease-specific survival showed no difference in both arms, and Arm A had male and ever smoker cases more frequently compared to Arm B. They may have affected the trend of unfavorable prognosis in Arm A.

Although toxicities were generally well tolerated in both groups, anorexia, skin symptoms, and lacrimation, which are the typical adverse effects that can affect the patients’ quality of life, were significantly more frequent in Arm B compared with Arm A.

As described in Discussion, there is no standard care of postoperative adjuvant chemotherapy for elderly patients with completely resected NSCLC. Thus, “daily dosing remains the standard of care” raised by Reviewer #5 as another conclusion, is not appropriate.

Considering above, we deleted the phrase “although alternate-day oral administration of S-1 was advantageous for better treatment completion at 12 months and low toxicity” and put the phrase of “Although several adverse effects were less frequent in Arm A,” and the following are the final version of Conclusion in Abstract and Discussion in the main text.

In Abstract (Lines 76-80):

**Conclusion:** Although several adverse effects were less frequent in Arm A, b~~B~~oth alternate-day and daily oral administrations of S-1 were demonstrated to be feasible~~, although alternate-day oral administration of S-1 was advantageous for better treatment completion at 12 months and low toxicity~~ in elderly patients with completely resected NSCLC.

In Discussion (Lines 493-499):

In conclusion, although several adverse effects were less frequent in Arm A, both alternate-day and daily oral administration of S-1 were demonstrated to be feasible as adjuvant chemotherapy in elderly patients with stage IA to IIIA NSCLC~~, although alternate-day oral administration of S-1 was advantageous for better treatment completion at 12 months and low toxicity~~. Although there is no standard care of postoperative adjuvant chemotherapy for elderly patients with completely resected NSCLC, future investigation should be focused on high-risk populations for recurrence.
